# Supplementary material for: Image3C, a multimodal image-based and label-independent integrative method for single-cell analysis
Source: eLife. 2021 Jul 21;10:e65372. doi: 10.7554/eLife.65372 (PMC8370771; doi:10.7554/eLife.65372)
Supplement: Supplementary file 1. — Names and descriptions of the features quantified by IDEAS software and used for clustering events based on cell morphology in the homeostasis cell composition experiment. BF: brightfield; CI: cell intrinsic; CF: cell function. [file elife-65372-supp1.docx]

**Supplementary File 1: Features used for the morphology assay**

Names and descriptions of the features quantified by IDEAS software and used for clustering events based on cell morphology in the homeostasis cell composition experiment. BF is Bright Field, CI is Cell Intrinsic, CF is Cell Function.

| **ID** | **Feature Name_**  **ImageMask_Channel** | **CI or CF** | **Feature description** |
| --- | --- | --- | --- |
| 1 | Area_AdaptiveErode_BF | CI | Cell size |
| 2 | Area_Intensity_SSC | CI | Areas of SSC signal above background |
| 3 | Area_Morphology_Draq5 | CI | Area of DNA signal |
| 4 | Aspect.Ratio_AdaptiveErode_BF | CI | Aspect ratio of total cell area |
| 5 | Bright.Detail.Intensity.R3_AdaptiveErode_BF_BF | CI | Intensity of brightest staining areas |
| 6 | Bright.Detail.Intensity.R3_AdaptiveErode_BF_SSC | CI | Intensity of brightest signal areas |
| 7 | Bright.Detail.Intensity.R3_AdaptiveErode_BF_Draq5 | CI | Intensity of brightest staining areas |
| 8 | Circularity_AdaptiveErode_BF | CI | Circularity of whole cell shape |
| 9 | Circularity_Morphology_Draq5 | CI | Circularity of nucleus |
| 10 | Contrast_AdaptiveErode_BF_BF | CI | Large changes in pixel values - Granularity of signal |
| 11 | Contrast_AdaptiveErode_BF_SSC | CI | Large changes in pixel values - Granularity of signal |
| 12 | Diameter_AdaptiveErode_BF | CI | Diameter of whole cell shape |
| 13 | Diameter_Morphology_Draq5 | CI | Diameter of nucleus |
| 14 | H.Energy.Mean_AdaptiveErode_BF_BF | CI | Intensity concentration - Texture feature |
| 15 | H.Energy.Mean_Morphology_Draq5_Draq5 | CI | Intensity concentration - Texture feature |
| 16 | H.Entropy.Mean_AdaptiveErode_BF_BF | CI | Intensity concentration and randomness of signal - Texture feature |
| 17 | H.Entropy.Mean_Morphology_Draq5_Draq5 | CI | Intensity concentration and randomness of signal - Texture feature |
| 18 | Intensity_AdaptiveErode_BF_SSC | CI | Integrated intensity of signal within whole cell mask - Cell granularity |
| 19 | Intensity_AdaptiveErode_BF_Draq5 | CI | Integrated intensity of signal within whole cell mask |
| 20 | Lobe.Count_Morphology_Draq5 | CI | Number of lobes of nucleus |
| 21 | Max.Pixel_Intensity_SSC | CI | Maximum pixel intensity within a whole cell mask - Cell granularity |
| 22 | Max.Pixel_Morphology_Draq5 | CI | Maximum pixel intensity within a whole cell mask |
| 23 | Mean.Pixel_Morphology_Draq5 | CI | Mean pixel intensity within a whole cell mask |
| 24 | Shape.Ratio_AdaptiveErode_BF | CI | Minimum thickness divided by length - Cell shape |
| 25 | Std.Dev_AdaptiveErode_BF | CI | Standard deviation of BF signal - Cell granularity and variance in BF |
