# Supplementary material for: Image3C, a multimodal image-based and label-independent integrative method for single-cell analysis
Source: eLife. 2021 Jul 21;10:e65372. doi: 10.7554/eLife.65372 (PMC8370771; doi:10.7554/eLife.65372)

Zebrafish
Homeostasis
Cell Morphology

Cell Gallery

Cluster
*Dr*1

Lymphocytes / Progenitors

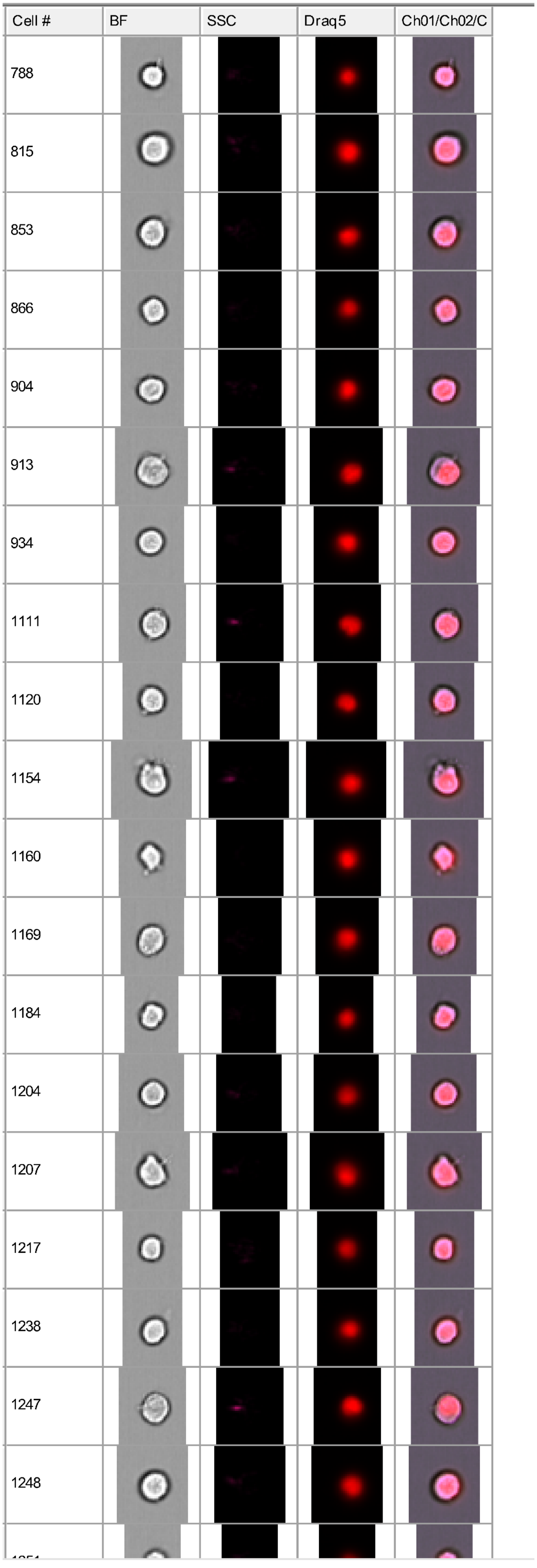

Cluster
Dr2

Dead cells

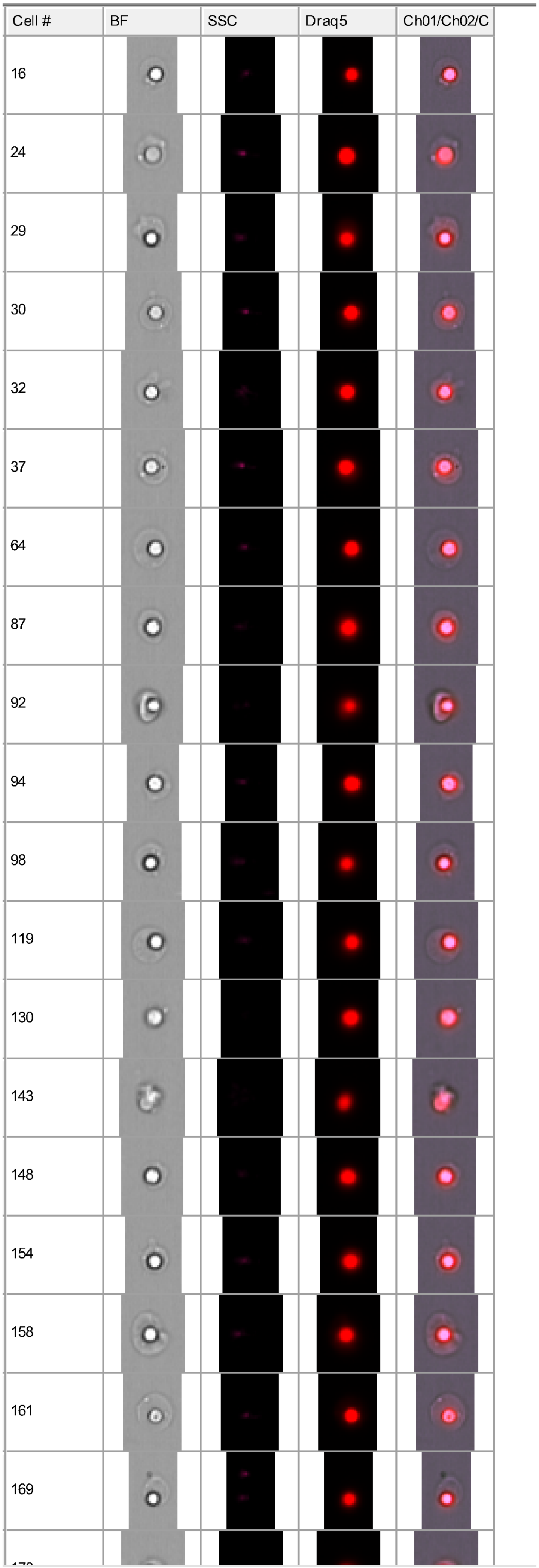

Cluster
Dr3

Dead cells

Cell #	BF	SSC	Draq5	Ch01/Ch02/C
46				
166				
173				
175				
190				
212				
283				
340				
367				
641				
693				
711				
938				
947				
1118				
1165				
1175				
1229				
1370				
[illegible]				

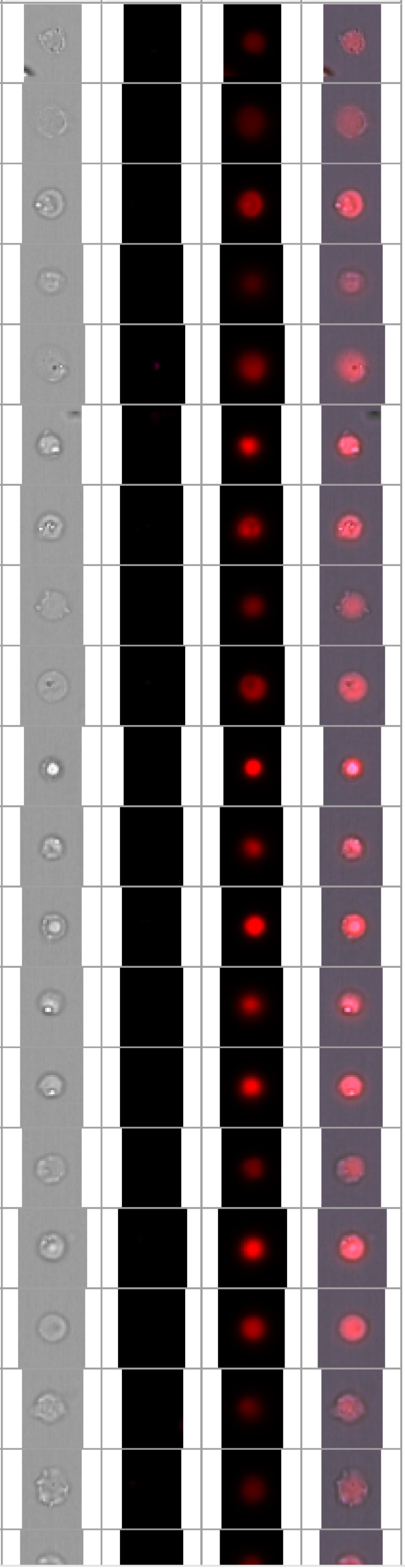

Cluster
*Dr*4

Dead cells

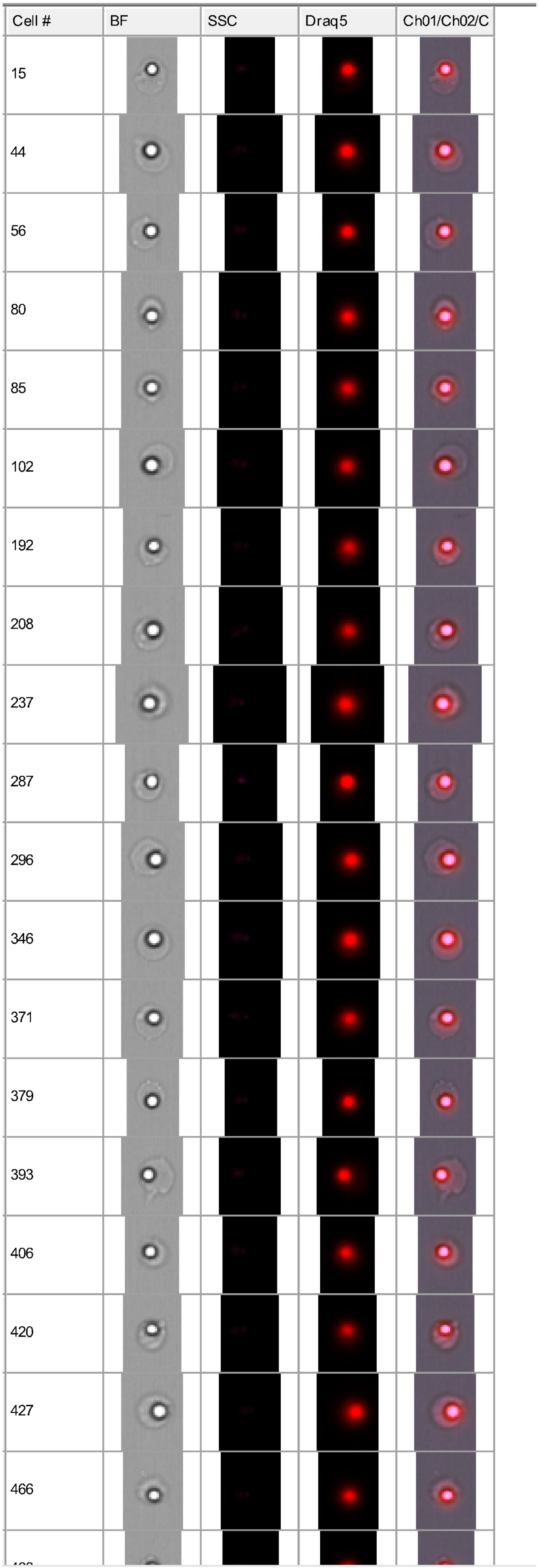

Cluster
Dr5

Dead cells

Cell #	BF	SSC	Draq5	Ch01/Ch02/C
7				
11				
12				
33				
53				
58				
76				
89				
93				
105				
122				
176				
188				
204				
214				
225				
248				
280				
285				
[illegible]				

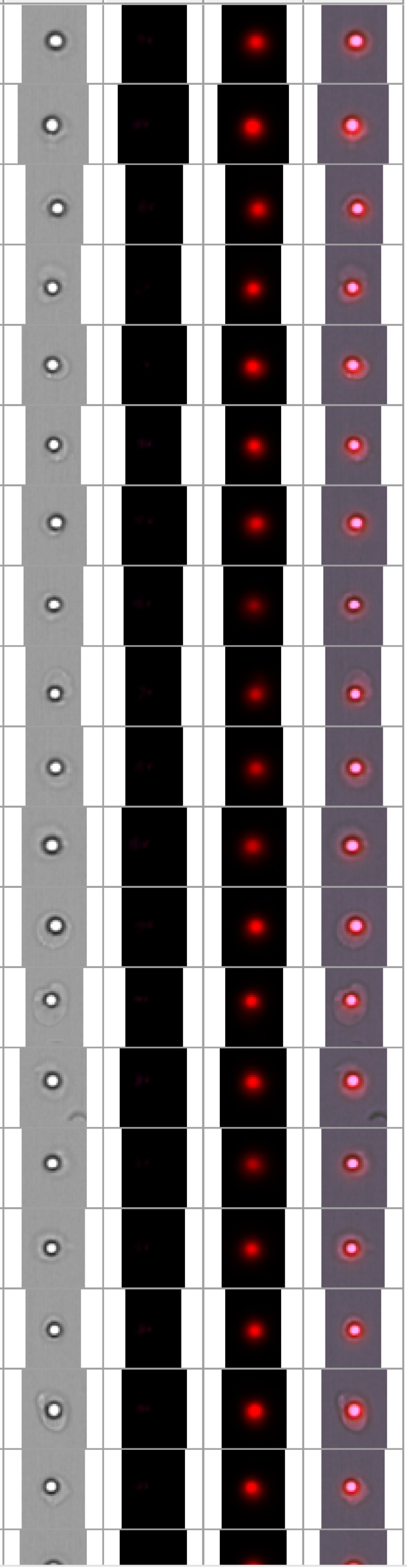

Cluster
*Dr*6

Erythrocytes

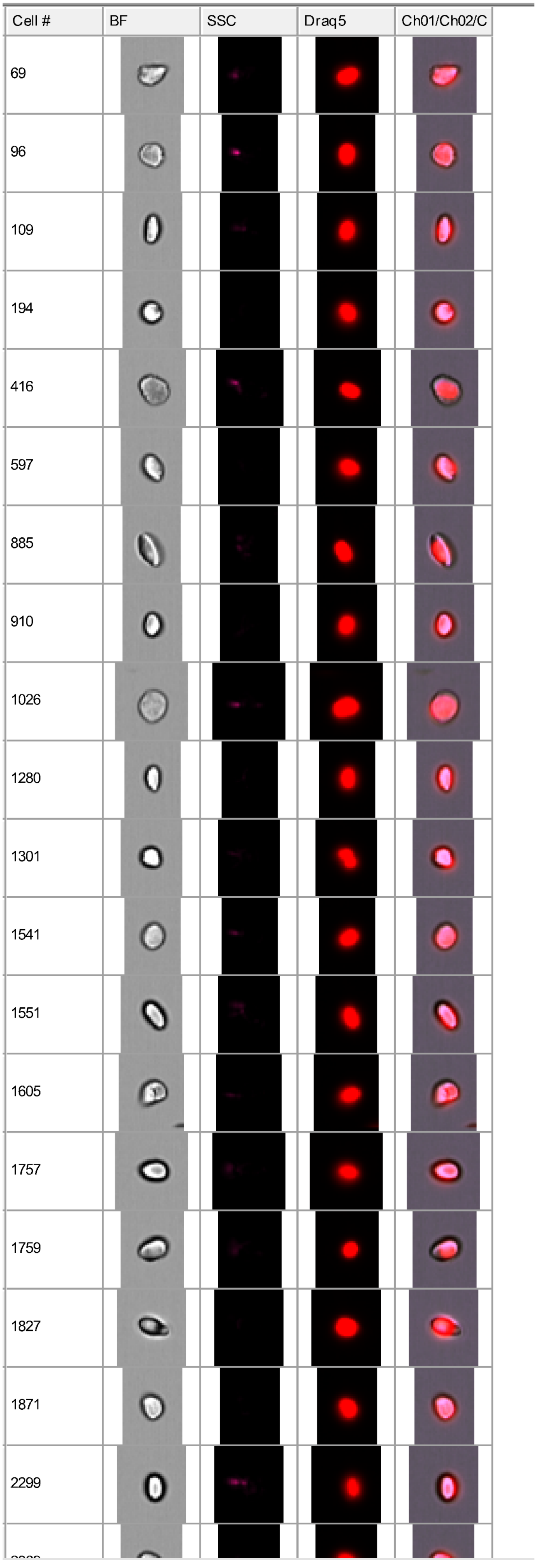

Cluster
Dr7

Lymphocytes / Progenitors

Cell #	BF	SSC	Draq5	Ch01/Ch02/C
2199				
2203				
2211				
2214				
2228				
2230				
2244				
2253				
2255				
2284				
2287				
2289				
2298				
2305				
2311				
2324				
2333				
2336				
2337				
[illegible]				

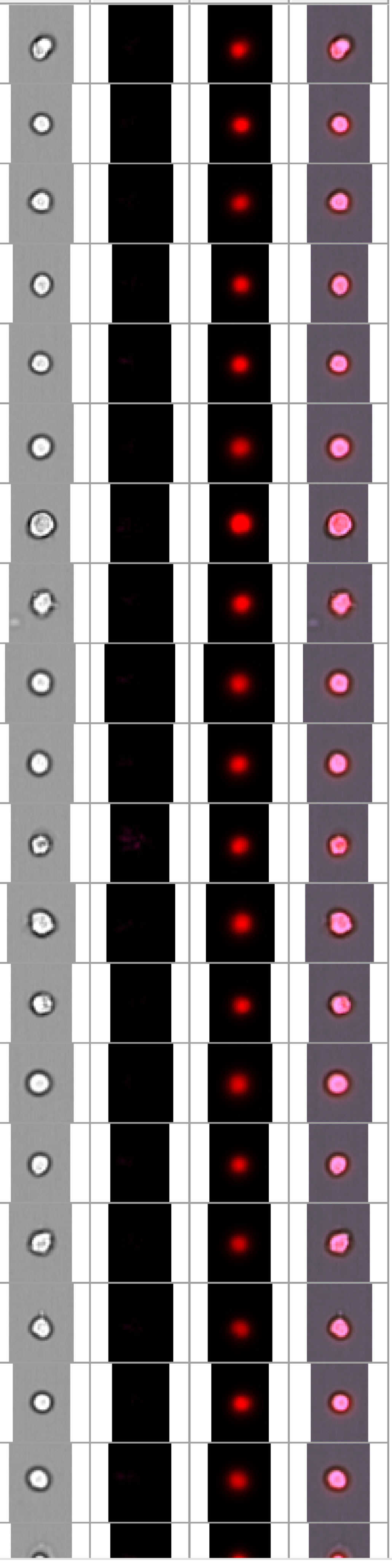

Cluster
*Dr*8

Promyelocytes

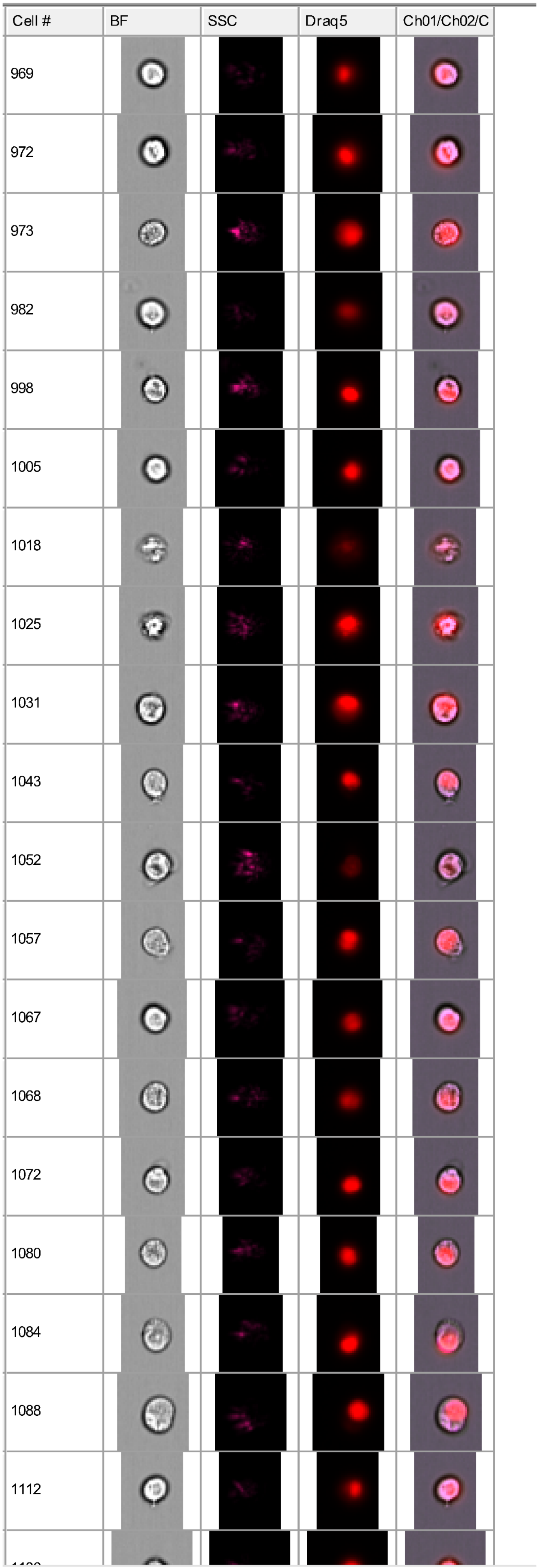

Cluster
*Dr*9

Debris

Cell #	BF	SSC	Draq5	Ch01/Ch02/C
40				
118				
317				
908				
952				
1252				
1434				
1516				
1529				
1638				
1950				
2036				
2186				
2541				
2718				
2998				
3478				

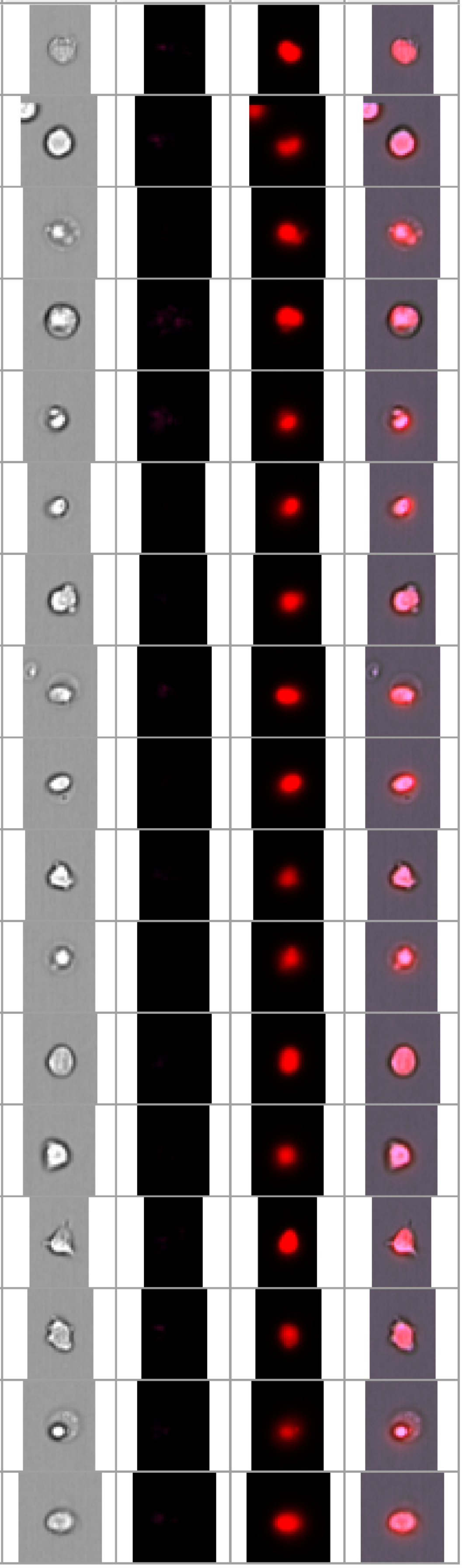

Cluster
*Dr*10

Neutrophils

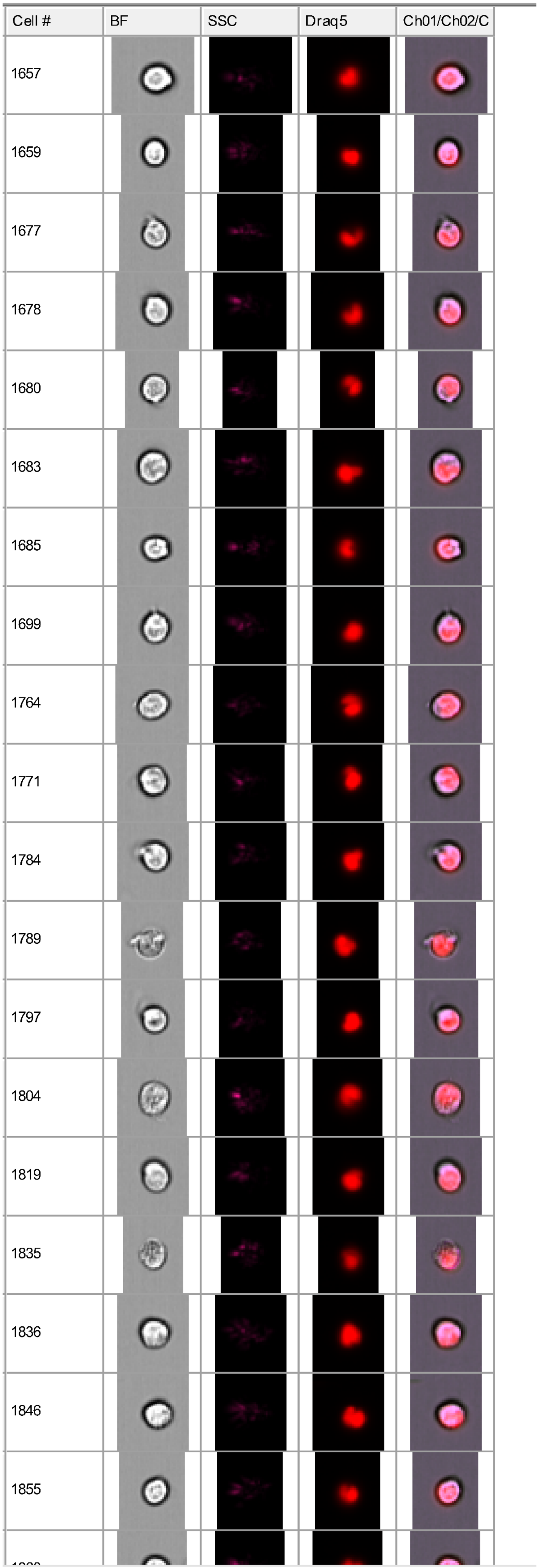

Cluster
*Dr*11

Myeloid Precursors

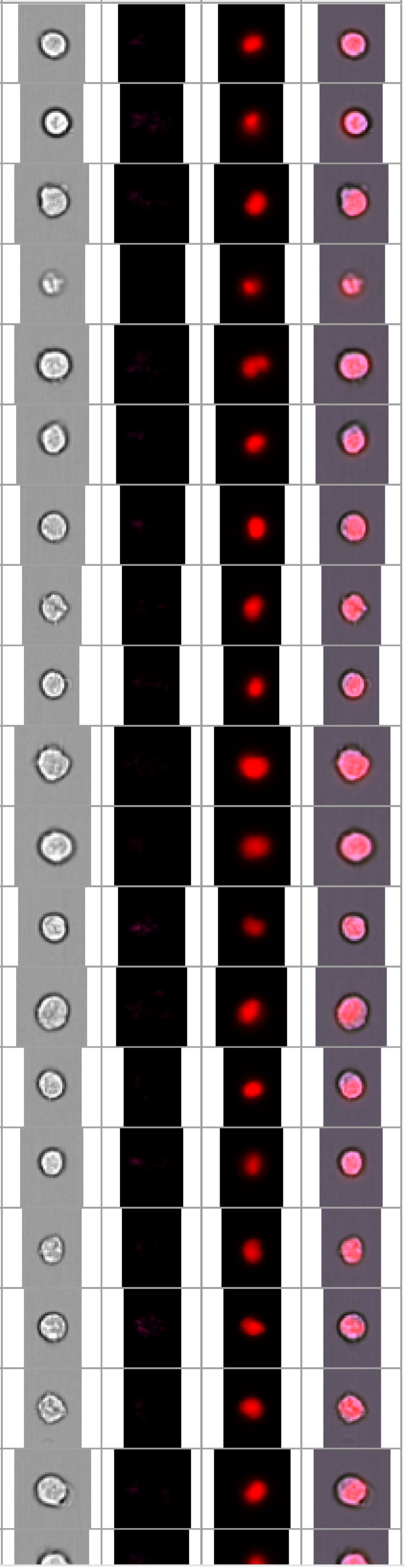

Cell #	BF	SSC	Draq5	Ch01/Ch02/C
1335				
1355				
1476				
1479				
1540				
1594				
1614				
1624				
1634				
1721				
1723				
1740				
1807				
1822				
1838				
1929				
1946				
1954				
2043				
[illegible]				

Cluster
*Dr*12

Dead cells

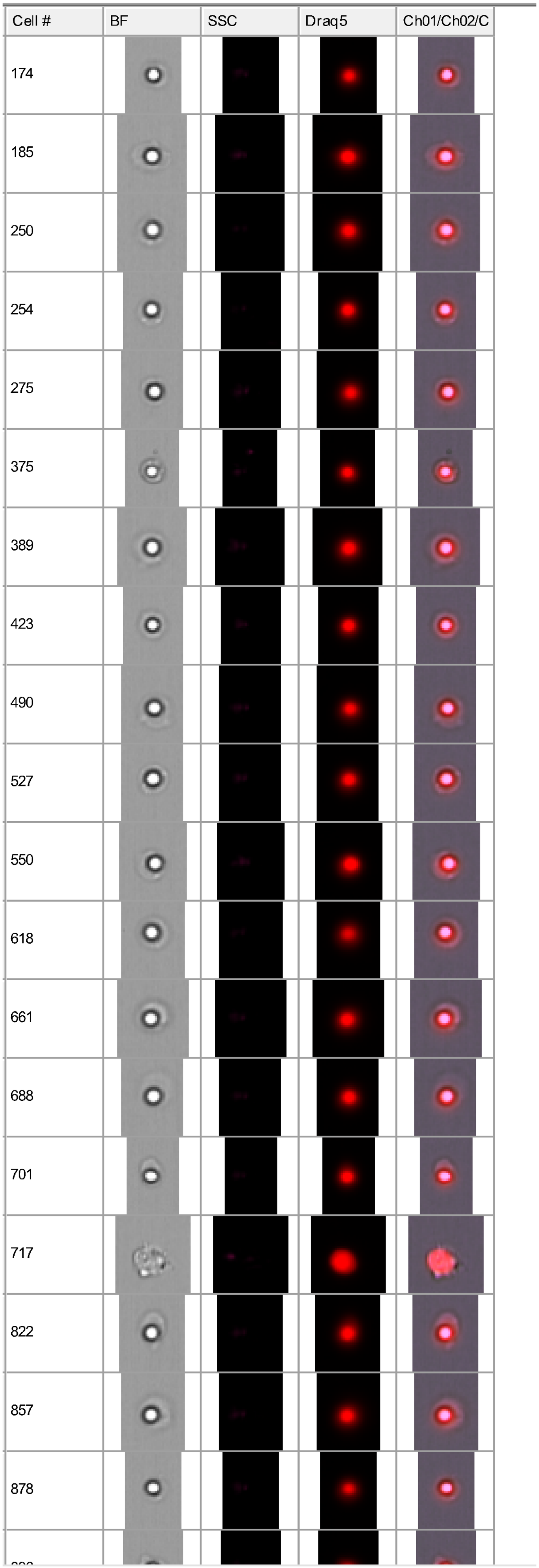

Cluster
*Dr*13

Dead cells

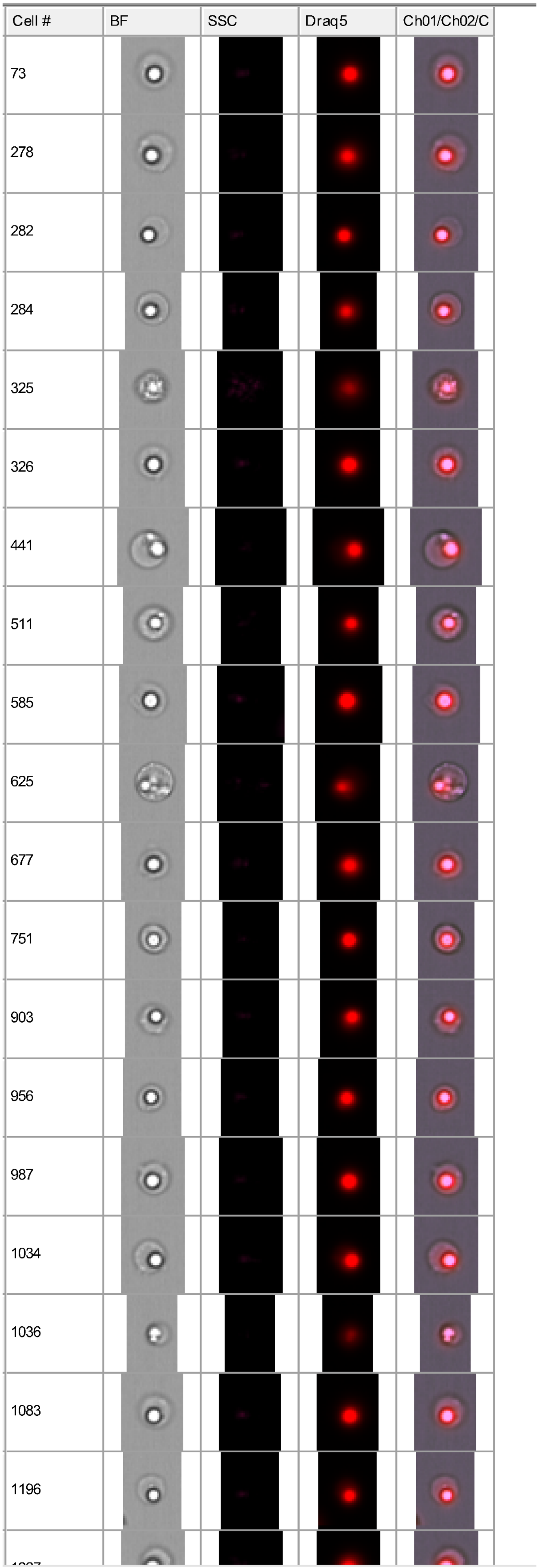

Cluster
*Dr*14

Erythrocytes

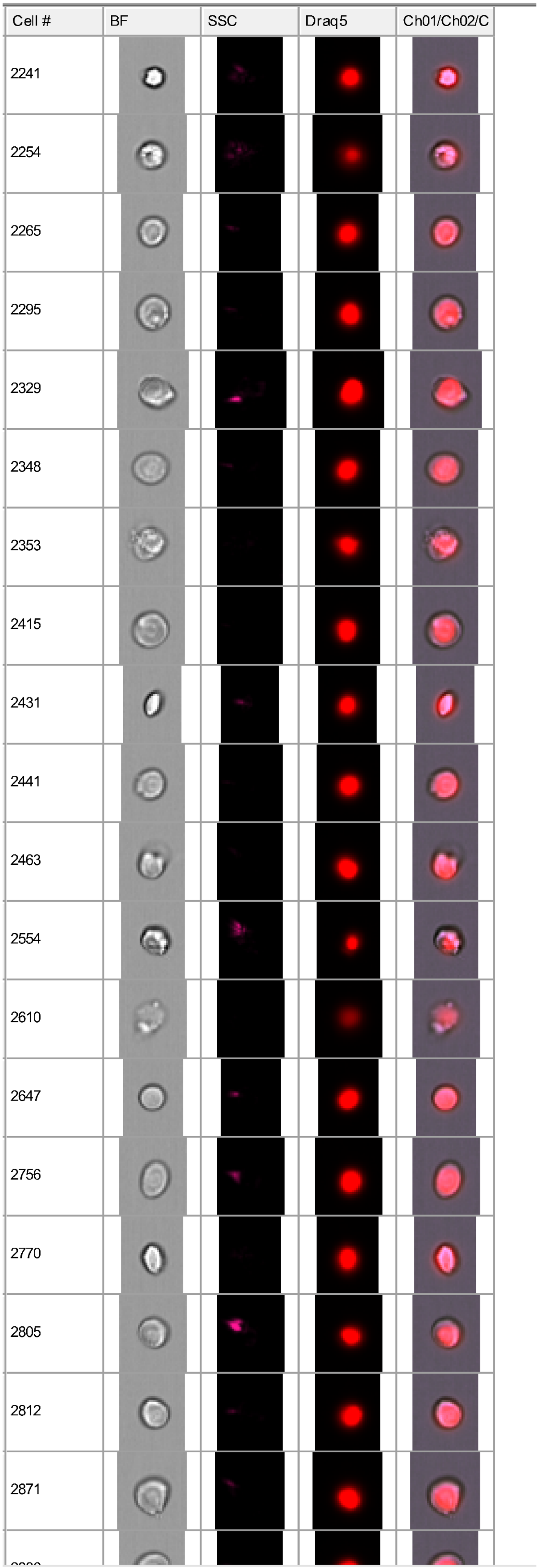

Cluster
*Dr*15

Granulocytic / Monocytic cells

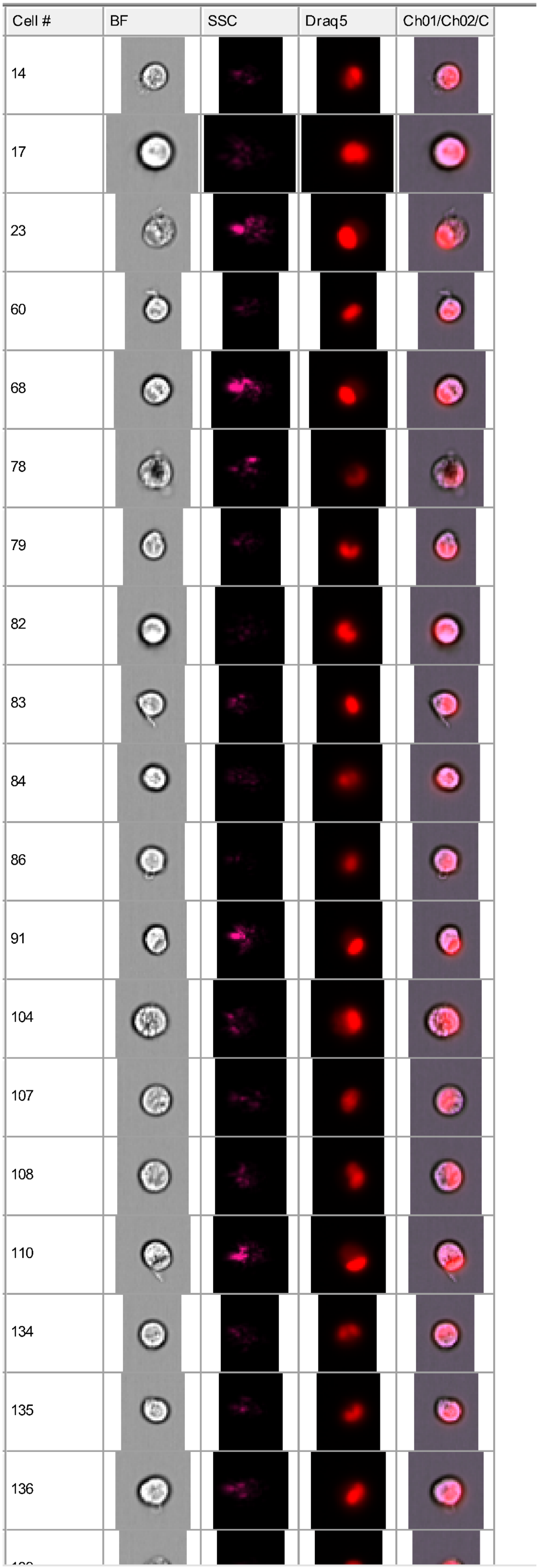

Cluster
*Dr*16

Erythrocytes

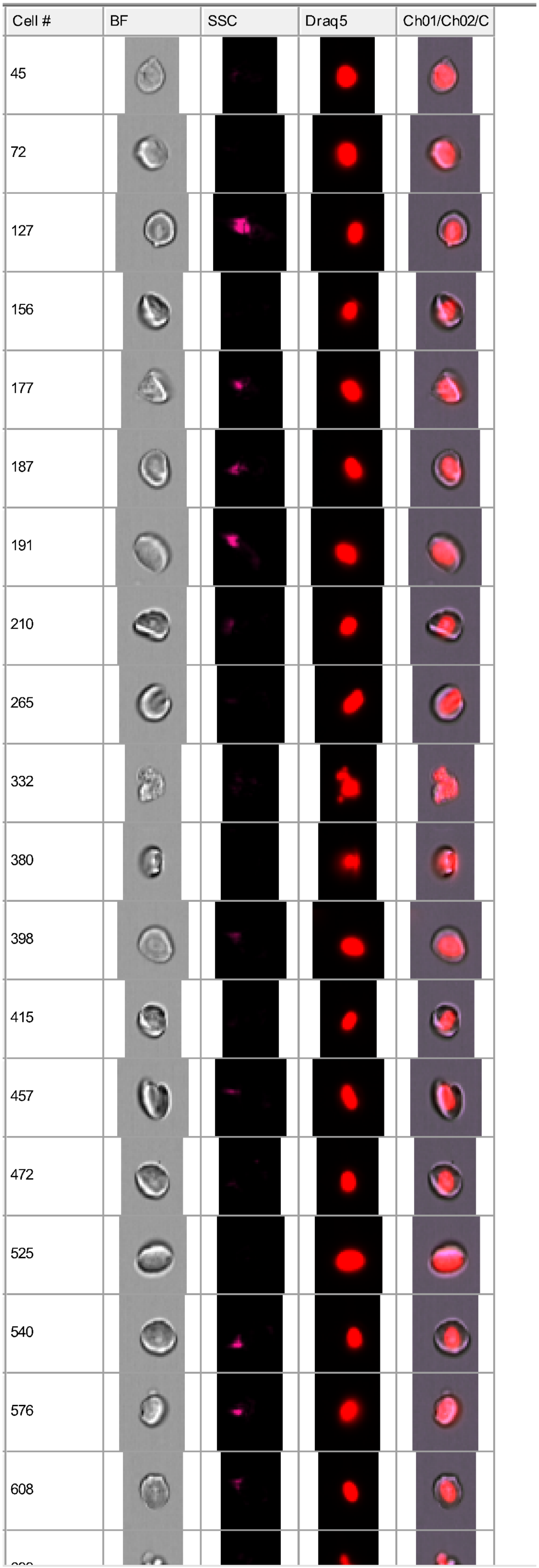

Supplement: Supplementary file 4. — Representative cell images belonging to each individual cluster identified by Image3C for zebrafish WKM in homeostasis condition are shown. BF: brightfield; SSC: side scatter signal; Draq5: nuclear staining. Merge represents the overlay of BF, SSC, and Draq5. [file elife-65372-supp4.pdf]
