# Supplementary material for: Image3C, a multimodal image-based and label-independent integrative method for single-cell analysis
Source: eLife. 2021 Jul 21;10:e65372. doi: 10.7554/eLife.65372 (PMC8370771; doi:10.7554/eLife.65372)
Supplement: Supplementary file 5. — Results of negative binomial regression analysis comparing cluster relative abundance between phagocytosis samples (CTV-S. aureus) and phagocytosis inhibited with CCB samples (CTV-S. aureus + CCB) in the zebrafish phagocytosis experiment. FC: fold change; CPM: count per million; LR: likelihood ratio; FDR: false discovery rate. Relative graph is reported in Figure 3B. [file elife-65372-supp5.docx]

**Supplementary File 5: Phagocytosis vs phagocytosis inhibited with CCB on zebrafish WKM**

Results of negative binomial regression analysis comparing cluster relative abundance between phagocytosis samples (CTV-S. aureus) vs phagocytosis inhibited with CCB samples (CTV-S. aureus + CCB) in the zebrafish phagocytosis experiment. FC is Fold Change, CPM is Count Per Million, LR is Likelihood Ratio, FDR is Fold Discovery Rate. Relative graph is reported in Figure 3B.

| **Cluster ID** | **logFC** | **logCPM** | **LR** | **PValue** | **FDR** |
| --- | --- | --- | --- | --- | --- |
| *Dr*1_P | -2.48673 | 14.76127 | 24.65067 | 6.9E-07 | 1.2E-06 |
| *Dr*2_P | -3.82090 | 15.11433 | 30.32912 | 3.7E-08 | 7.0E-08 |
| *Dr*3_P | -2.63248 | 15.10065 | 30.25504 | 3.8E-08 | 7.0E-08 |
| *Dr*5_P | -2.76060 | 14.21908 | 33.08750 | 8.8E-09 | 1.9E-08 |
| *Dr*6_P | -2.70177 | 13.37119 | 36.16033 | 1.8E-09 | 4.7E-09 |
| *Dr*7_P | -2.72126 | 14.24771 | 34.08437 | 5.3E-09 | 1.3E-08 |
| *Dr*8_P | 4.48271 | 14.88169 | 82.05466 | 1.3E-19 | 4.9E-19 |
| *Dr*10_P | -3.45902 | 14.60211 | 24.35992 | 8.0E-07 | 1.3E-06 |
| *Dr*11_P | 6.90476 | 13.91280 | 84.08534 | 4.7E-20 | 2.1E-19 |
| *Dr*12_P | 1.08728 | 12.83107 | 11.29997 | 7.8E-04 | 1.1E-03 |
| *Dr*13_P | 1.51443 | 12.94985 | 11.48213 | 7.0E-04 | 1.0E-03 |
| *Dr*14_P | -2.99602 | 11.50543 | 42.88105 | 5.8E-11 | 1.7E-10 |
| *Dr*15_P | -2.38678 | 12.70026 | 21.12804 | 4.3E-06 | 6.6E-06 |
| *Dr*16_P | 5.66338 | 14.13744 | 143.18631 | 5.4E-33 | 1.4E-31 |
| *Dr*17_P | 5.71512 | 14.80998 | 122.27043 | 2.0E-28 | 2.6E-27 |
| *Dr*19_P | 4.07753 | 14.85917 | 93.86068 | 3.4E-22 | 1.8E-21 |
| *Dr*20_P | 3.84792 | 13.05544 | 49.27037 | 2.2E-12 | 7.3E-12 |
| *Dr*21_P | 1.57131 | 11.87021 | 9.42118 | 2.1E-03 | 2.8E-03 |
| *Dr*23_P | 4.37593 | 16.67274 | 99.99839 | 1.5E-23 | 9.9E-23 |
| *Dr*25_P | 5.76977 | 13.99753 | 119.32182 | 8.9E-28 | 7.7E-27 |
