# Supplementary material for: Image3C, a multimodal image-based and label-independent integrative method for single-cell analysis
Source: eLife. 2021 Jul 21;10:e65372. doi: 10.7554/eLife.65372 (PMC8370771; doi:10.7554/eLife.65372)
Supplement: Supplementary file 6. — Results of negative binomial regression analysis comparing cluster relative abundance between phagocytosis samples (CTV-S. aureus) and phagocytosis inhibited with ice samples (CTV-S. aureus + Ice) in the zebrafish phagocytosis experiment. FC: fold change; CPM: count per million; LR: likelihood ratio; FDR: false discovery rate. Relative graph is reported in Figure 3—figure supplement 2. [file elife-65372-supp6.docx]

**Supplementary File 6: Phagocytosis vs phagocytosis inhibited with ice on zebrafish WKM**

Results of negative binomial regression analysis comparing cluster relative abundance between phagocytosis samples (CTV-S. aureus) vs phagocytosis inhibited with ice samples (CTV-S. aureus + Ice) in the zebrafish phagocytosis experiment. FC is Fold Change, CPM is Count Per Million, LR is Likelihood Ratio, FDR is Fold Discovery Rate. Relative graph is reported in Figure 3-figure supplement 2.

| **Cluster ID** | **logFC** | **logCPM** | **LR** | **PValue** | **FDR** |
| --- | --- | --- | --- | --- | --- |
| *Dr*1_P | -2.57222 | 14.76127 | 26.21074 | 3.1E-07 | 2.7E-06 |
| *Dr*3_P | -1.47929 | 15.10065 | 10.27905 | 1.3E-03 | 5.0E-03 |
| *Dr*5_P | 1.23557 | 14.21908 | 6.95840 | 8.3E-03 | 2.4E-02 |
| *Dr*6_P | -1.96681 | 13.37119 | 19.93869 | 8.0E-06 | 5.2E-05 |
| *Dr*7_P | -1.93868 | 14.24771 | 18.19453 | 2.0E-05 | 1.0E-04 |
| *Dr*9_P | 4.34094 | 11.68675 | 36.21393 | 1.8E-09 | 4.6E-08 |
| *Dr*12_P | 0.83638 | 12.83107 | 6.79951 | 9.1E-03 | 2.4E-02 |
| *Dr*14_P | -2.35912 | 11.50543 | 26.47869 | 2.7E-07 | 2.7E-06 |
| *Dr*15_P | -1.67916 | 12.70026 | 10.80552 | 1.0E-03 | 4.4E-03 |
| *Dr*24_P | 1.08190 | 16.03192 | 8.34069 | 3.9E-03 | 1.3E-02 |
