# Supplementary material for: Image3C, a multimodal image-based and label-independent integrative method for single-cell analysis
Source: eLife. 2021 Jul 21;10:e65372. doi: 10.7554/eLife.65372 (PMC8370771; doi:10.7554/eLife.65372)
Supplement: Supplementary file 7. — Representative cell images belonging to each individual cluster identified by Image3C for zebrafish WKM after phagocytosis assay are shown. BF: brightfield; DHR: fluorescent reactive oxygen species indicator; SSC: side scatter signal; Bac: CTV signal (S. aureus labeling); Draq5: nuclear staining. Merge represents the overlay of DHR, Bac, and Draq5. [file elife-65372-supp7.pdf]

Zebrafish Phagocytosis Experiment

Cell Gallery

*Dr*1_P

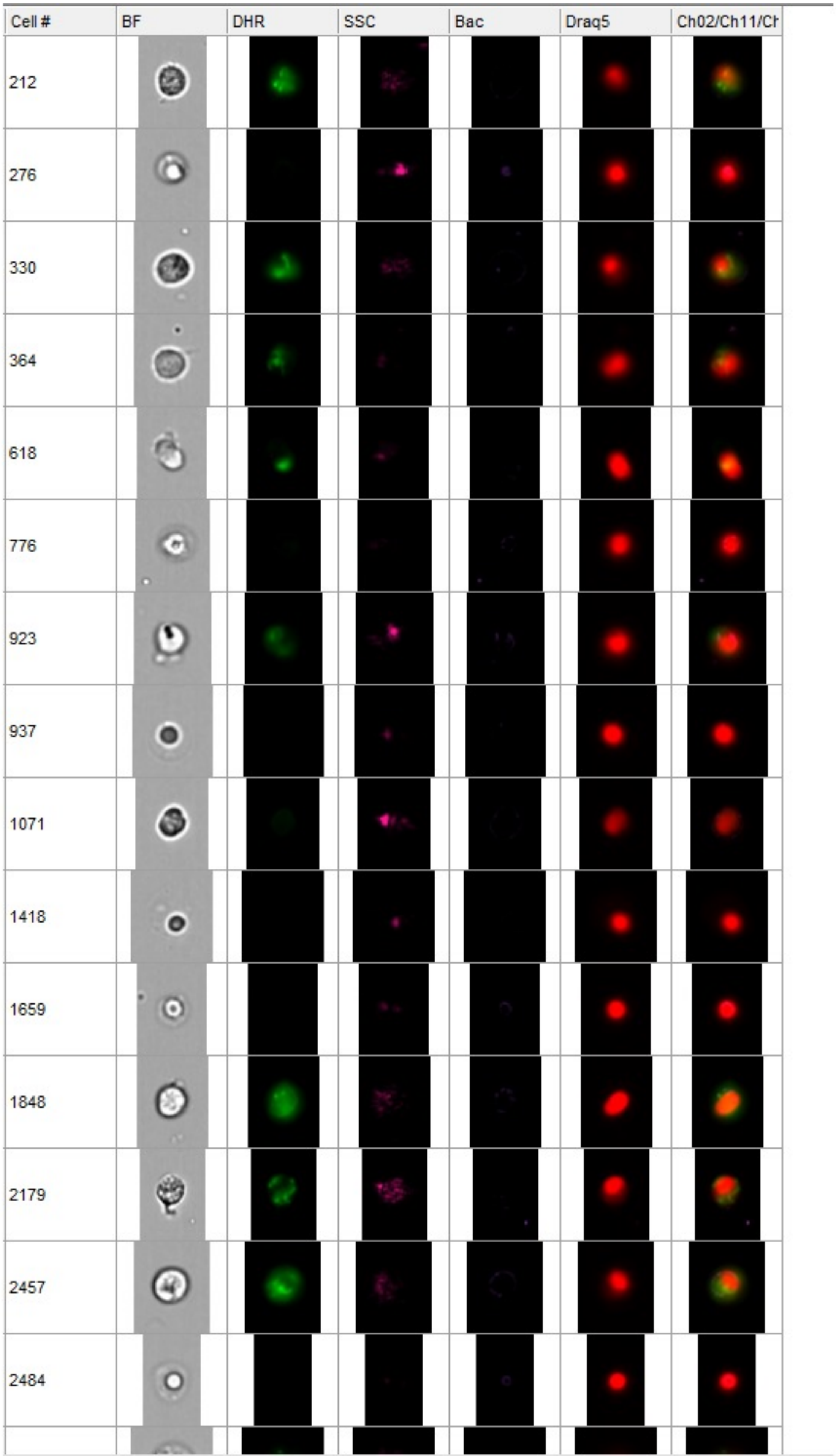
Cell #
BF
DHR
SSC
Bac
Draq5
Ch02/Ch11/Ch
212
276
330
364
618
776
923
937
1071
1418
1659
1848
2179
2457
2484

*Dr*2_P

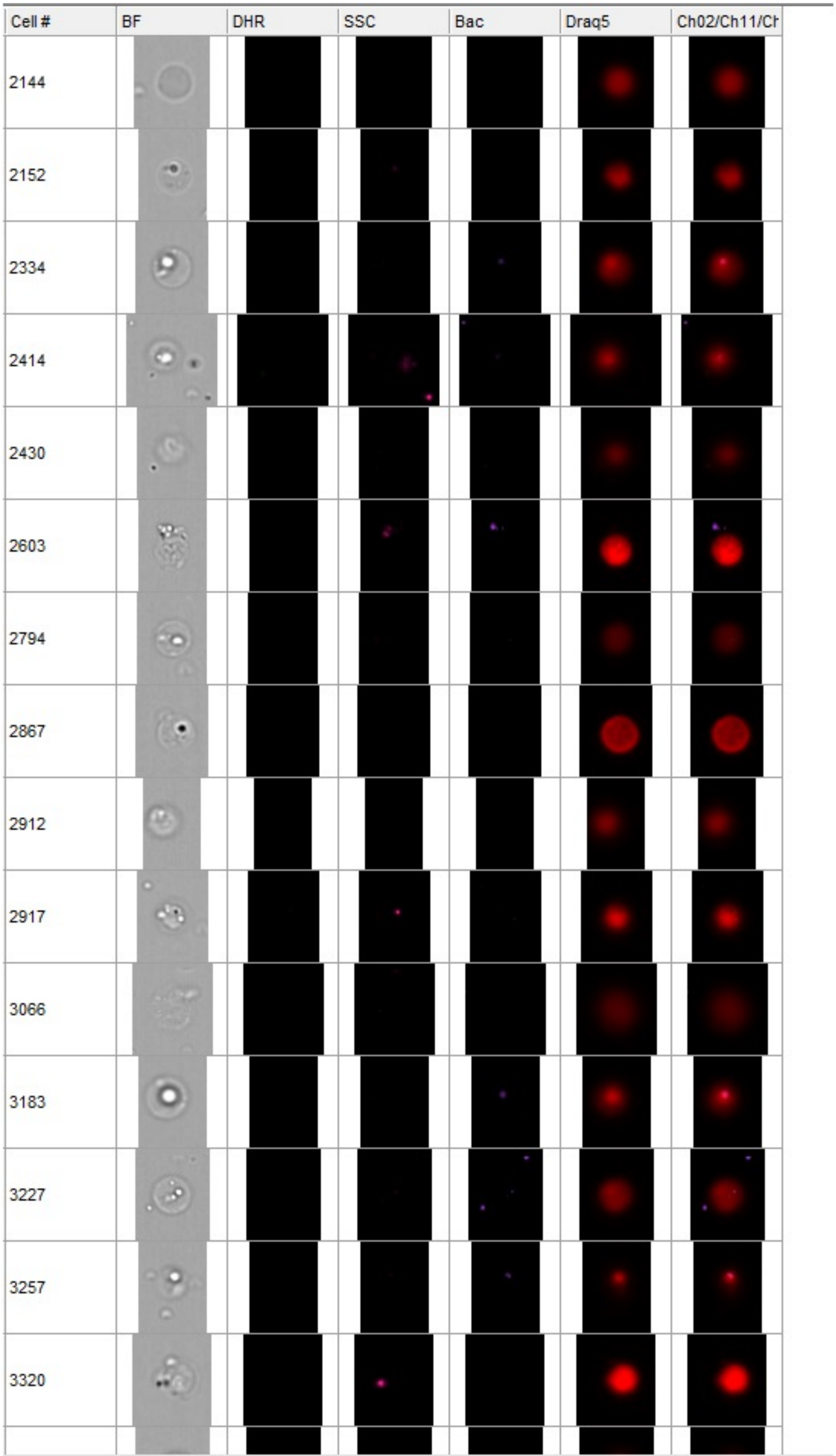
Cell #
BF
DHR
SSC
Bac
Draq5
Ch02/Ch11/Ch
2144
2152
2334
2414
2430
2603
2794
2867
2912
2917
3066
3183
3227
3257
3320

*Dr*3_P

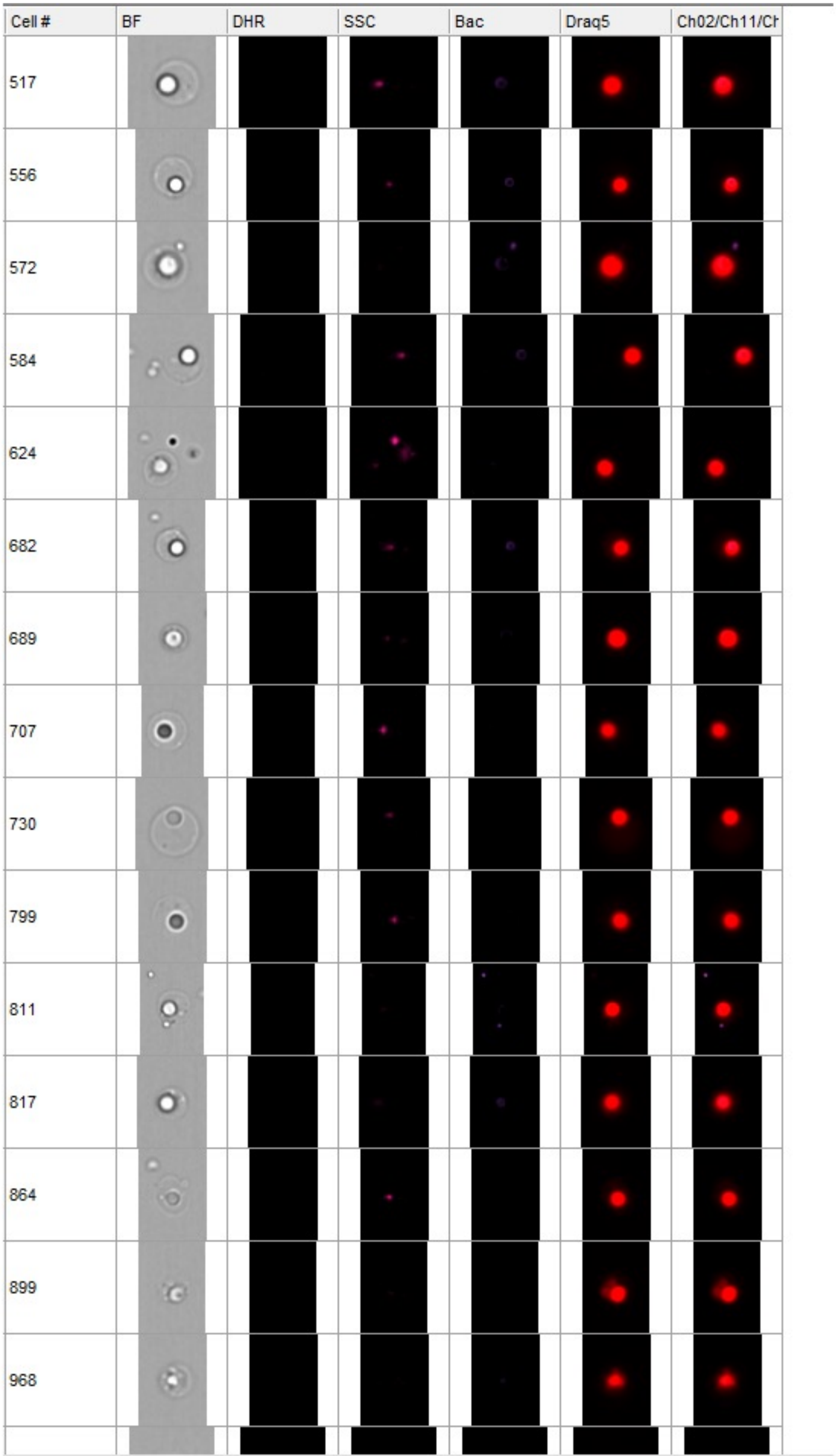

Cell #
BF
DHR
SSC
Bac
Draq5
Ch02/Ch11/Ch
517
556
572
584
624
682
689
707
730
799
811
817
864
899
968

*Dr*4_P   Professional phagocytes

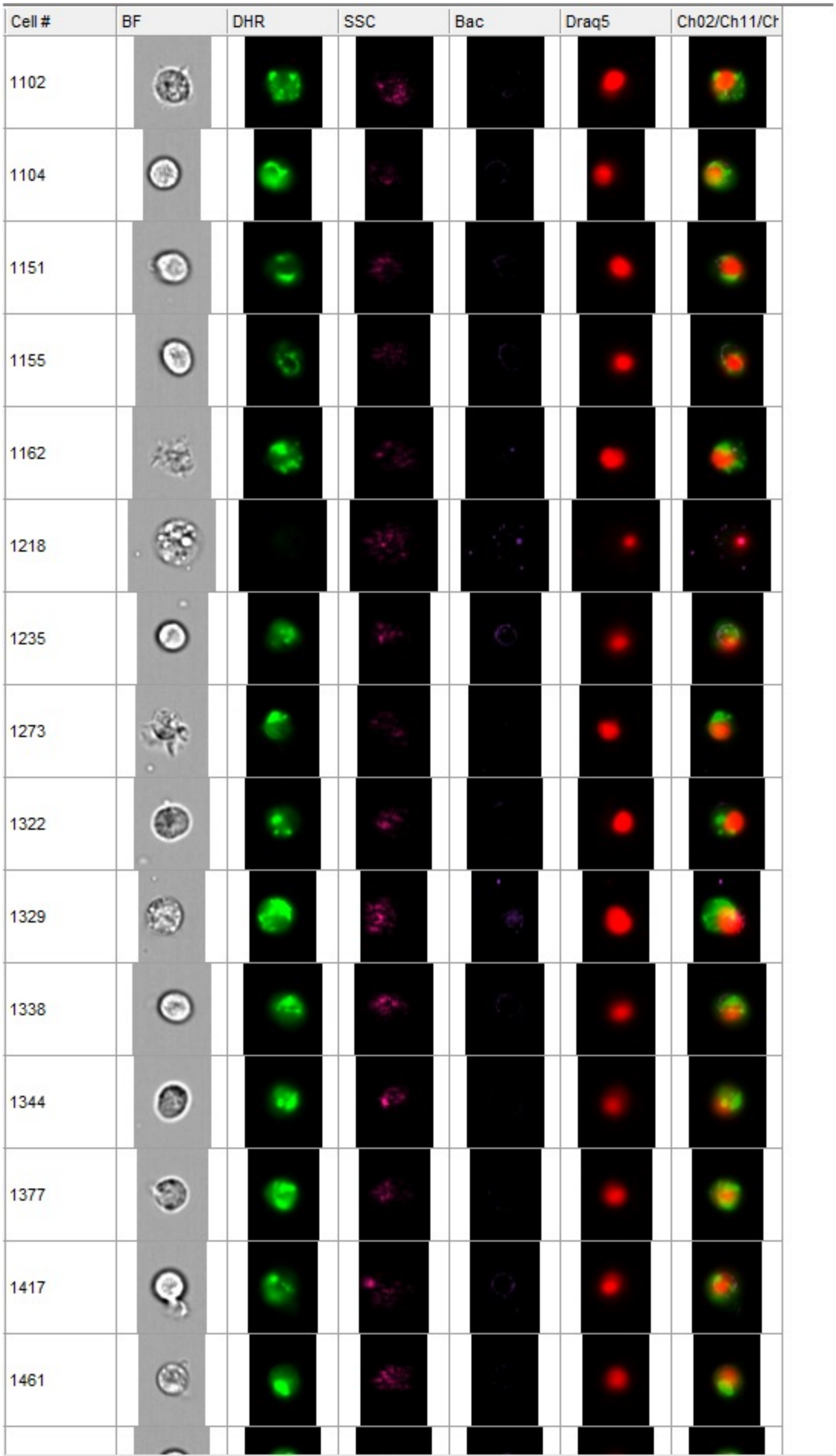

*Dr*5_P

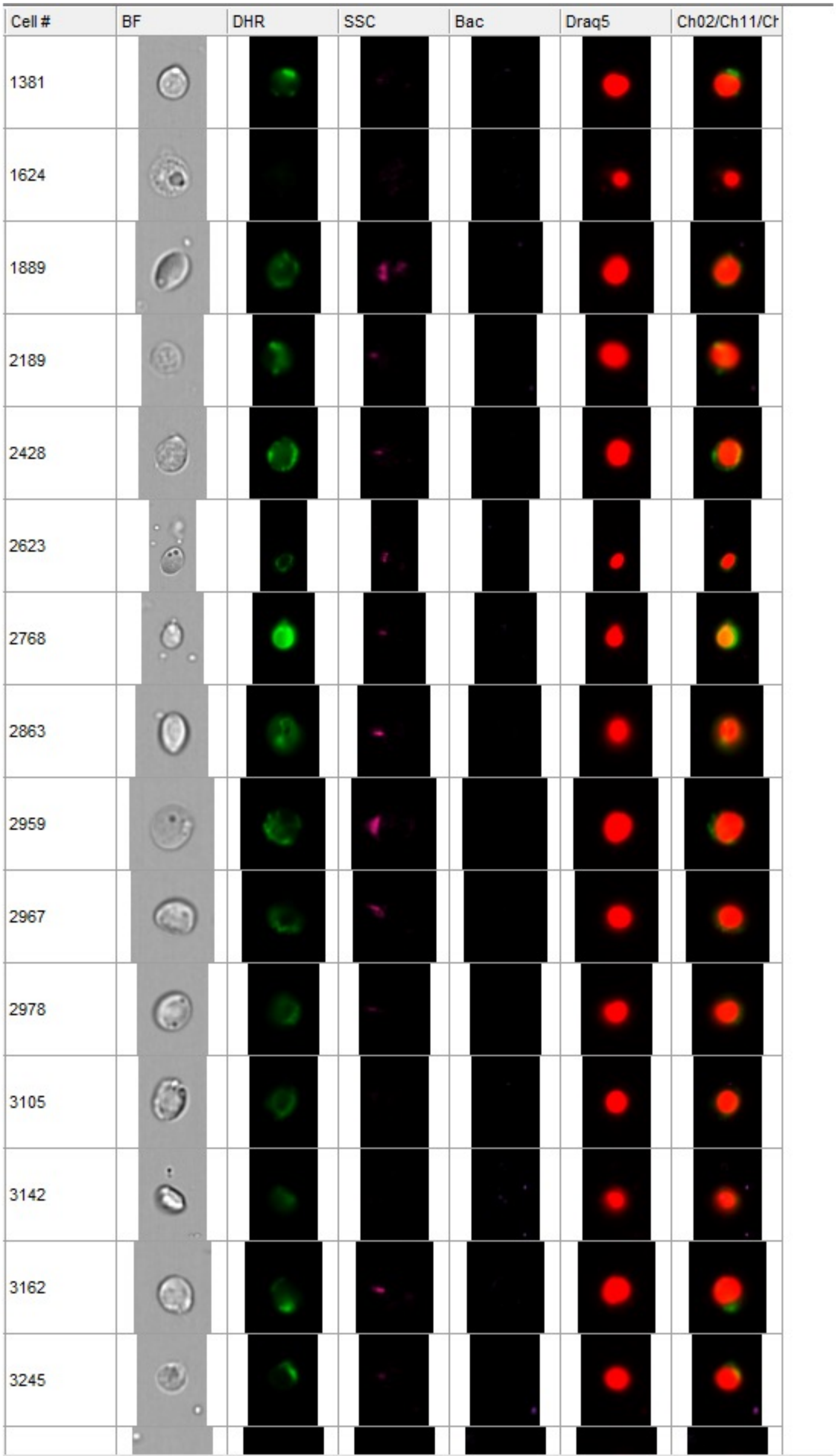
Cell #
BF
DHR
SSC
Bac
Draq5
Ch02/Ch11/Ch
1381
1624
1889
2189
2428
2623
2768
2863
2959
2967
2978
3105
3142
3162
3245

*Dr*6_P

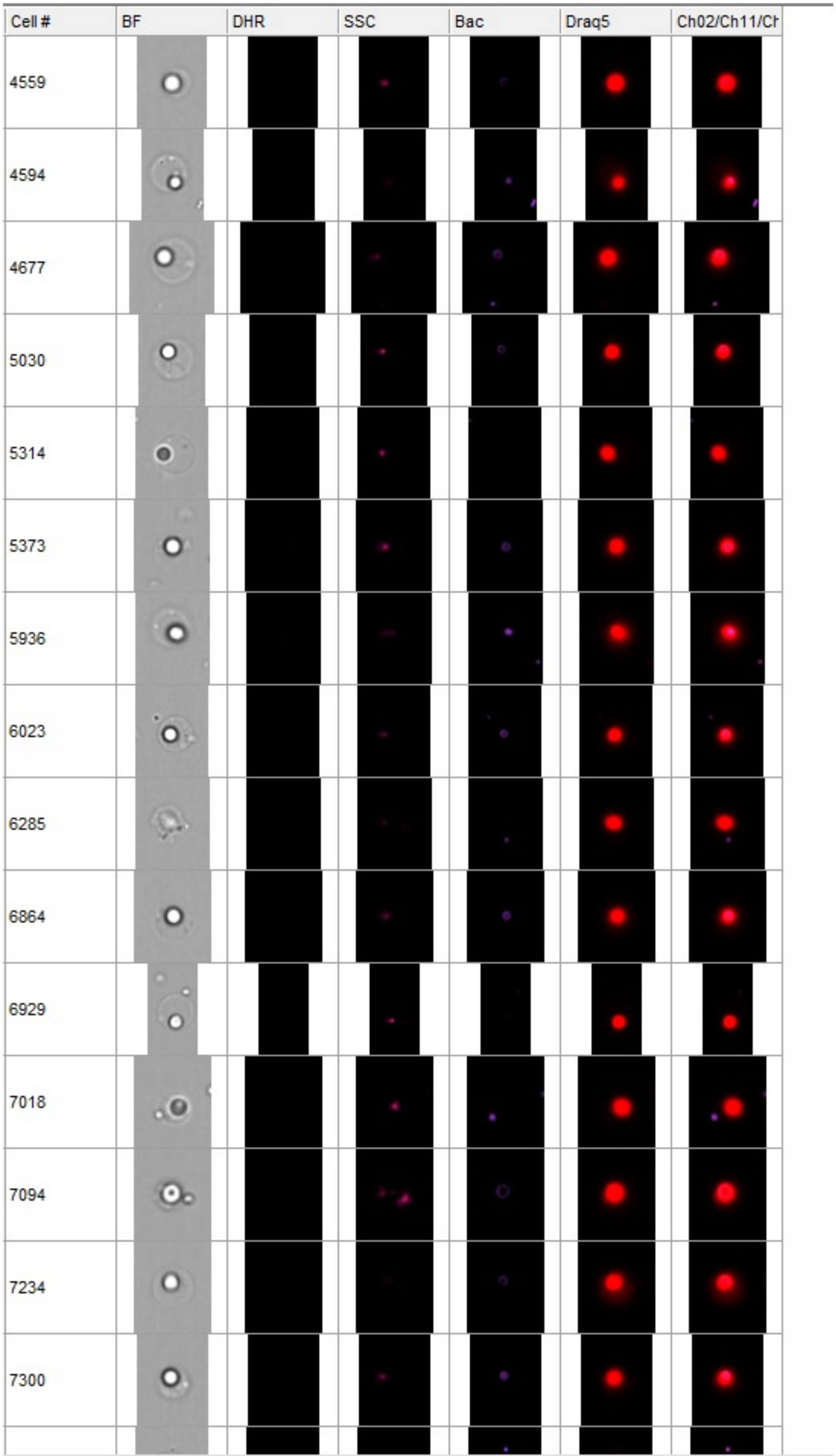
Cell #
BF
DHR
SSC
Bac
Draq5
Ch02/Ch11/Ch
4559
4594
4677
5030
5314
5373
5936
6023
6285
6864
6929
7018
7094
7234
7300

*Dr7*_P

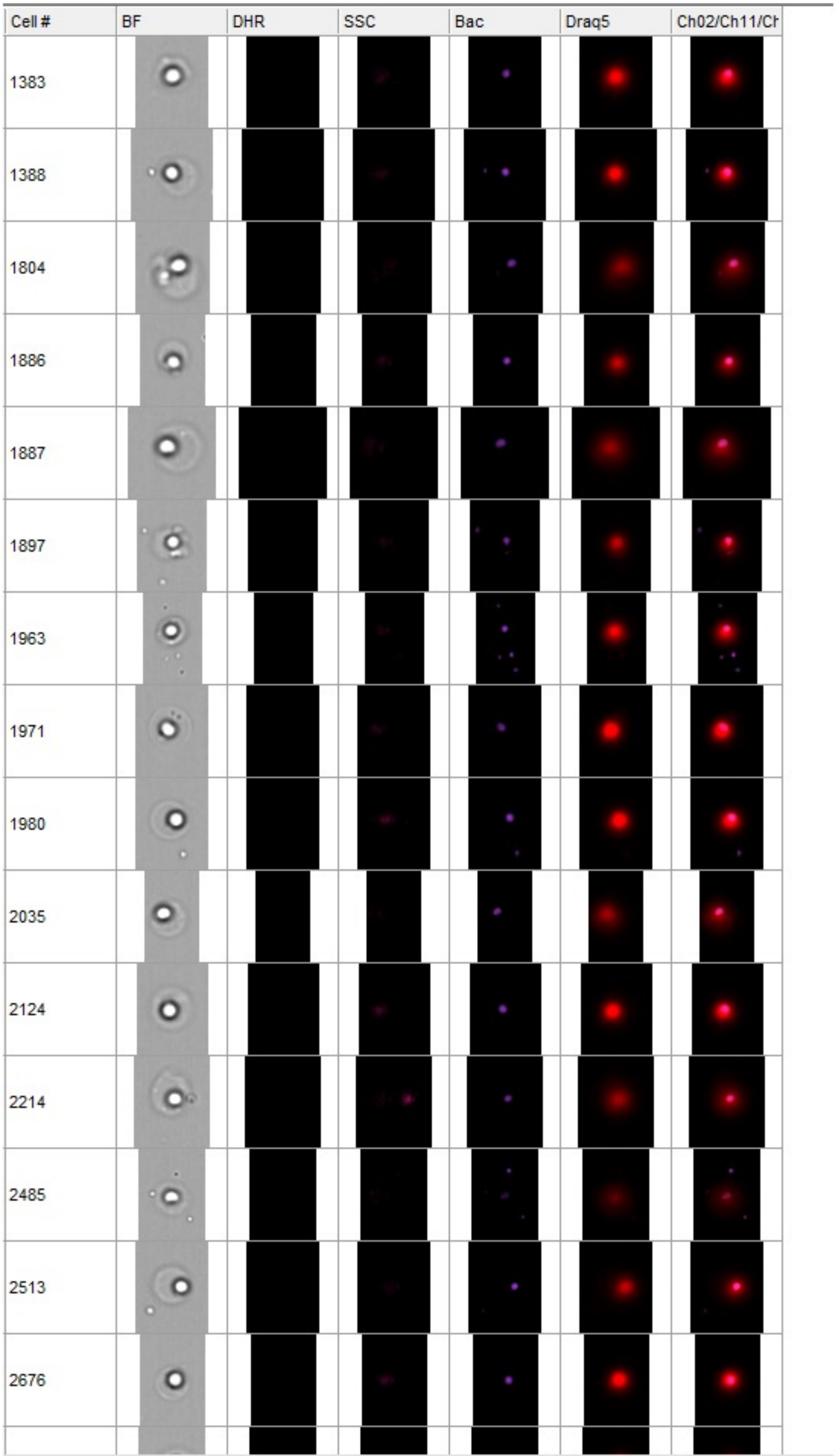
Cell #
BF
DHR
SSC
Bac
Draq5
Ch02/Ch11/Ch
1383
1388
1804
1886
1887
1897
1963
1971
1980
2035
2124
2214
2485
2513
2676

*Dr*8_P

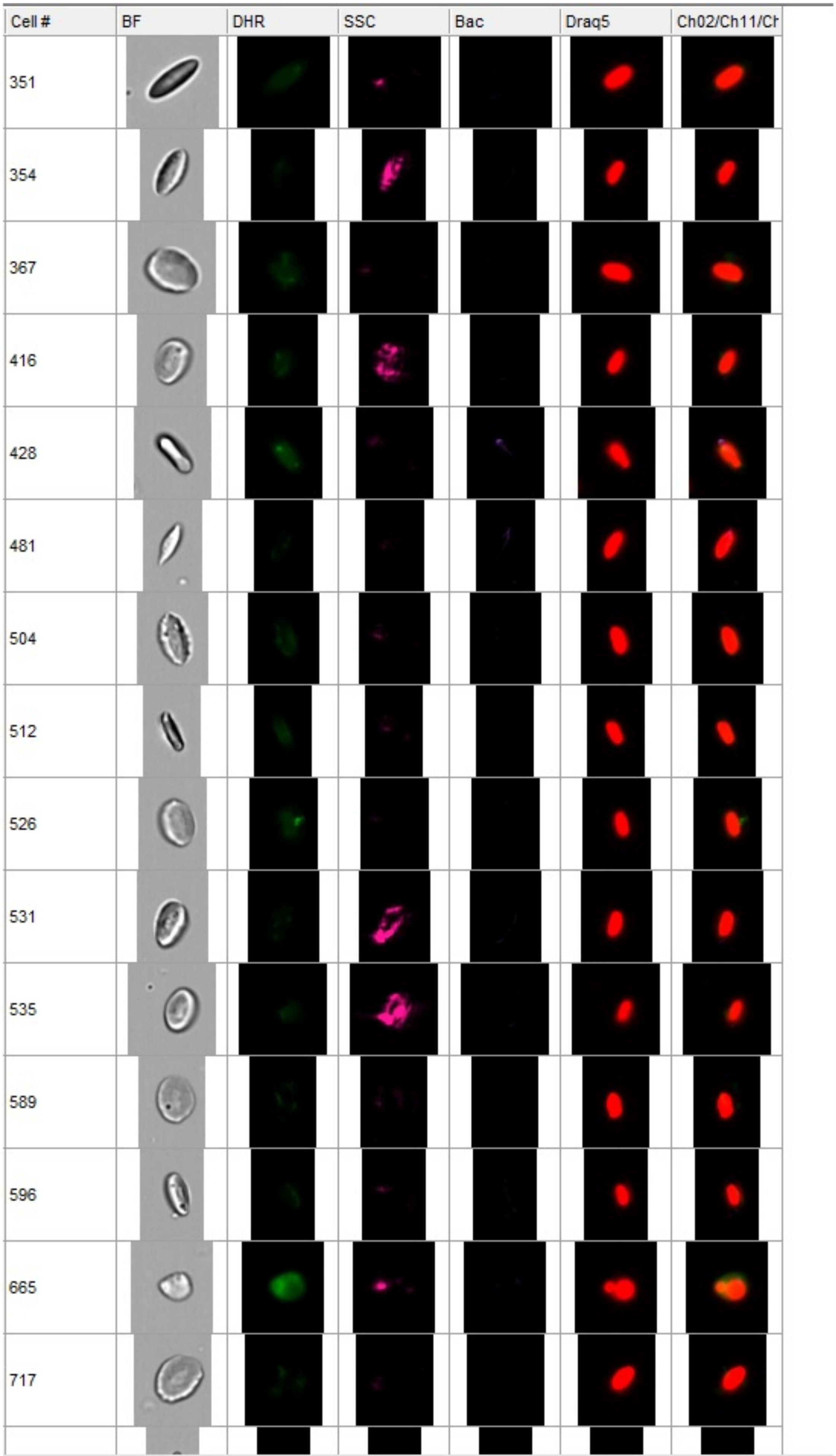
Cell #
BF
DHR
SSC
Bac
Draq5
Ch02/Ch11/Ch
351
354
367
416
428
481
504
512
526
531
535
589
596
665
717

*Dr*9_P

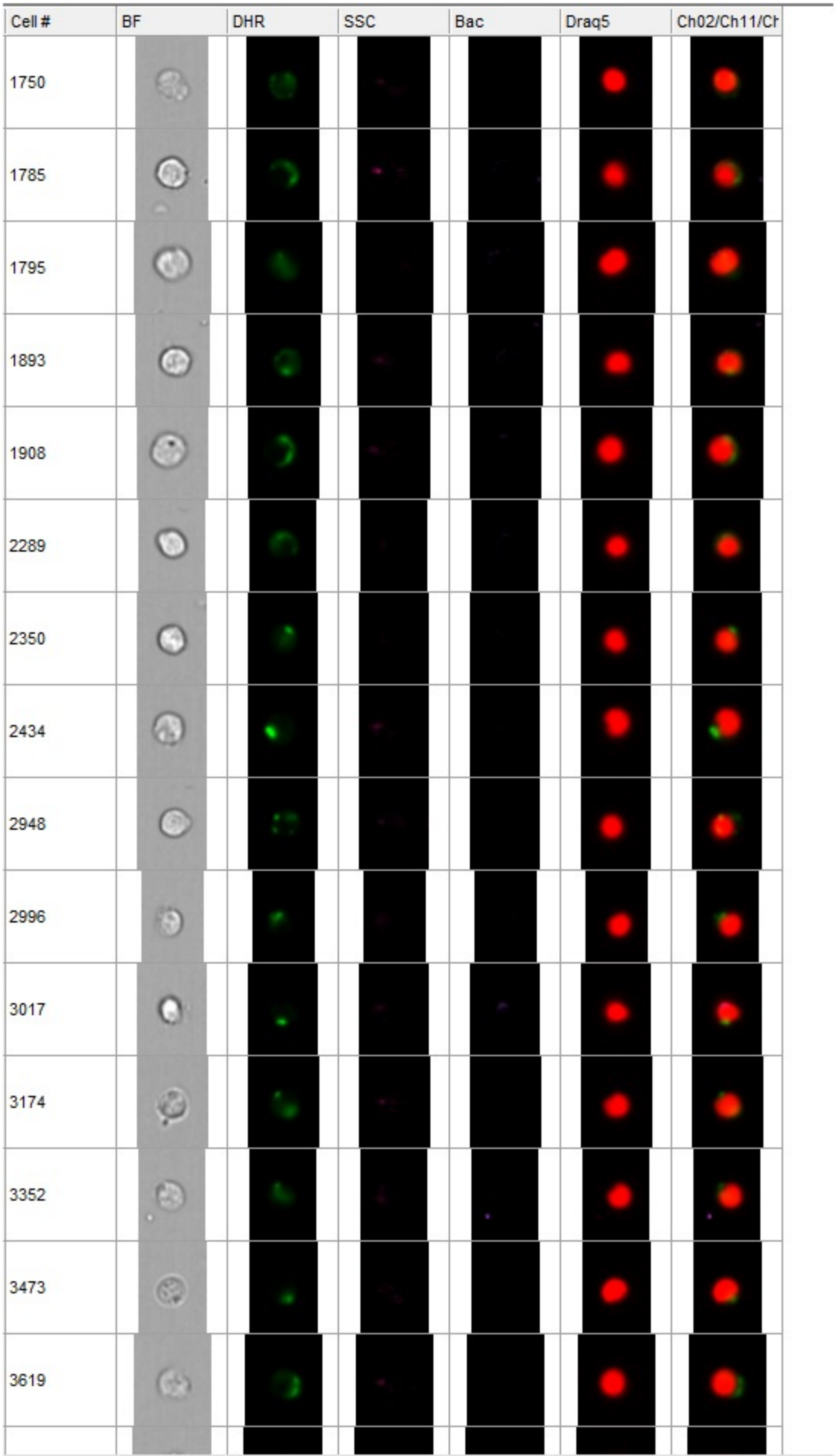

Cell #
BF
DHR
SSC
Bac
Draq5
Ch02/Ch11/Ch
1750
1785
1795
1893
1908
2289
2350
2434
2948
2996
3017
3174
3352
3473
3619

*Dr*10_P

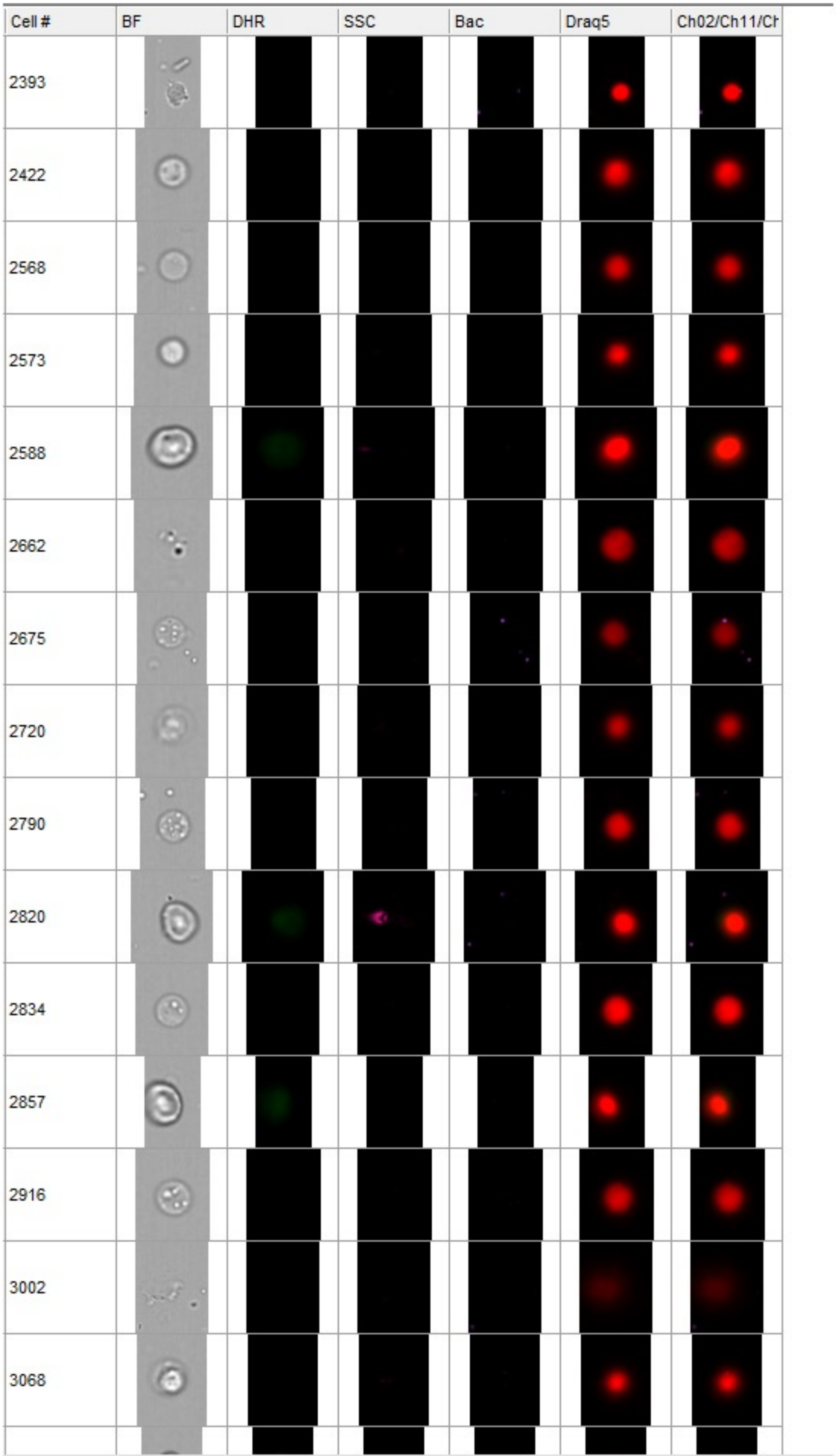
Cell #
BF
DHR
SSC
Bac
Draq5
Ch02/Ch11/Ch
2393
2422
2568
2573
2588
2662
2675
2720
2790
2820
2834
2857
2916
3002
3068

*Dr*11_P

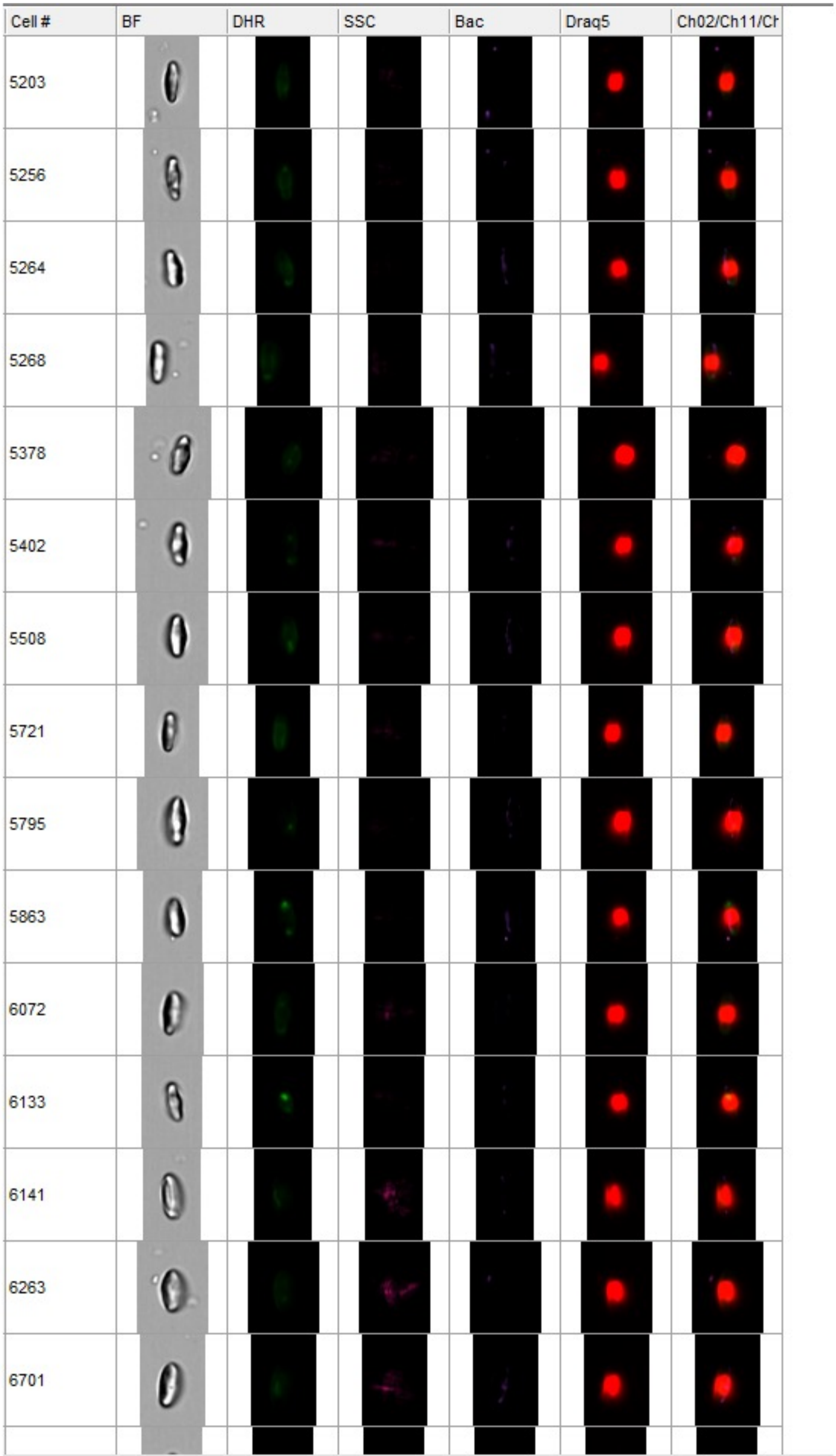

Cell #
BF
DHR
SSC
Bac
Draq5
Ch02/Ch11/Ch
5203
5256
5264
5268
5378
5402
5508
5721
5795
5863
6072
6133
6141
6263
6701

*Dr*12_P Professional phagocytes

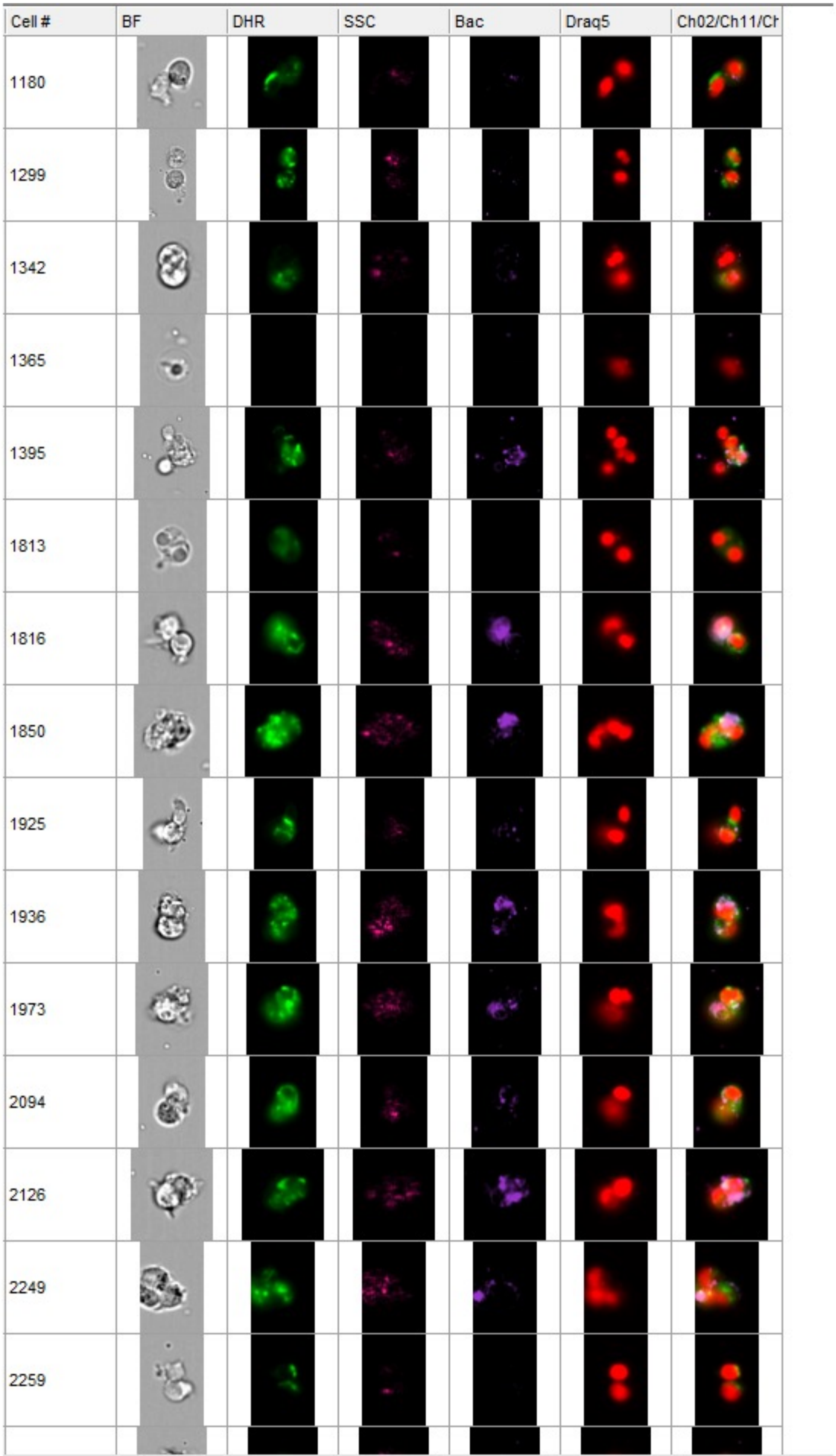

*Dr*13_P   Professional phagocytes

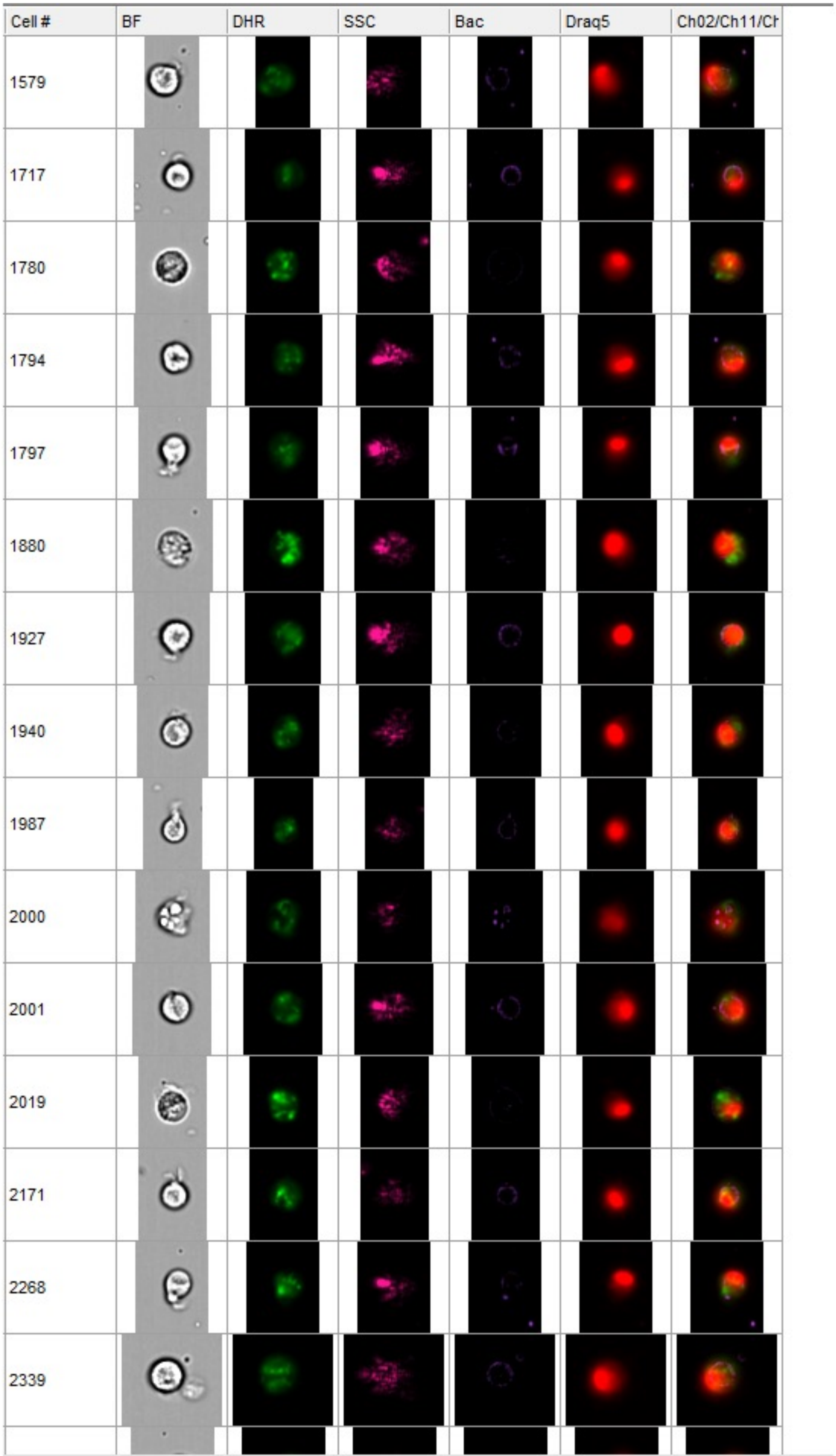

*Dr*14_P

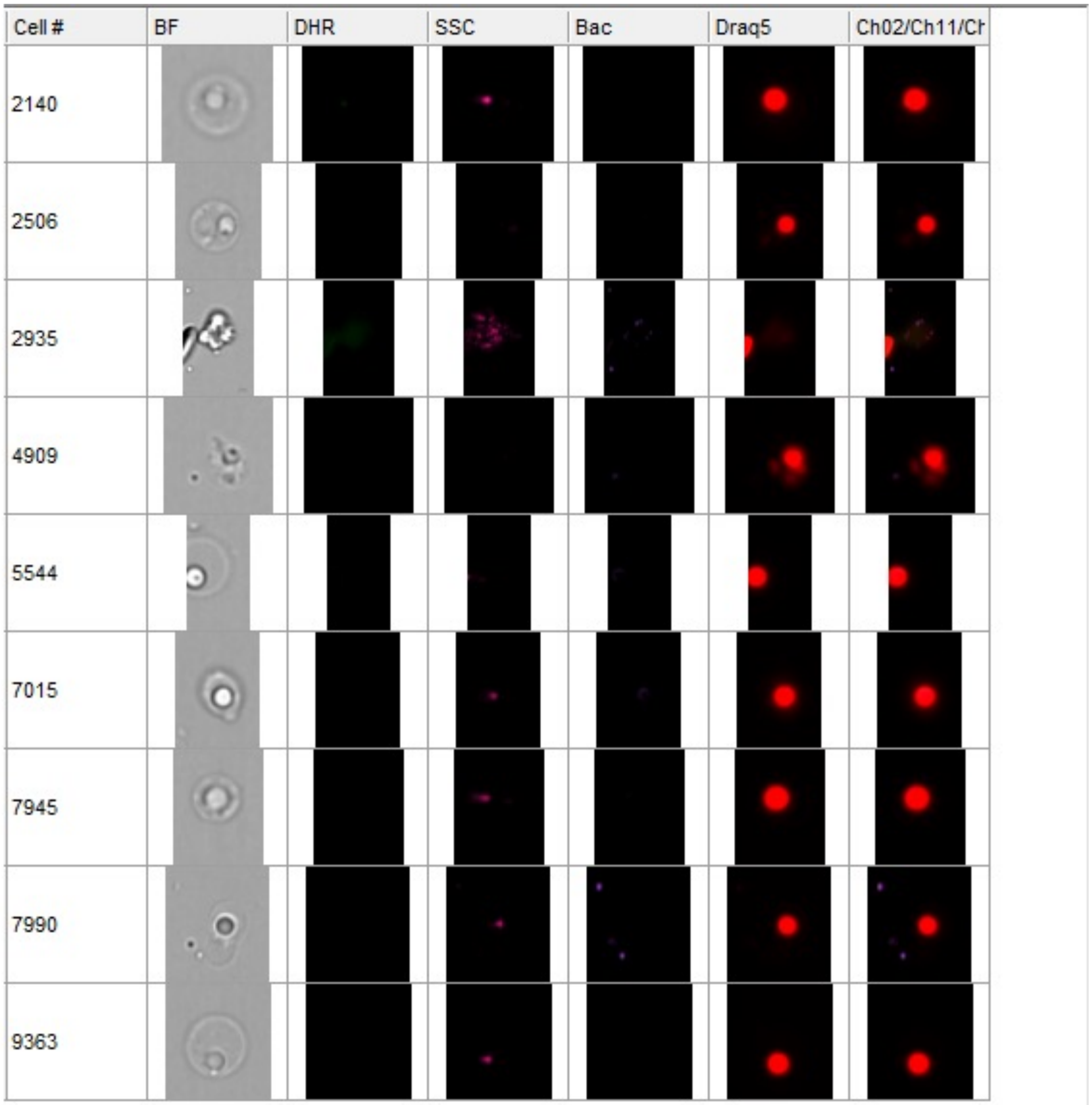
Cell #
BF
DHR
SSC
Bac
Draq5
Ch02/Ch11/Ch
2140
2506
2935
4909
5544
7015
7945
7990
9363

*Dr*15_P

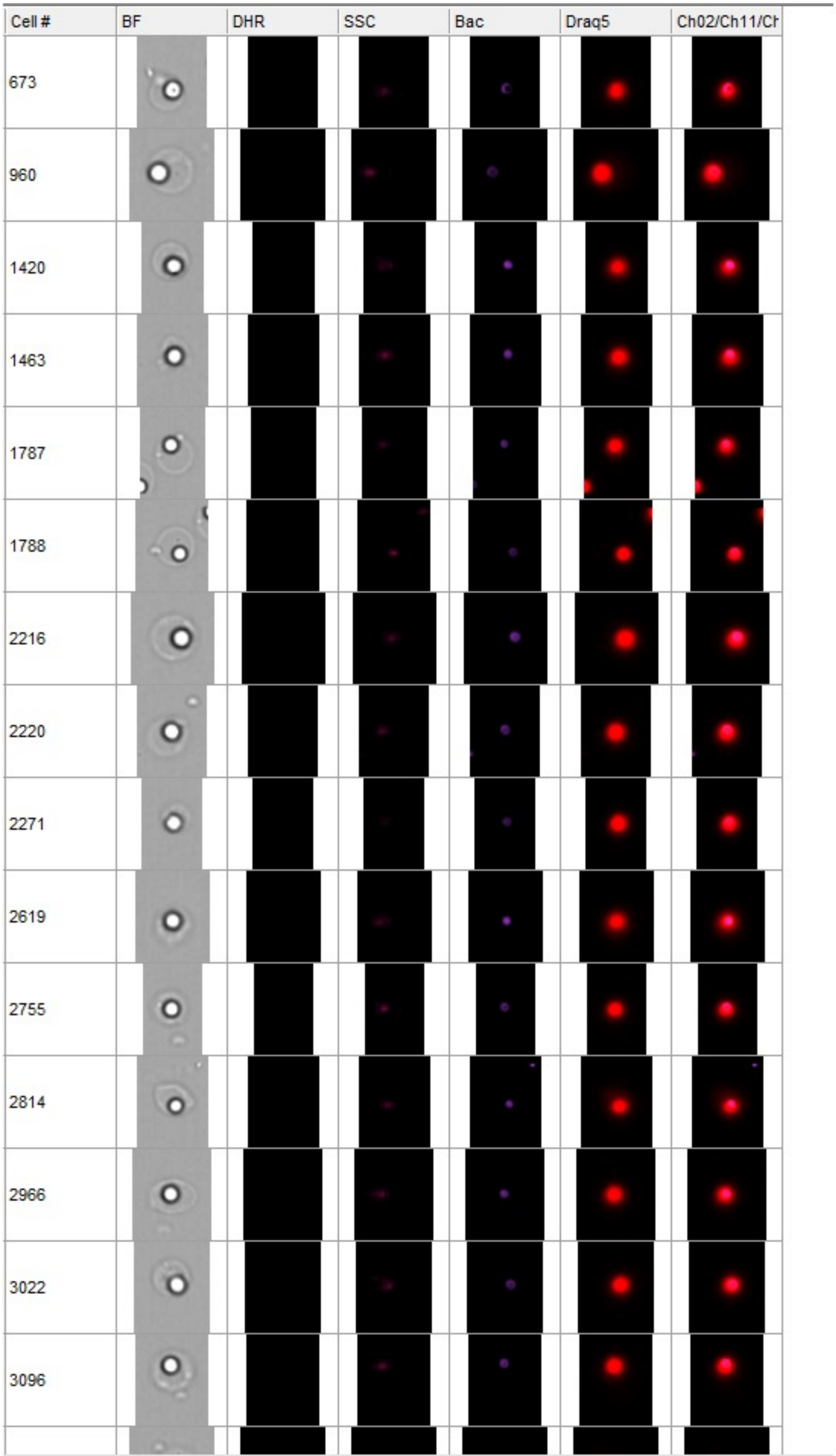
Cell #
BF
DHR
SSC
Bac
Draq5
Ch02/Ch11/Ch
673
960
1420
1463
1787
1788
2216
2220
2271
2619
2755
2814
2966
3022
3096

*Dr*16_P

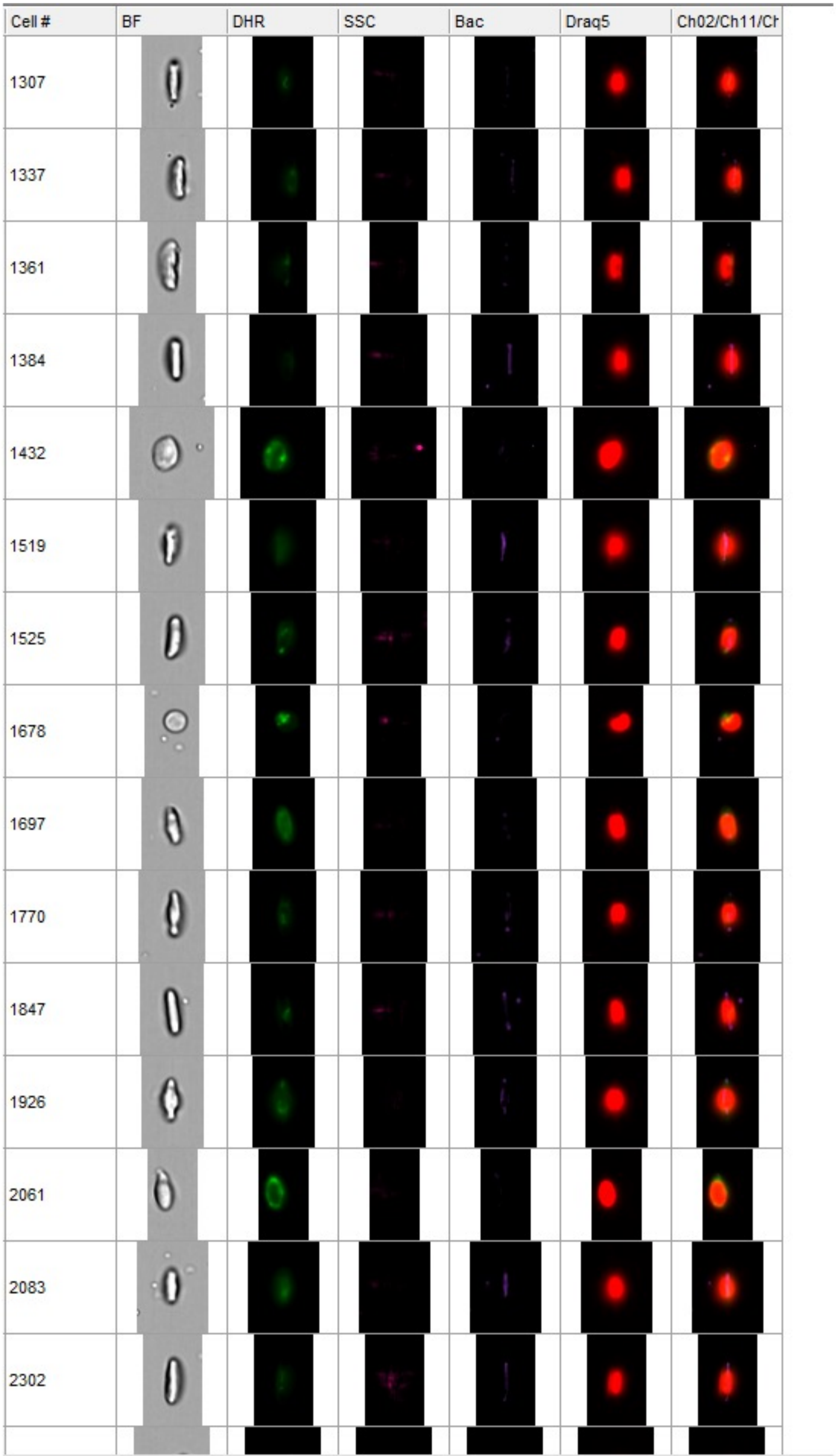

Cell #
BF
DHR
SSC
Bac
Draq5
Ch02/Ch11/Ch
1307
1337
1361
1384
1432
1519
1525
1678
1697
1770
1847
1926
2061
2083
2302

*Dr*17_P

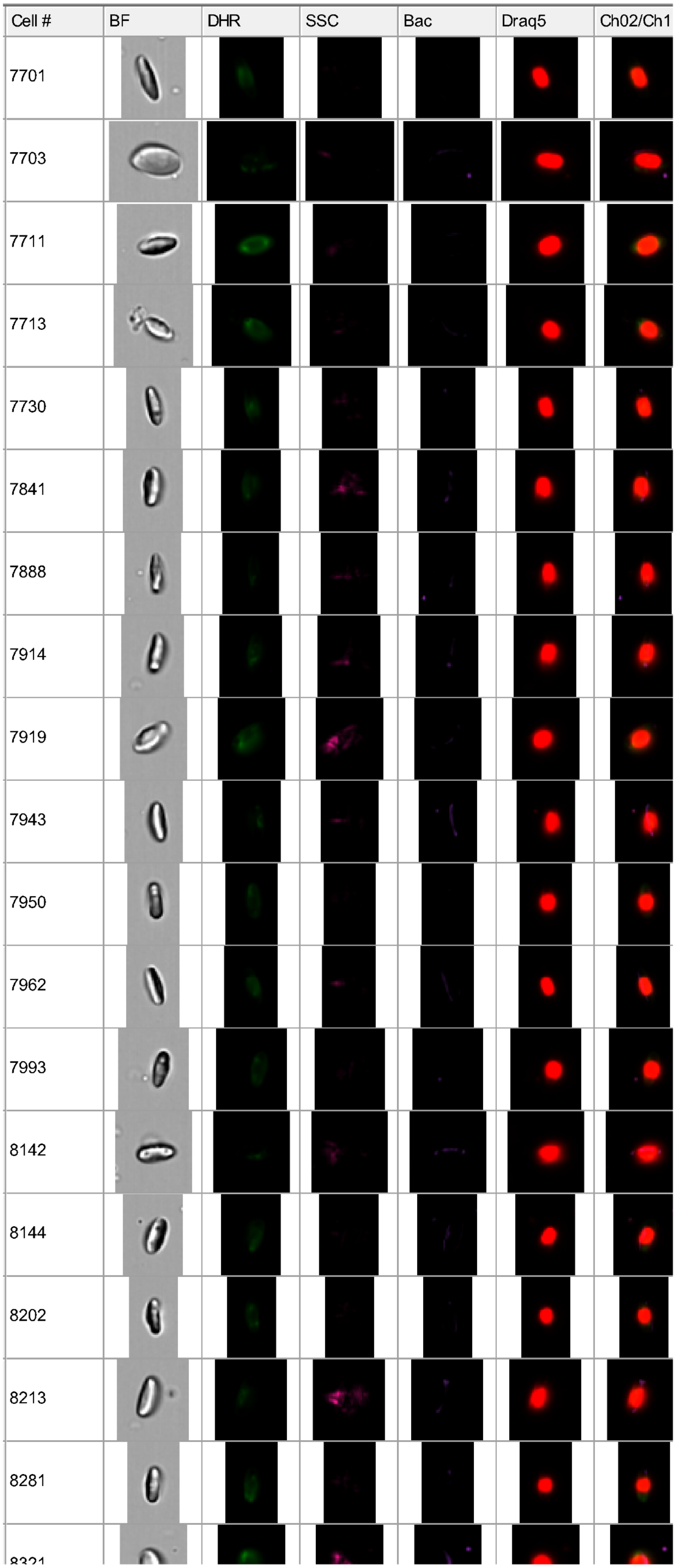
Cell #
BF
DHR
SSC
Bac
Draq5
Ch02/Ch1
7701
7703
7711
7713
7730
7841
7888
7914
7919
7943
7950
7962
7993
8142
8144
8202
8213
8281

*Dr*18_P

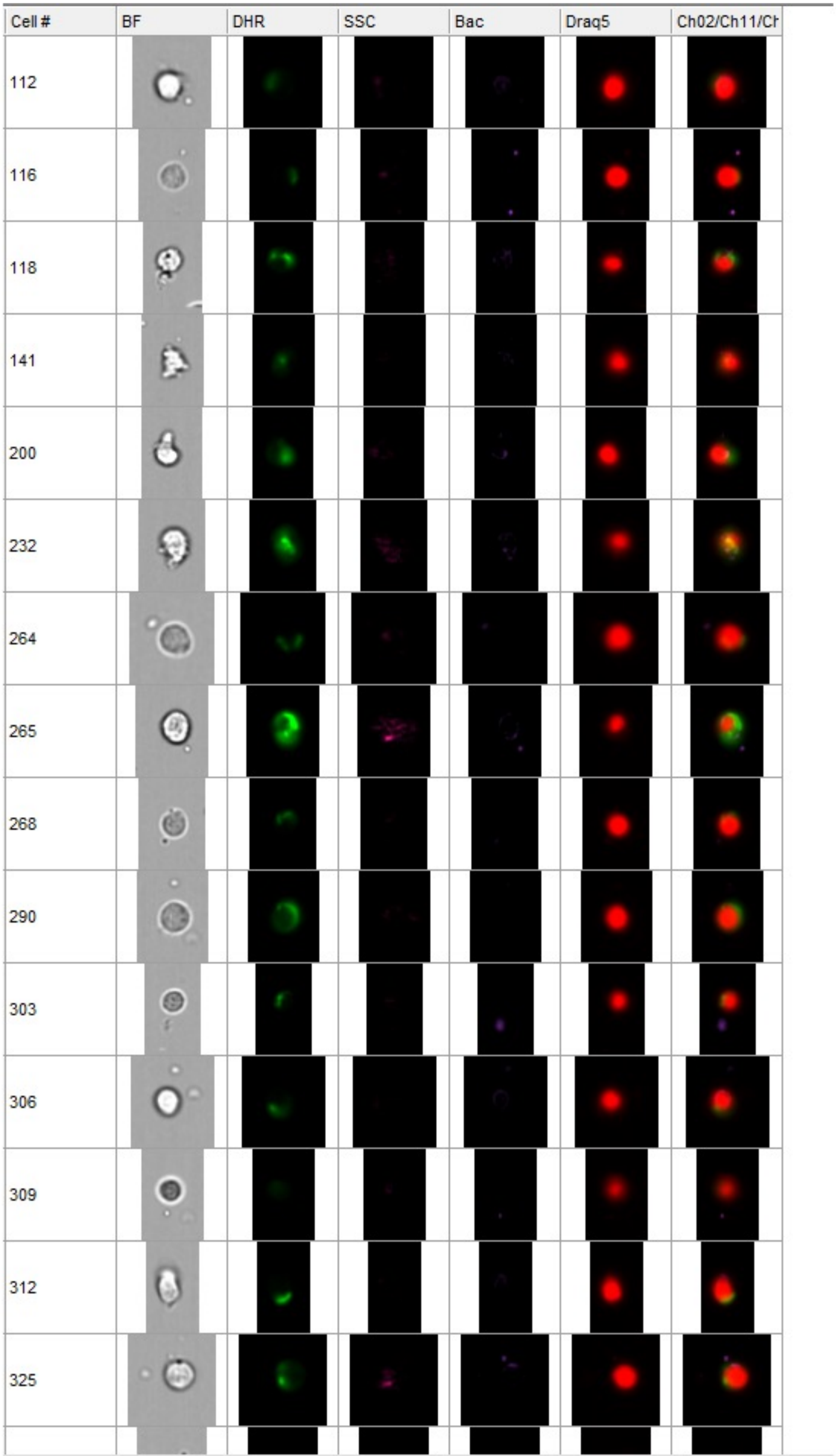
Cell #
BF
DHR
SSC
Bac
Draq5
Ch02/Ch11/Ch
112
116
118
141
200
232
264
265
268
290
303
306
309
312
325

*Dr*19_P

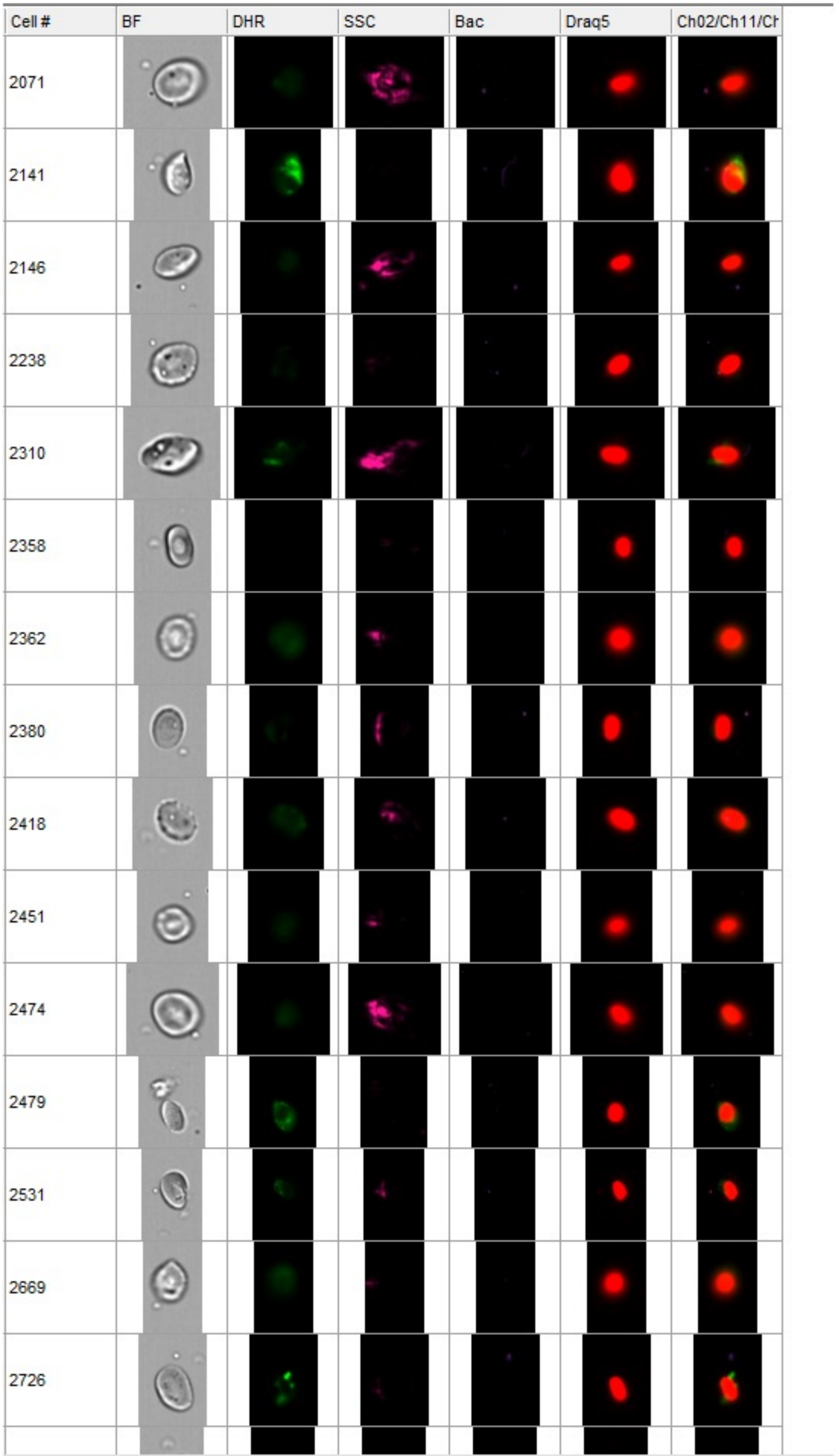
Cell #
BF
DHR
SSC
Bac
Draq5
Ch02/Ch11/Ch
2071
2141
2146
2238
2310
2358
2362
2380
2418
2451
2474
2479
2531
2669
2726

*Dr*20_P

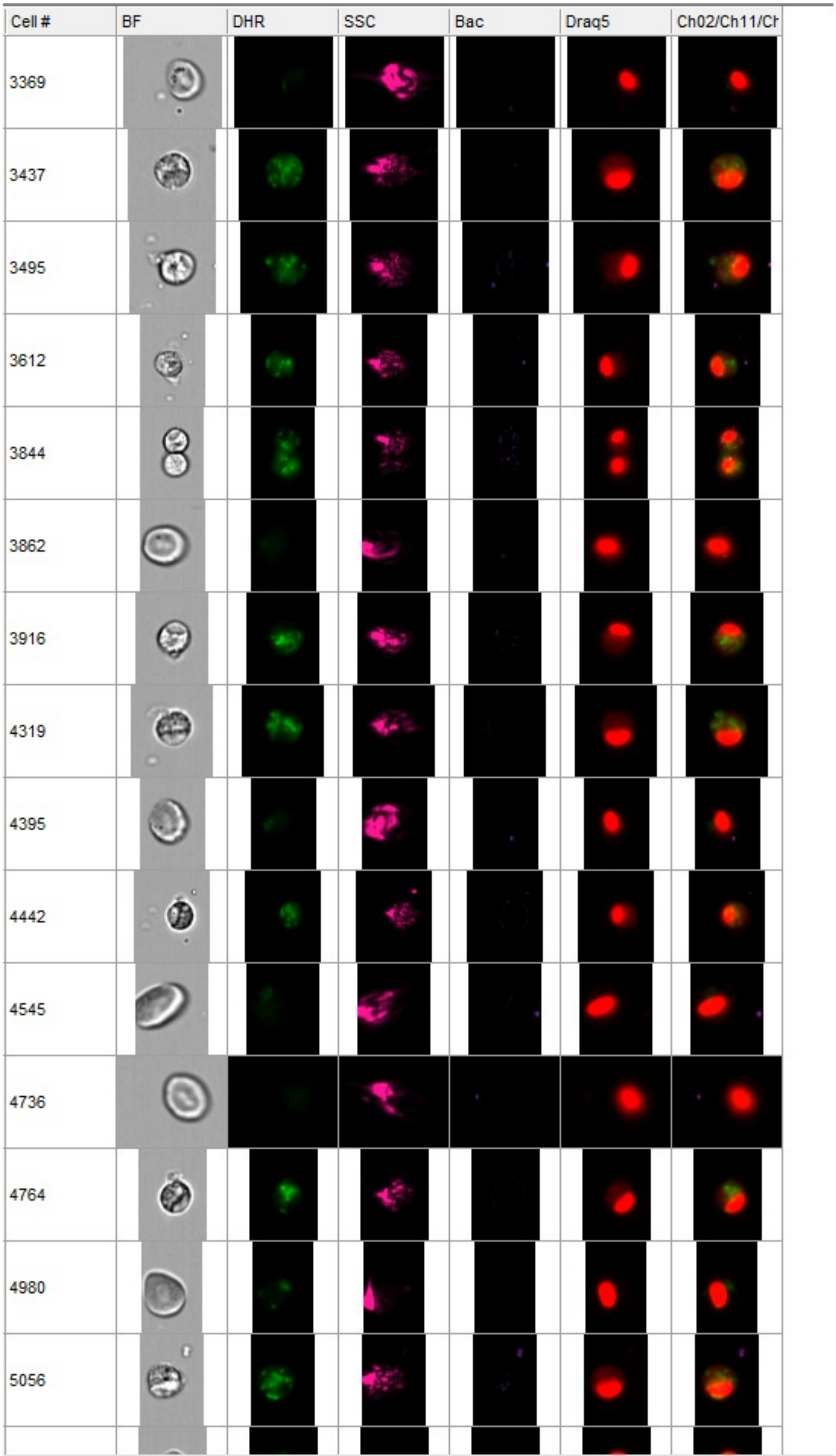
Cell #
BF
DHR
SSC
Bac
Draq5
Ch02/Ch11/Ch
3369
3437
3495
3612
3844
3862
3916
4319
4395
4442
4545
4736
4764
4980
5056

*Dr*21_P   Professional phagocytes

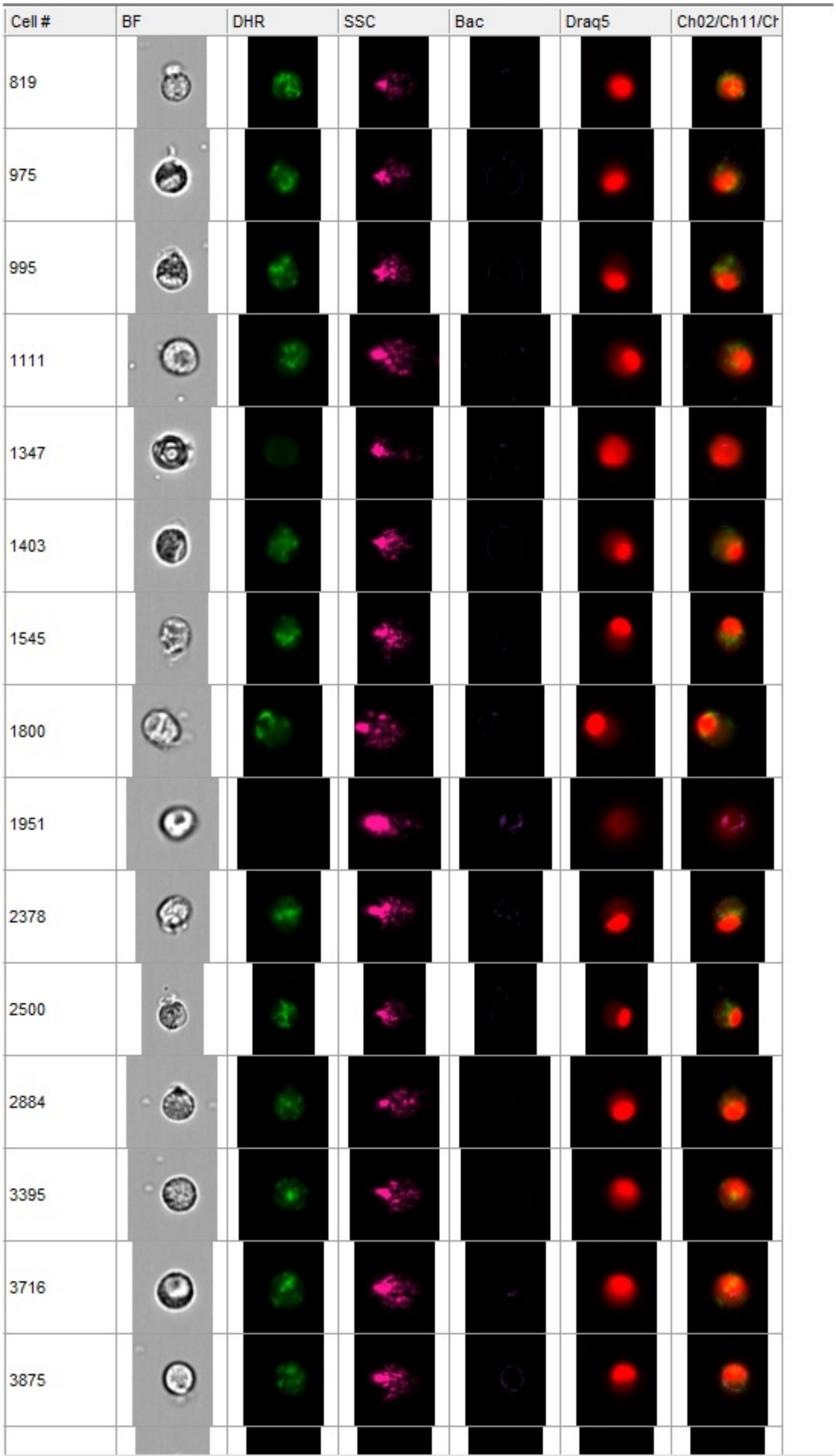

*Dr*22_P

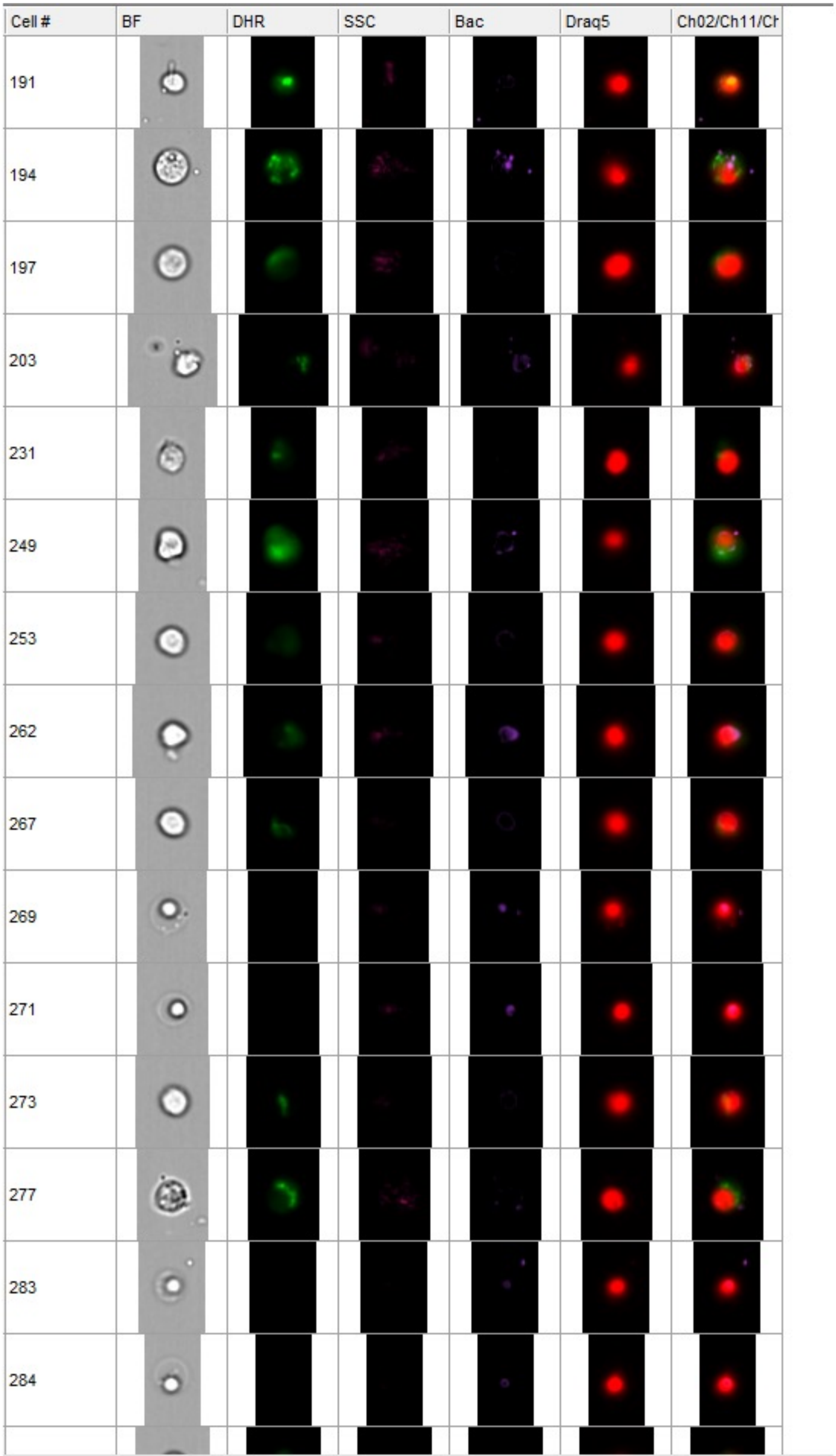
Cell #
BF
DHR
SSC
Bac
Draq5
Ch02/Ch11/Ch
191
194
197
203
231
249
253
262
267
269
271
273
277
283
284

*Dr*23_P

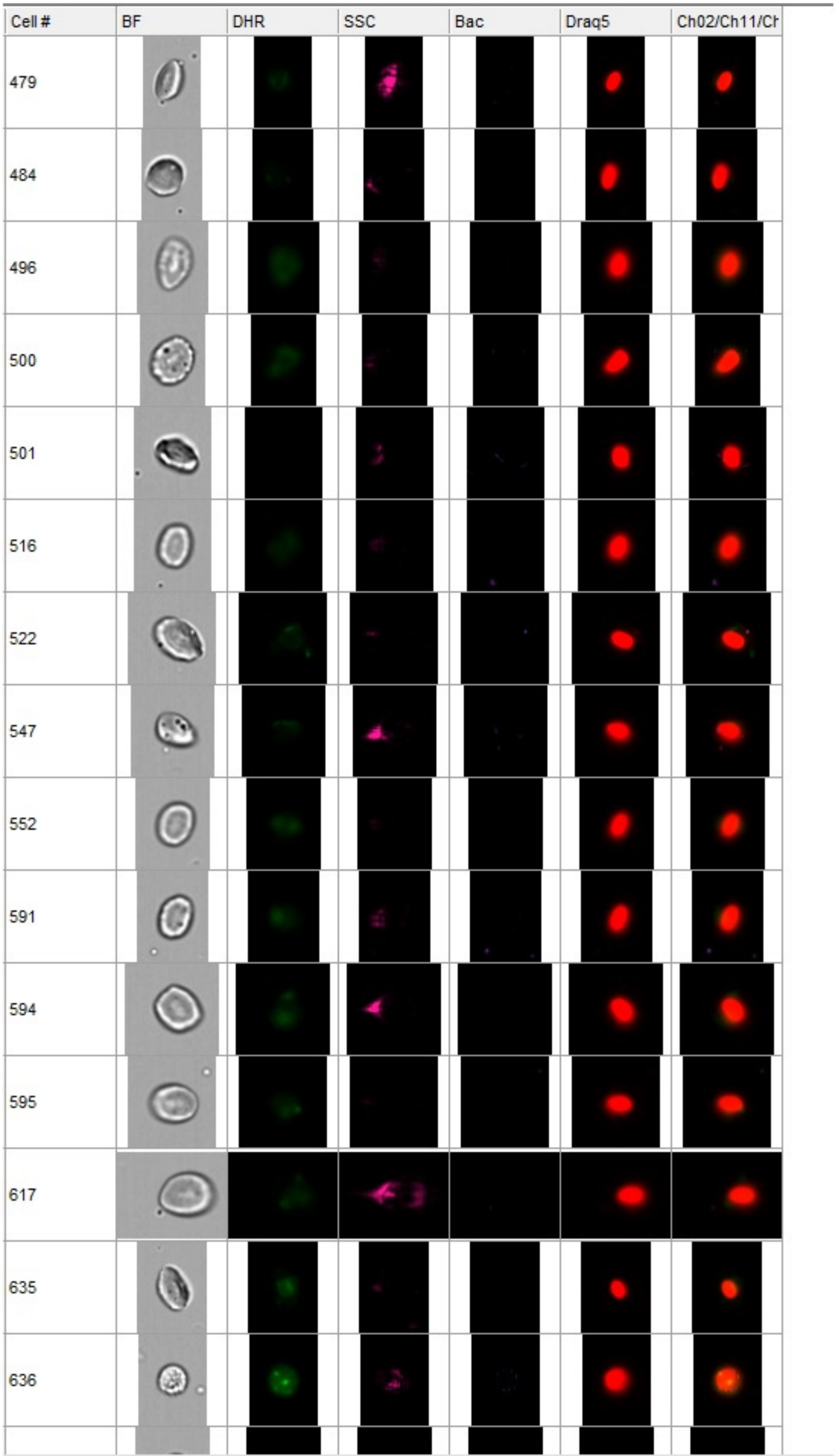
Cell #
BF
DHR
SSC
Bac
Draq5
Ch02/Ch11/Ch
479
484
496
500
501
516
522
547
552
591
594
595
617
635
636

*Dr*24_P

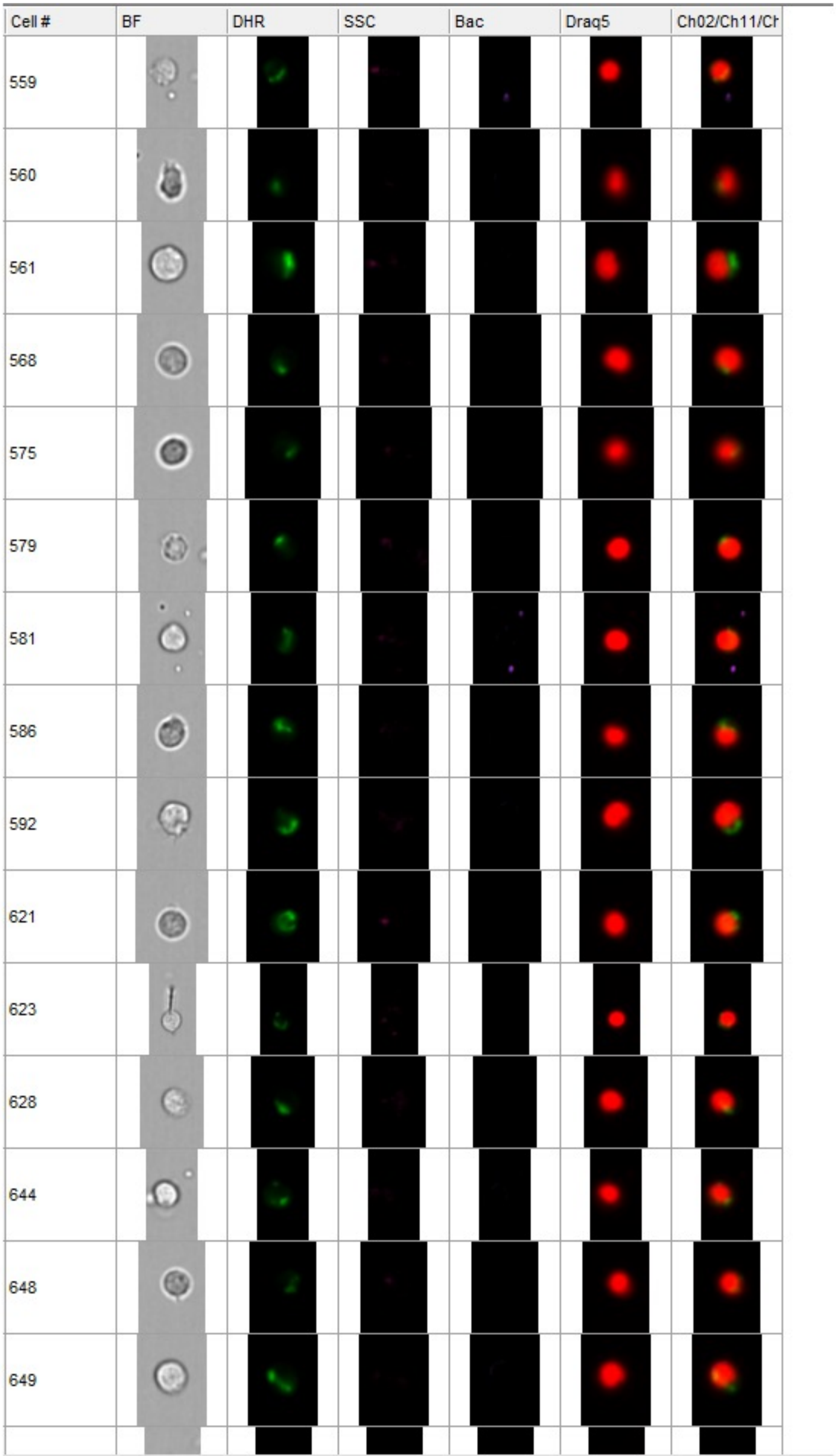

Cell #
BF
DHR
SSC
Bac
Draq5
Ch02/Ch11/Ch
559
560
561
568
575
579
581
586
592
621
623
628
644
648
649

*Dr25*_P

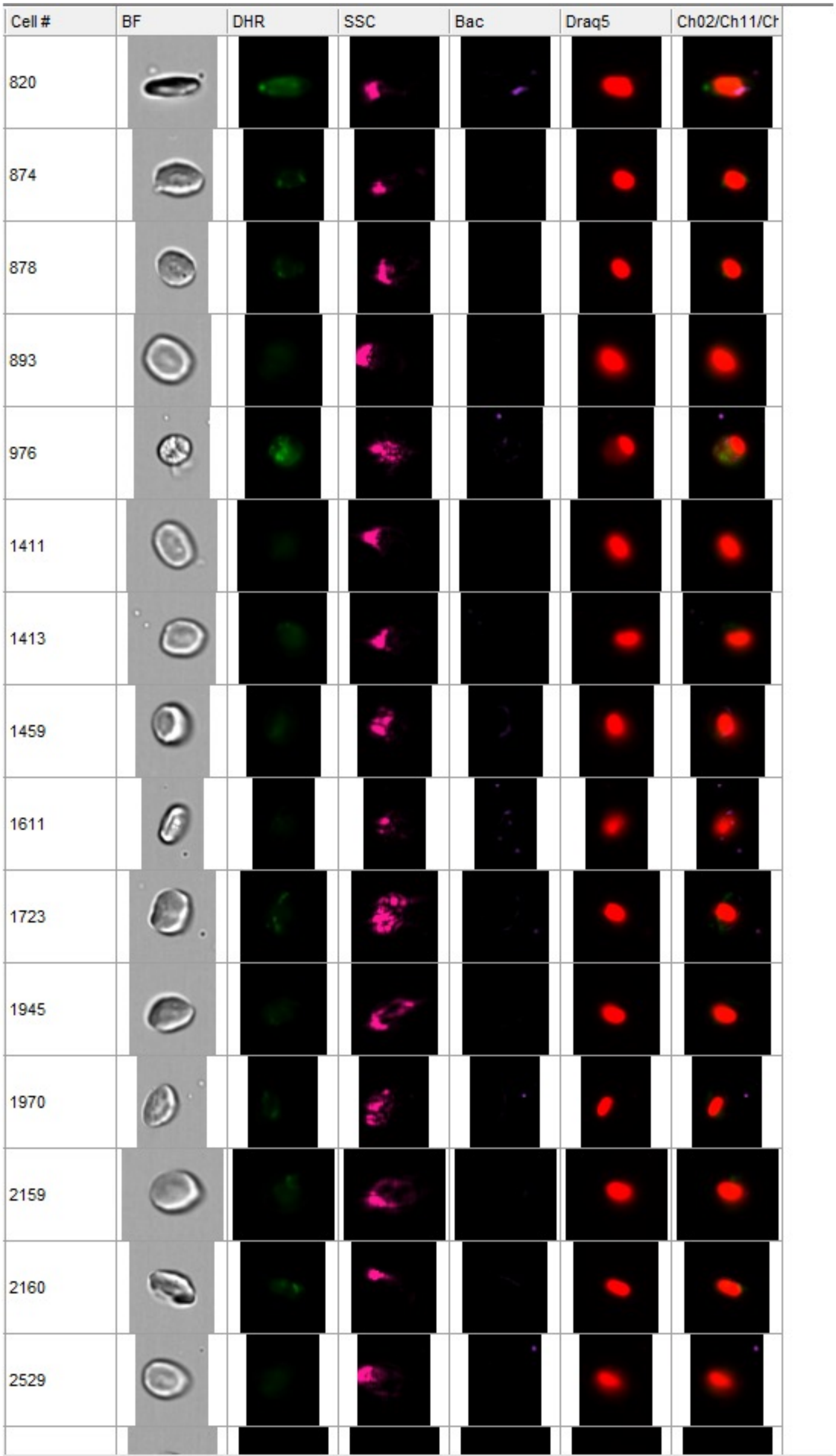
Cell #
BF
DHR
SSC
Bac
Draq5
Ch02/Ch11/Ch
820
874
878
893
976
1411
1413
1459
1611
1723
1945
1970
2159
2160
2529

*Dr*26_P

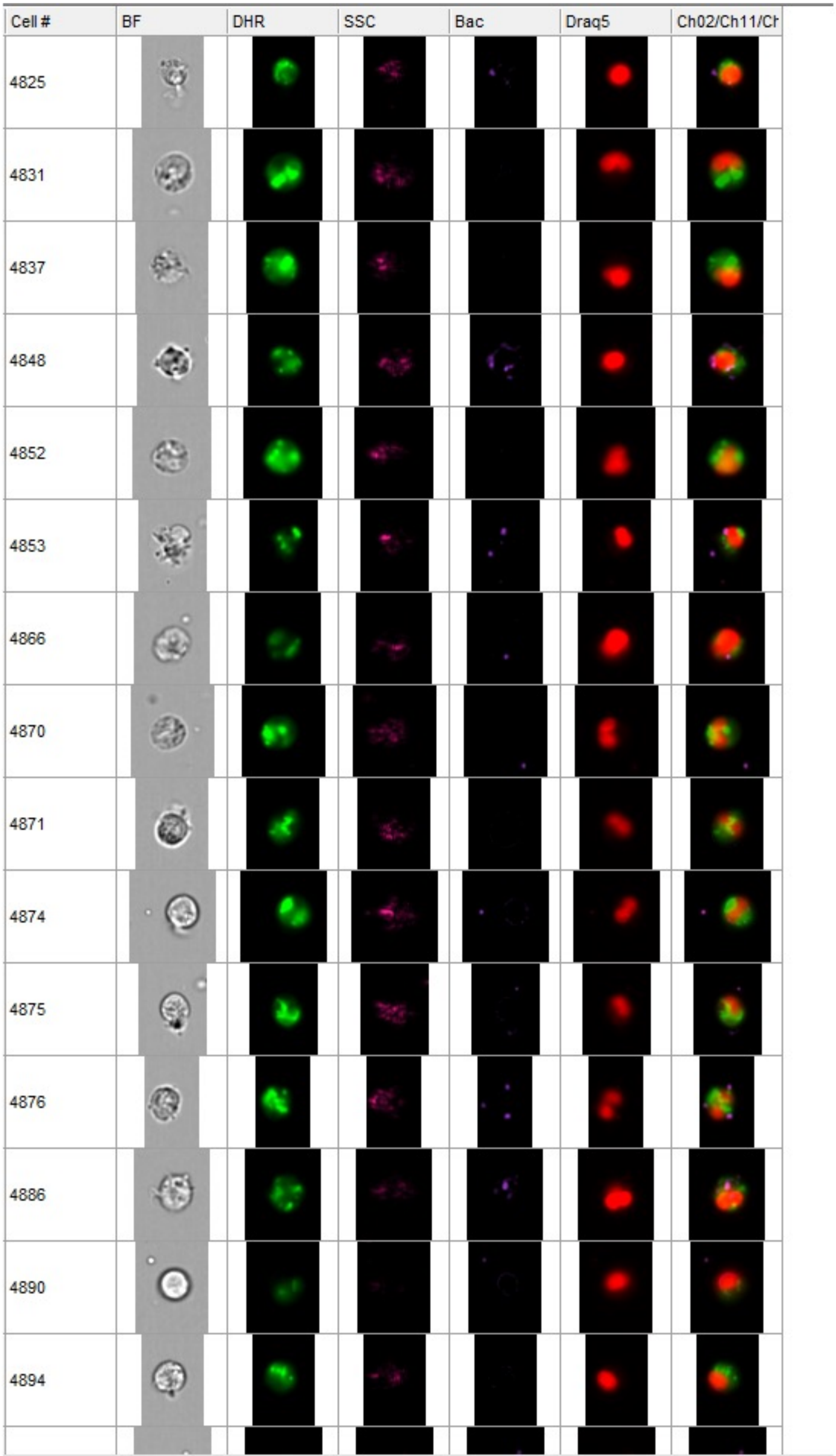
Cell #
BF
DHR
SSC
Bac
Draq5
Ch02/Ch11/Ch
4825
4831
4837
4848
4852
4853
4866
4870
4871
4874
4875
4876
4886
4890
4894
