# Supplementary material for: Image3C, a multimodal image-based and label-independent integrative method for single-cell analysis
Source: eLife. 2021 Jul 21;10:e65372. doi: 10.7554/eLife.65372 (PMC8370771; doi:10.7554/eLife.65372)

Apple snail
Homeostasis
Cell Morphology

Cell Gallery

Cluster
*Pc*1

Agranular large
hemocytes

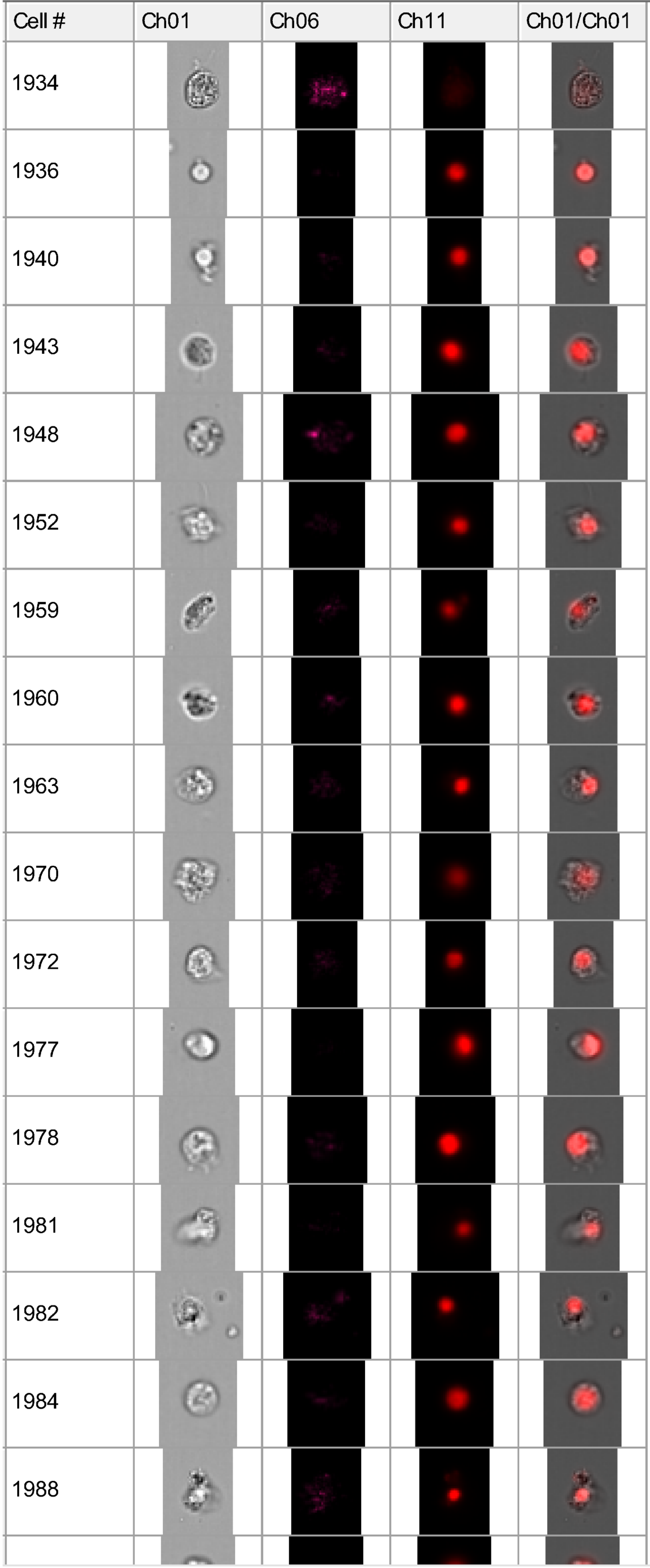

Cluster
Pc2

Intermediate
hemocytes

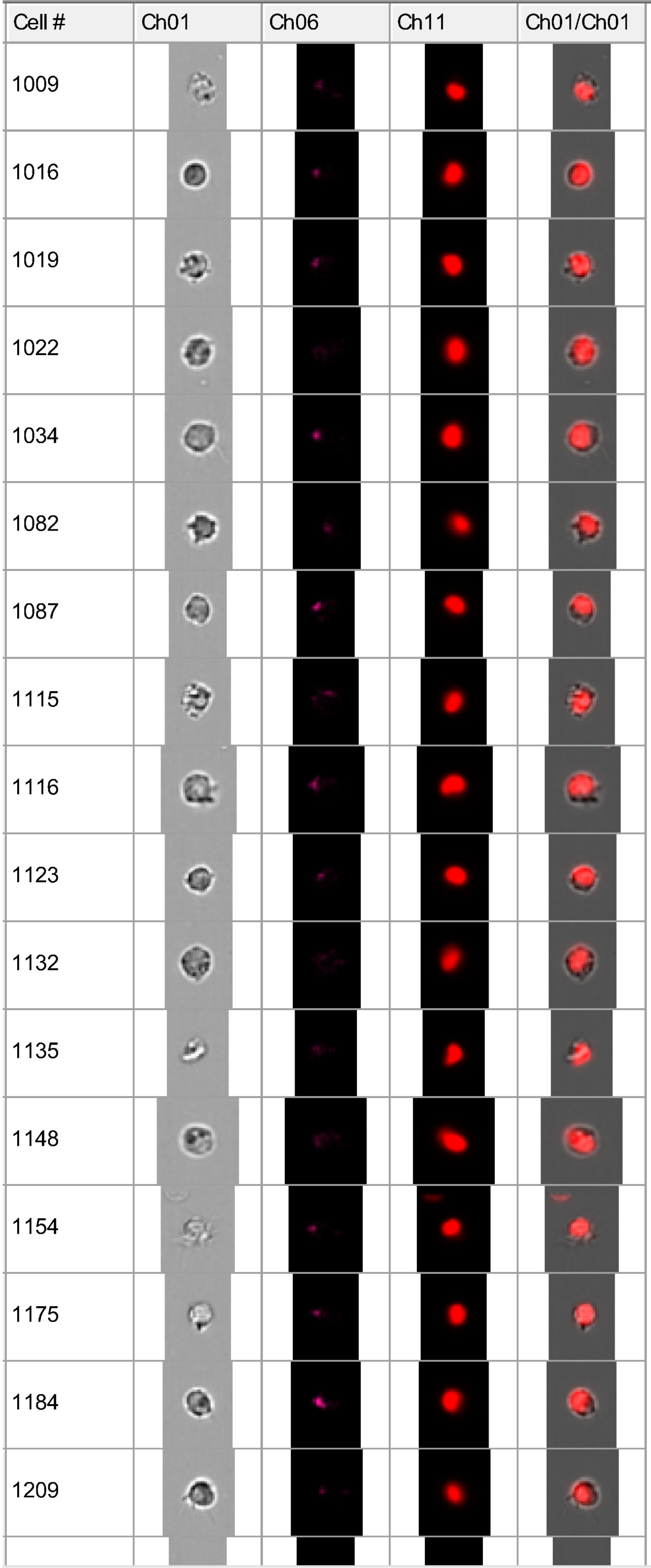

Cluster
*Pc*3

Intermediate
hemocytes

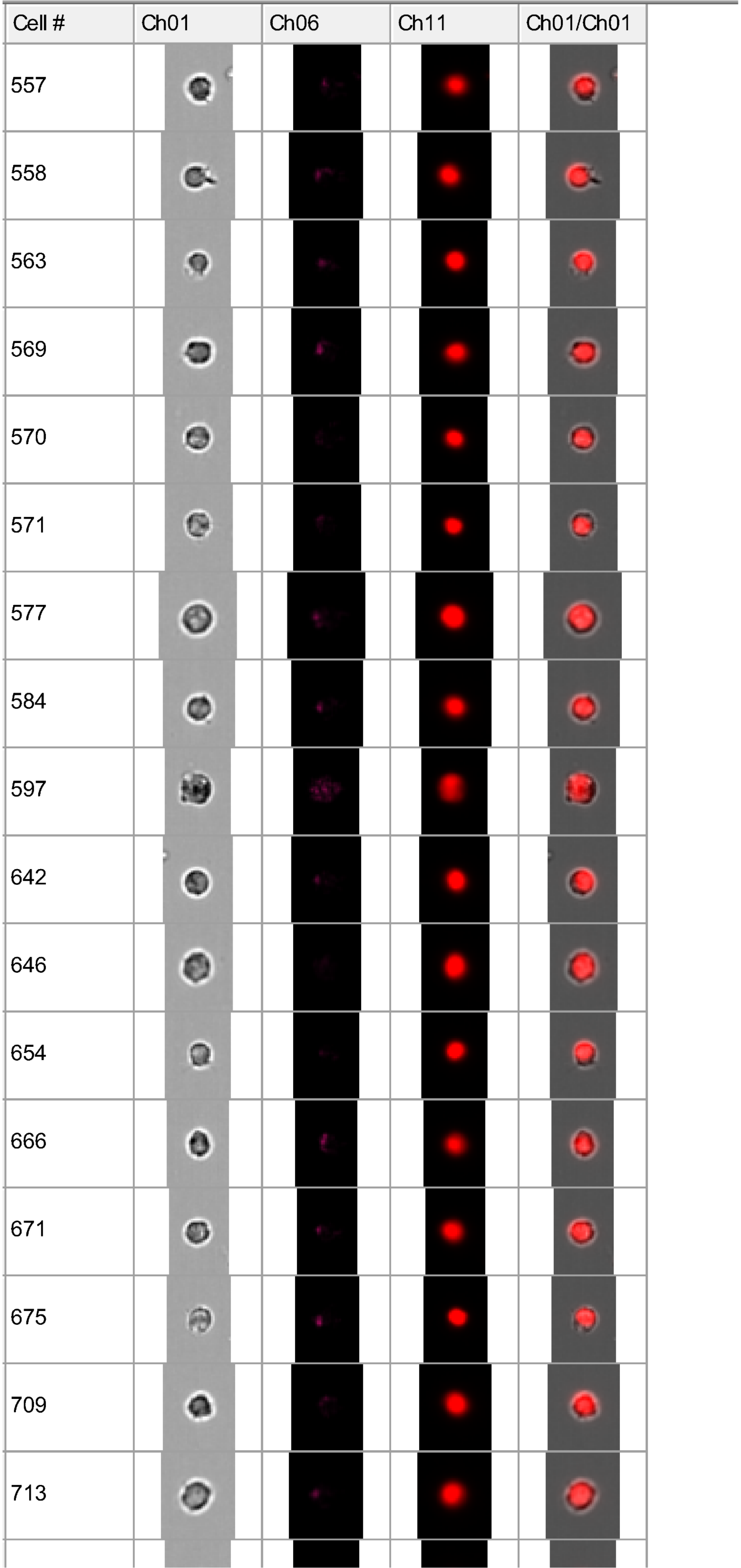

Cluster
*Pc*4

Blast-like
hemocytes

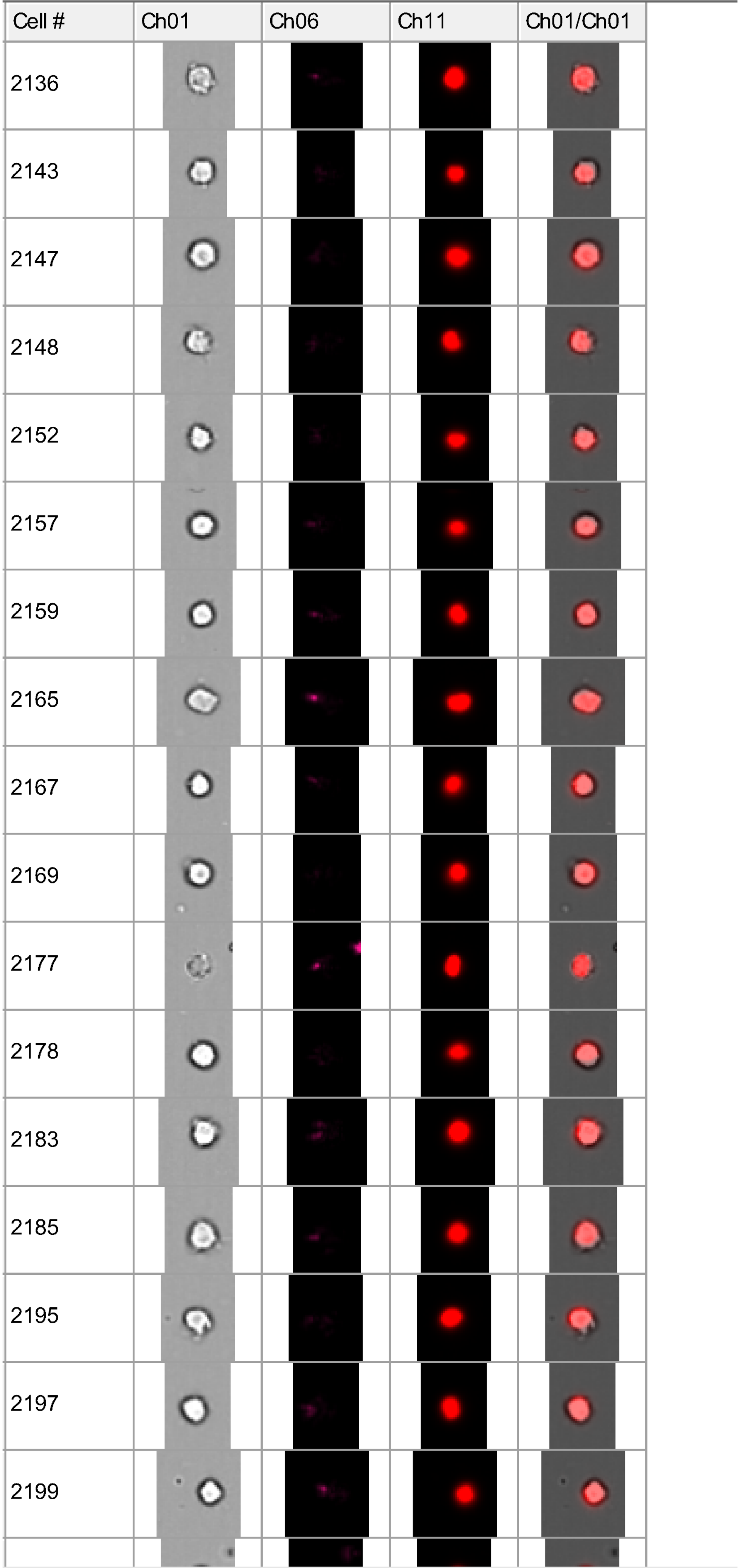

Cluster
Pc5

Doublets and dead cells

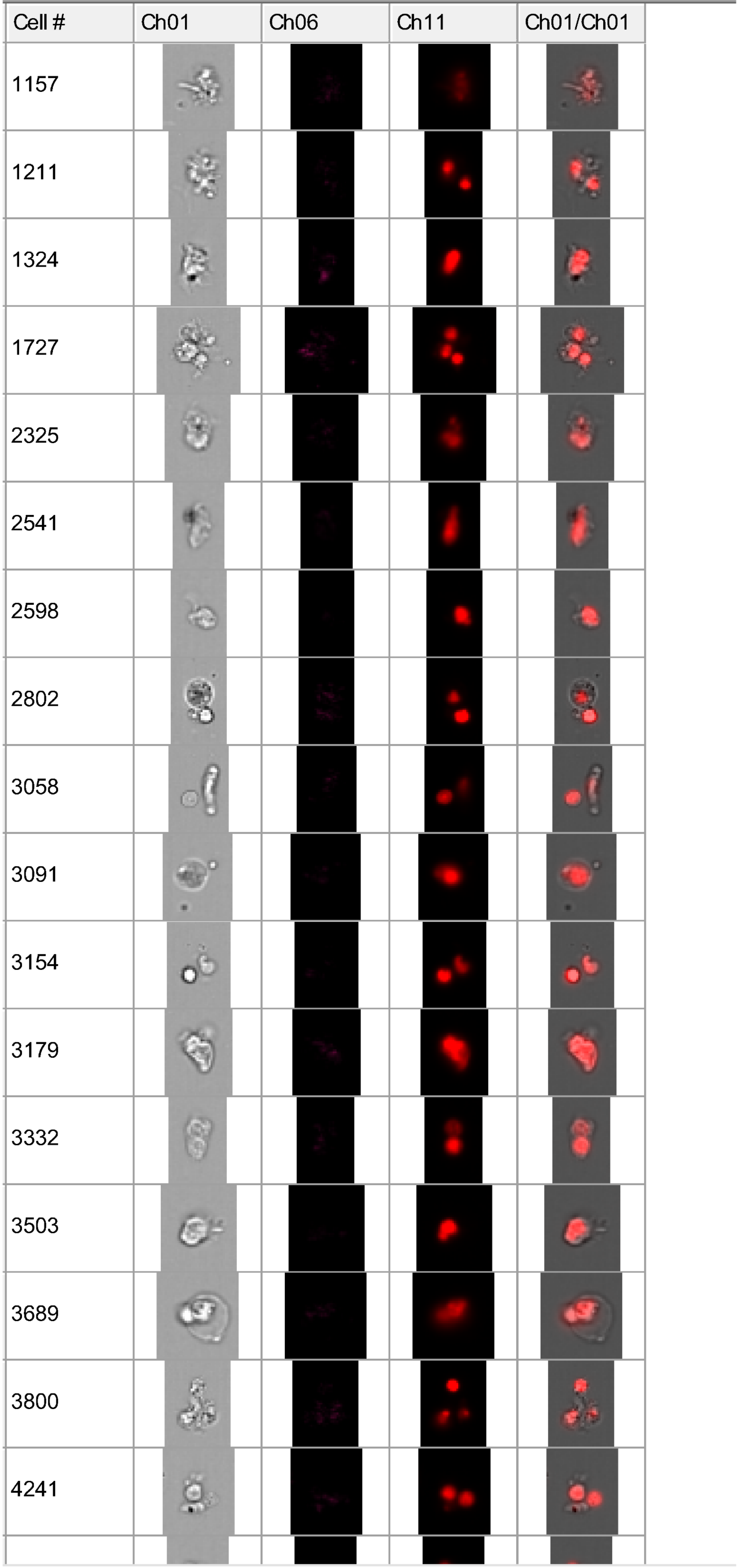

Cluster
*Pc*6

Granular large hemocytes

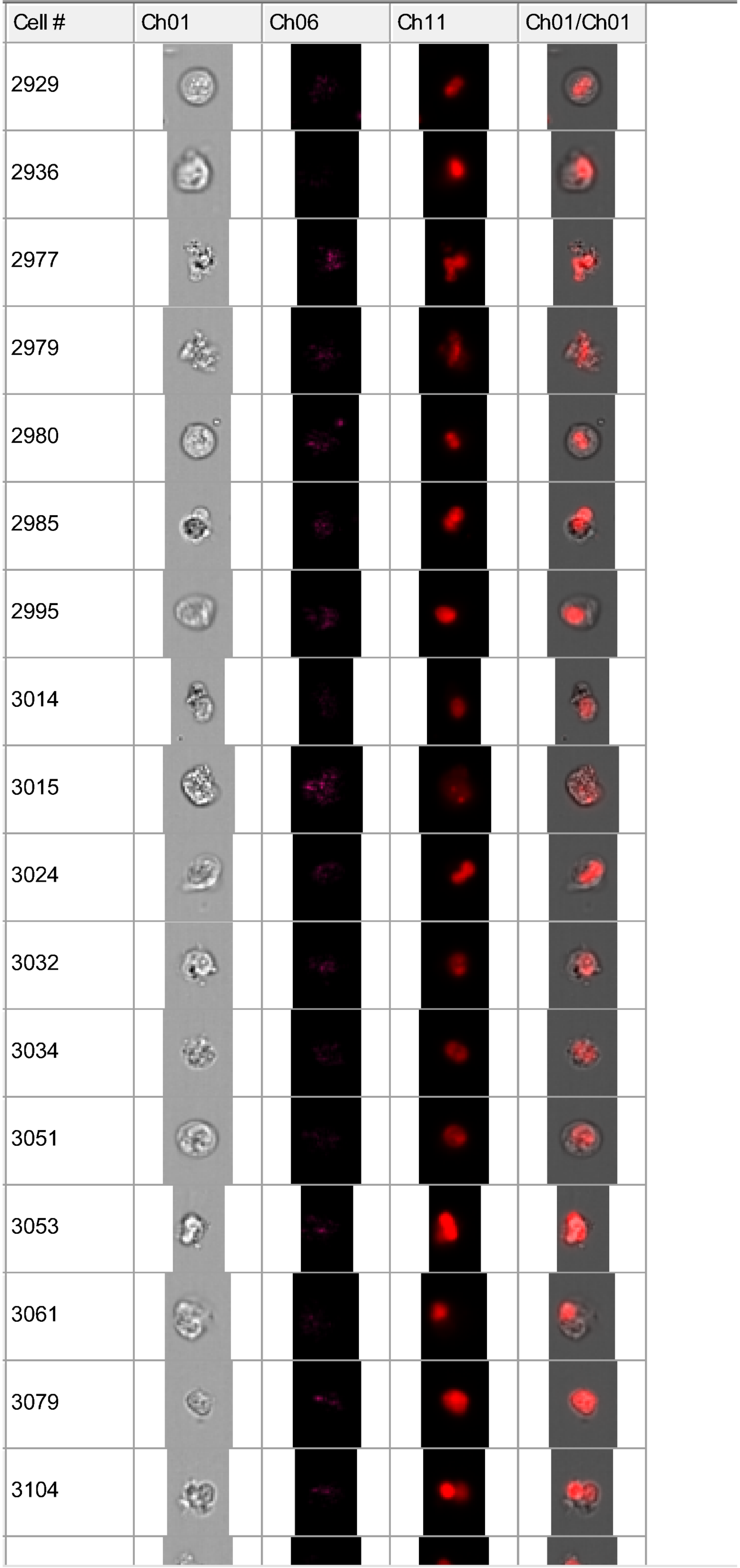

Cluster
Pc7

Agranular large hemocytes

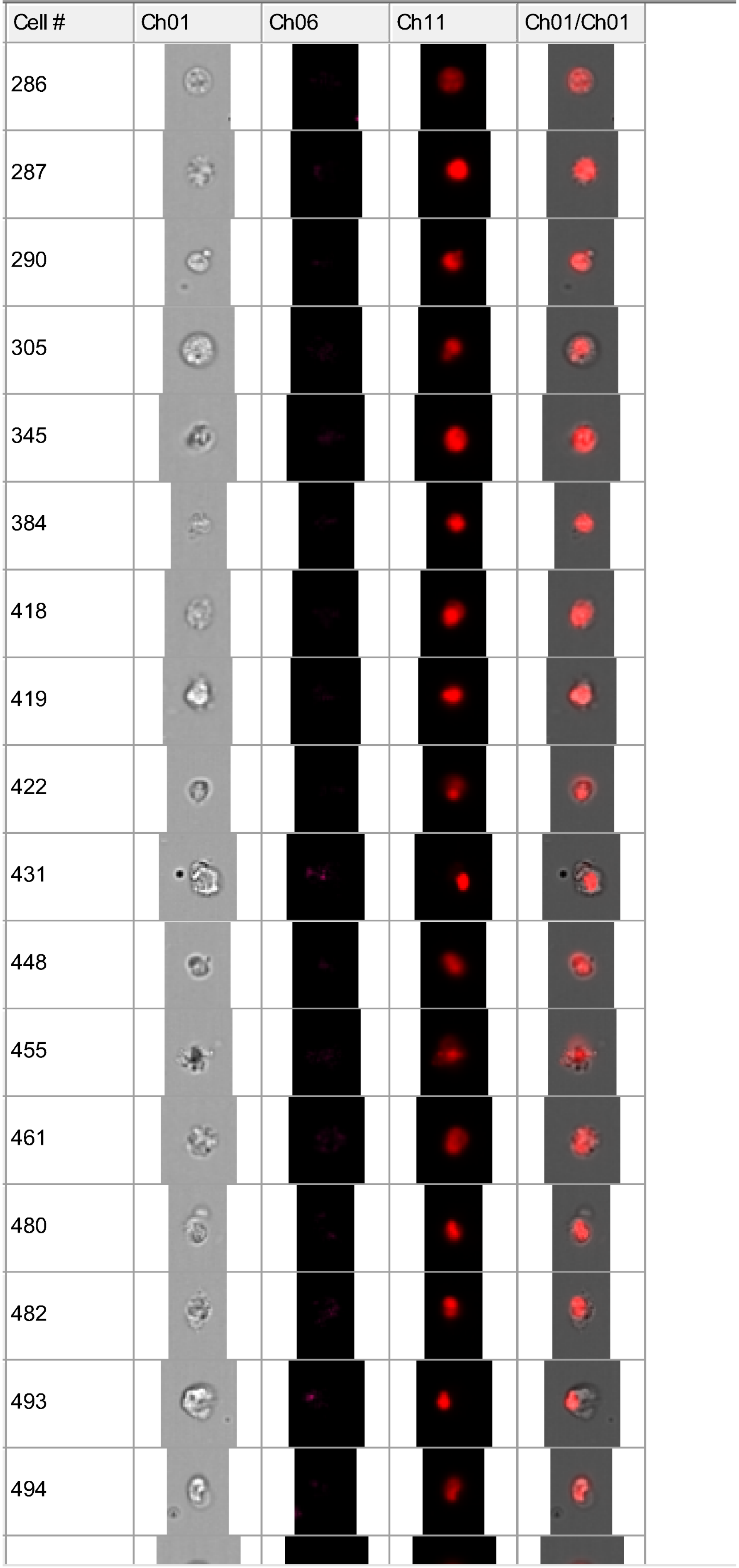

Cluster
*Pc*8

Doublets and dead cells

Cell #	Ch01	Ch06	Ch11	Ch01/Ch01
1207	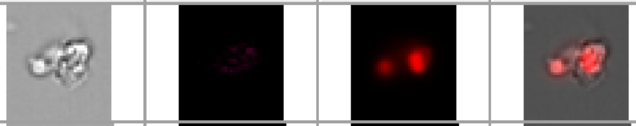			
1210	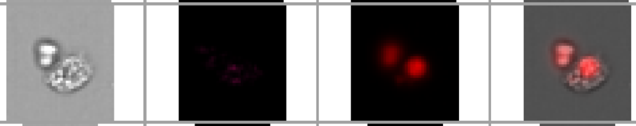			
1229	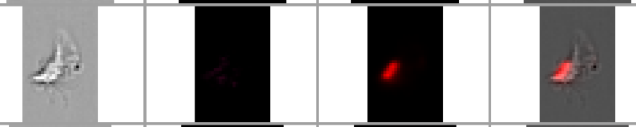			
1232	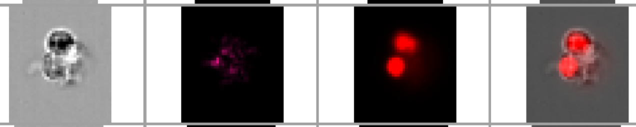			
1254	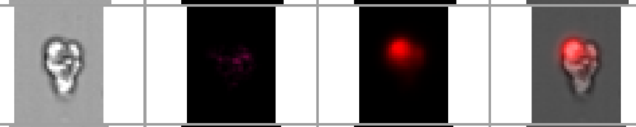			
1265	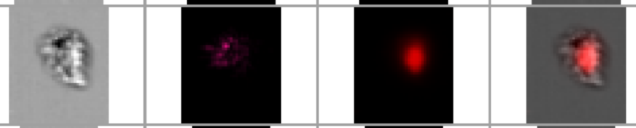			
1332	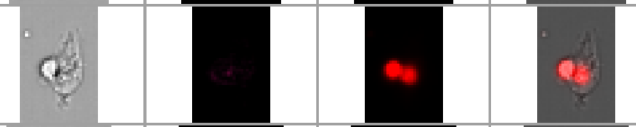			
1379	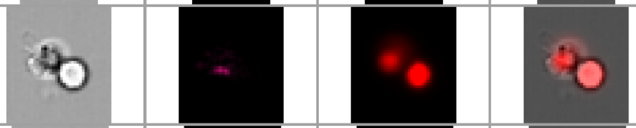			
1474	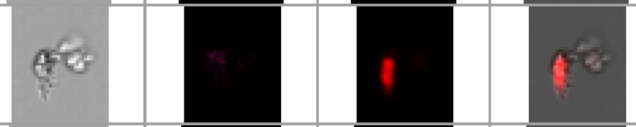			
1484	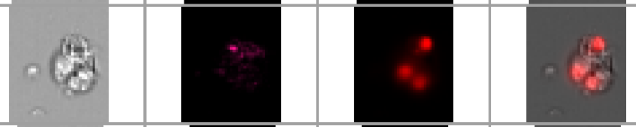			
1496	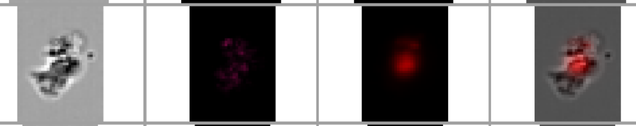			
1522	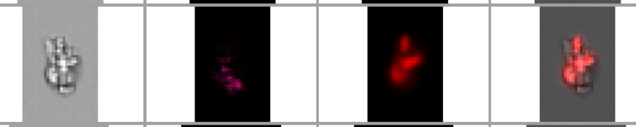			
1524	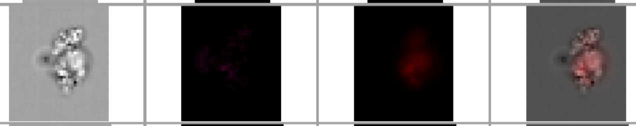			
1531	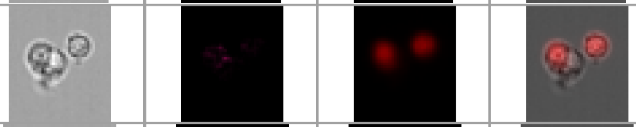			
1534	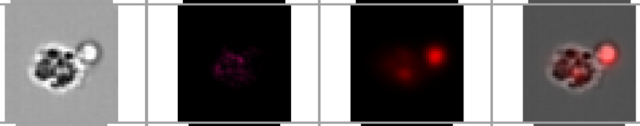			
1548	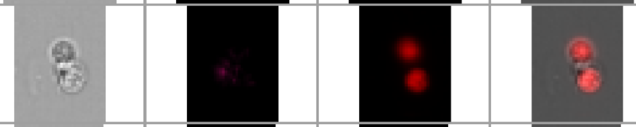			
1633	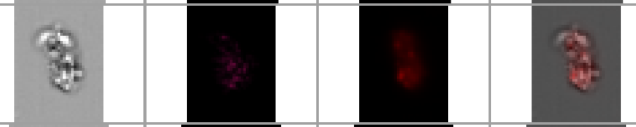			
	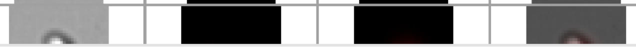			

Cluster
*Pc*9

Agranular large
hemocytes

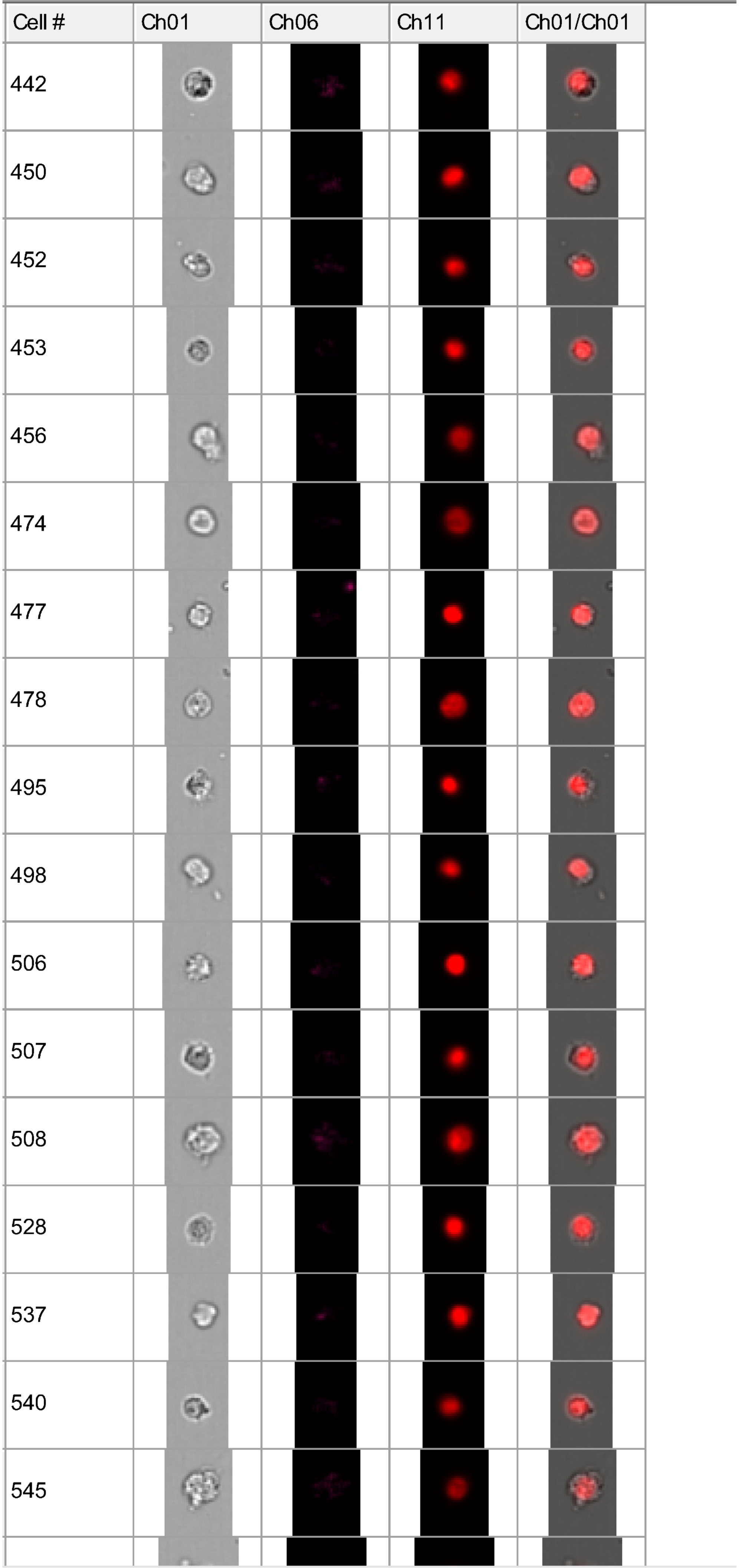

Supplement: Supplementary file 8. — Representative cell images belonging to each individual cluster identified by Image3C for snail hemocytes in homeostasis condition are shown. Ch01 is brightfield, Ch06 is side scatter signal, and Ch11 is Draq5 (nuclear staining). Merge represents the overlay of Ch01, Ch06, and Ch11. [file elife-65372-supp8.pdf]
