# Supplementary material for: Image3C, a multimodal image-based and label-independent integrative method for single-cell analysis
Source: eLife. 2021 Jul 21;10:e65372. doi: 10.7554/eLife.65372 (PMC8370771; doi:10.7554/eLife.65372)
Supplement: Supplementary file 9. — Results of negative binomial regression analysis comparing cluster relative abundance between phagocytosis samples (CTV-S. aureus) and phagocytosis inhibited with EDTA samples (CTV-S. aureus + EDTA) in the apple snail P. canaliculata phagocytosis experiment. FC: fold change; CPM: count per million; LR: likelihood ratio; FDR: false discovery rate. Relative graph is reported in Figure 5B. [file elife-65372-supp9.docx]

**Supplementary File 9: Phagocytosis vs phagocytosis inhibited with EDTA on P. canaliculata hemocytes**

Results of negative binomial regression analysis comparing cluster relative abundance between phagocytosis samples (CTV-S. aureus) vs phagocytosis inhibited with EDTA samples (CTV-S. aureus + EDTA) in the apple snail P. canaliculata phagocytosis experiment. FC is Fold Change, CPM is Count Per Million, LR is Likelihood Ratio, FDR is Fold Discovery Rate. Relative graph is reported in Figure 5B.

| **Cluster ID** | **logFC** | **logCPM** | **LR** | **PValue** | **FDR** |
| --- | --- | --- | --- | --- | --- |
| *Pc*1_P | 1.21972 | 16.42389 | 23.86393 | 1.0E-06 | 4.1E-06 |
| *Pc*2_P | 1.52130 | 16.31424 | 23.42745 | 1.3E-06 | 4.3E-06 |
| *Pc*5_P | 3.50692 | 11.96025 | 19.19534 | 1.2E-05 | 2.6E-05 |
| *Pc*6_P | 2.00062 | 13.66811 | 21.45211 | 3.6E-06 | 9.1E-06 |
| *Pc*7_P | 1.17845 | 15.71918 | 15.65951 | 7.6E-05 | 1.5E-04 |
| *Pc*8_P | 0.91220 | 14.51336 | 5.60883 | 1.8E-02 | 2.4E-02 |
| *Pc*9_P | 1.91957 | 14.24789 | 21.73770 | 3.1E-06 | 8.9E-06 |
| *Pc*10_P | -0.95771 | 16.55159 | 15.15146 | 9.9E-05 | 1.8E-04 |
| *Pc*11_P | -2.21453 | 17.04466 | 66.60728 | 3.3E-16 | 6.6E-15 |
| *Pc*12_P | 1.92022 | 13.48376 | 27.01612 | 2.0E-07 | 1.0E-06 |
| *Pc*13_P | 1.15586 | 13.78276 | 11.69042 | 6.3E-04 | 9.7E-04 |
| *Pc*14_P | 1.74206 | 17.72100 | 51.24411 | 8.2E-13 | 5.4E-12 |
| *Pc*16_P | 1.64586 | 13.23961 | 10.80603 | 1.0E-03 | 1.4E-03 |
| *Pc*17_P | 3.13469 | 13.98527 | 55.58824 | 8.9E-14 | 8.9E-13 |
| *Pc*20_P | -0.82885 | 16.67206 | 12.56812 | 3.9E-04 | 6.5E-04 |
