# Supplementary material for: Image3C, a multimodal image-based and label-independent integrative method for single-cell analysis
Source: eLife. 2021 Jul 21;10:e65372. doi: 10.7554/eLife.65372 (PMC8370771; doi:10.7554/eLife.65372)
Supplement: Supplementary file 10. — Results of negative binomial regression analysis comparing cluster relative abundance between phagocytosis samples (CTV-S. aureus) and phagocytosis inhibited with ice samples (CTV-S. aureus + Ice) in the apple snail P. canaliculata phagocytosis experiment. FC: fold change; CPM: count per million; LR: likelihood ratio; FDR: false discovery rate. Relative graph is reported in Figure 5—figure supplement 2. [file elife-65372-supp10.docx]

**Supplementary File 10: Phagocytosis vs phagocytosis inhibited with ice on P. canaliculata hemocytes**

Results of negative binomial regression analysis comparing cluster relative abundance between phagocytosis samples (CTV-S. aureus) vs phagocytosis inhibited with ice samples (CTV-S. aureus + Ice) in the apple snail P. canaliculata phagocytosis experiment. FC is Fold Change, CPM is Count Per Million, LR is Likelihood Ratio, FDR is Fold Discovery Rate. Relative graph is reported in Figure 5-figure supplement 2.

| **Cluster ID** | **logFC** | **logCPM** | **LR** | **PValue** | **FDR** |
| --- | --- | --- | --- | --- | --- |
| *Pc*17_P | 2.50037 | 13.98527 | 37.29469 | 1.02E-09 | 2.03E-08 |
