# Supplementary material for: Image3C, a multimodal image-based and label-independent integrative method for single-cell analysis
Source: eLife. 2021 Jul 21;10:e65372. doi: 10.7554/eLife.65372 (PMC8370771; doi:10.7554/eLife.65372)
Supplement: Supplementary file 11. — Representative cell images belonging to each individual cluster identified by Image3C for snail hemocytes after phagocytosis assay are shown. Ch01 is brightfield, Ch02 is DHR signal (reactive oxygen species indicator), Ch06 is side scatter signal, Ch07 is CTV signal (S. aureus labeling), and Ch11 is Draq5 (nuclear staining). Merge represents the overlay of Ch02, Ch06, Ch07, and Ch11. [file elife-65372-supp11.pdf]

Apple snail Phagocytosis Experiment

Cell Gallery

*Pc*1_P

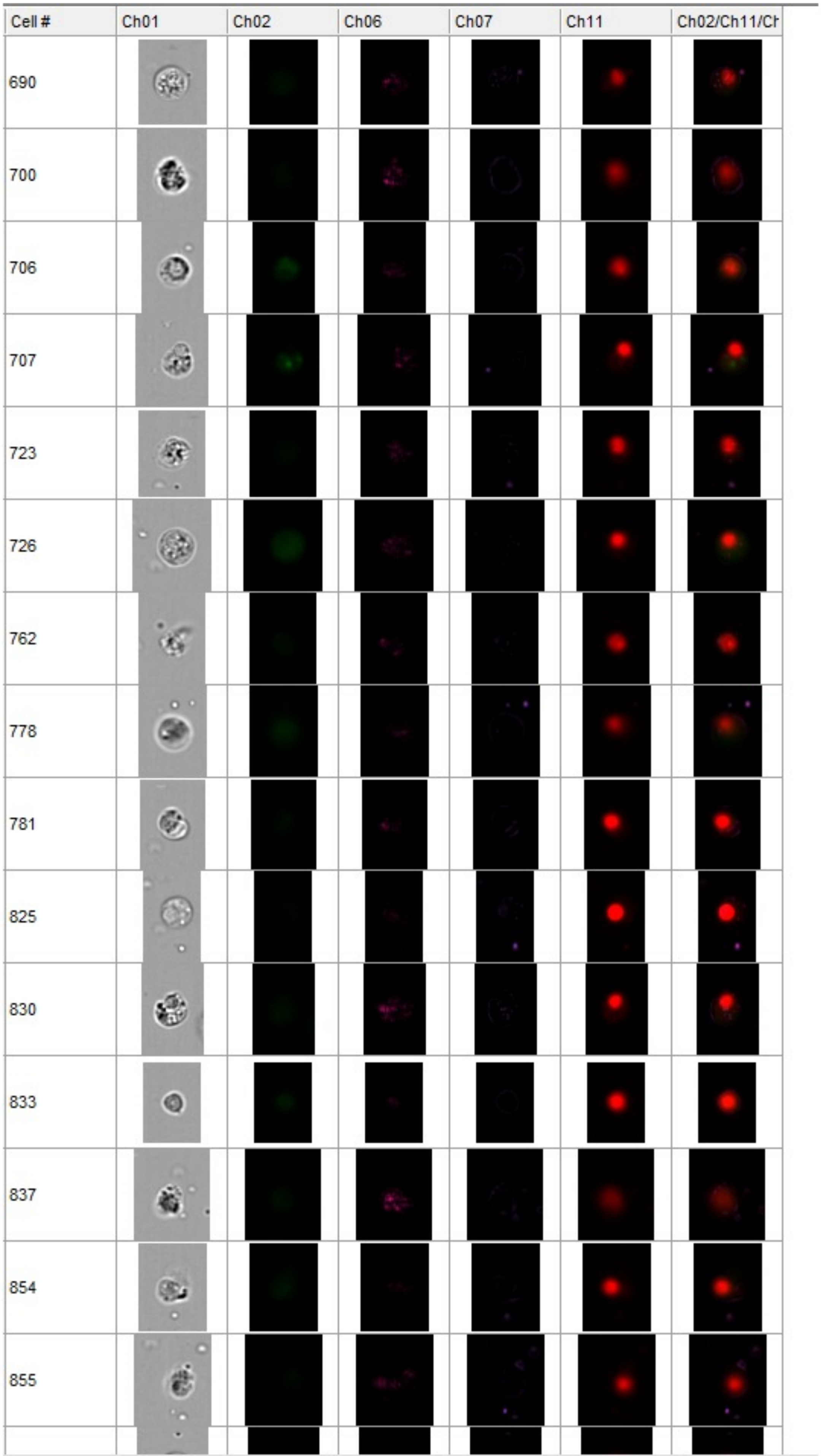
Cell #
Ch01
Ch02
Ch06
Ch07
Ch11
Ch02/Ch11/Ch
690
700
706
707
723
726
762
778
781
825
830
833
837
854
855

*Pc*2_P

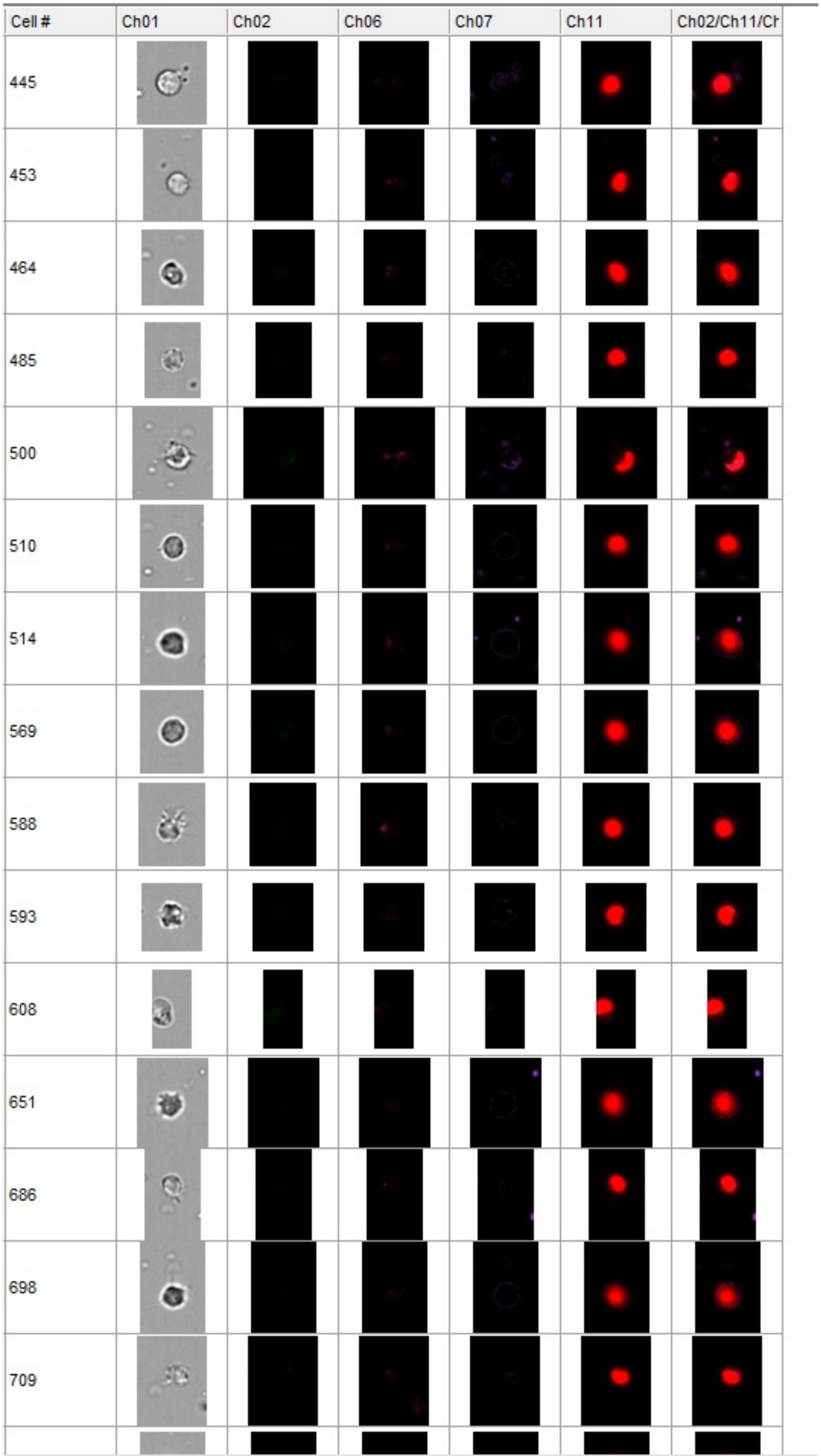

Cell #
Ch01
Ch02
Ch06
Ch07
Ch11
Ch02/Ch11/Ch
445
453
464
485
500
510
514
569
588
593
608
651
686
698
709

*Pc*3_P

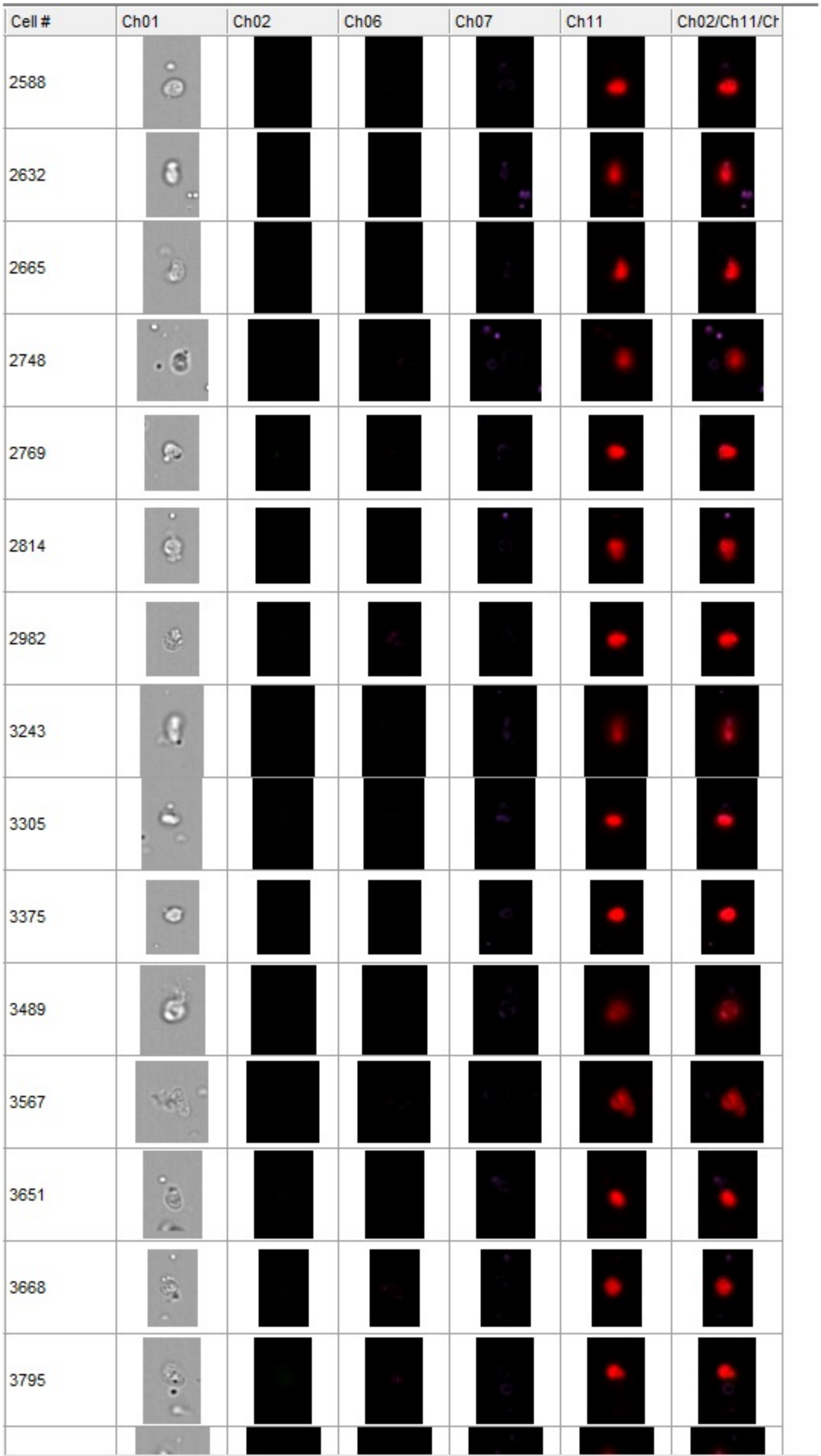
Cell #
Ch01
Ch02
Ch06
Ch07
Ch11
Ch02/Ch11/Ch
2588
2632
2665
2748
2769
2814
2982
3243
3305
3375
3489
3567
3651
3668
3795

*Pc*4_P

Cell #	Ch01	Ch02	Ch06	Ch07	Ch11	Ch02/Ch11/Ch
1235	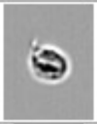	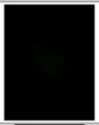	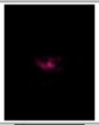	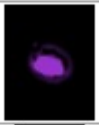	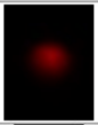	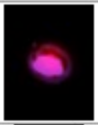
5722	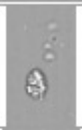	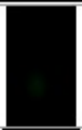	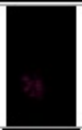	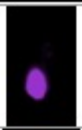	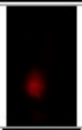	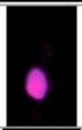
7916	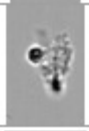	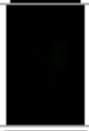	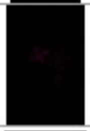	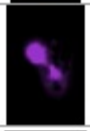	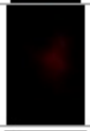	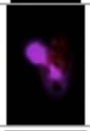
8446	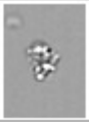	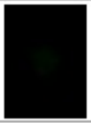	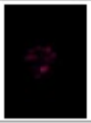	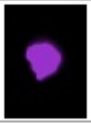	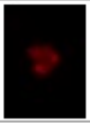	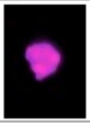

*Pc*5_P   Professional phagocytes

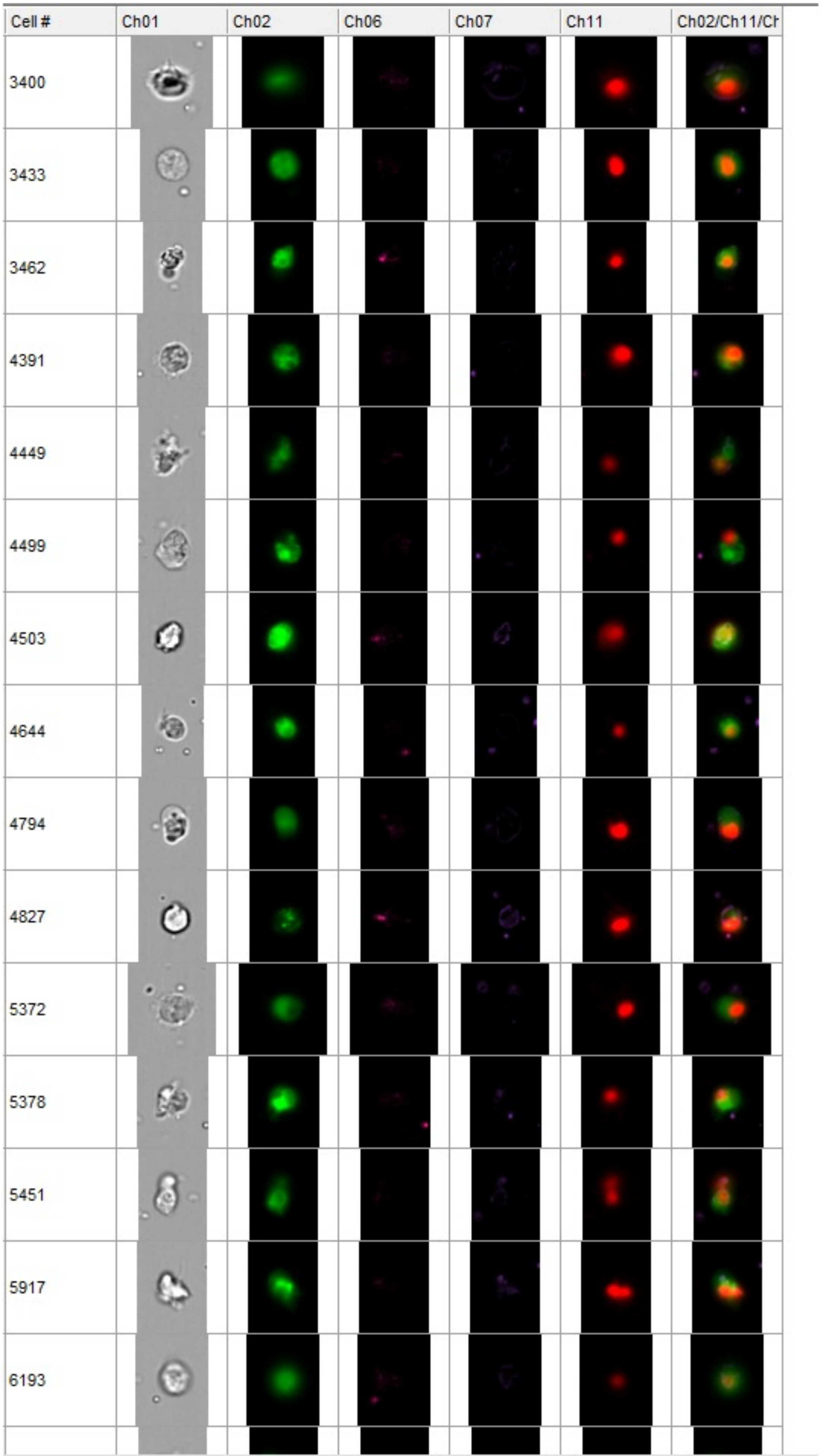

*Pc*6_P

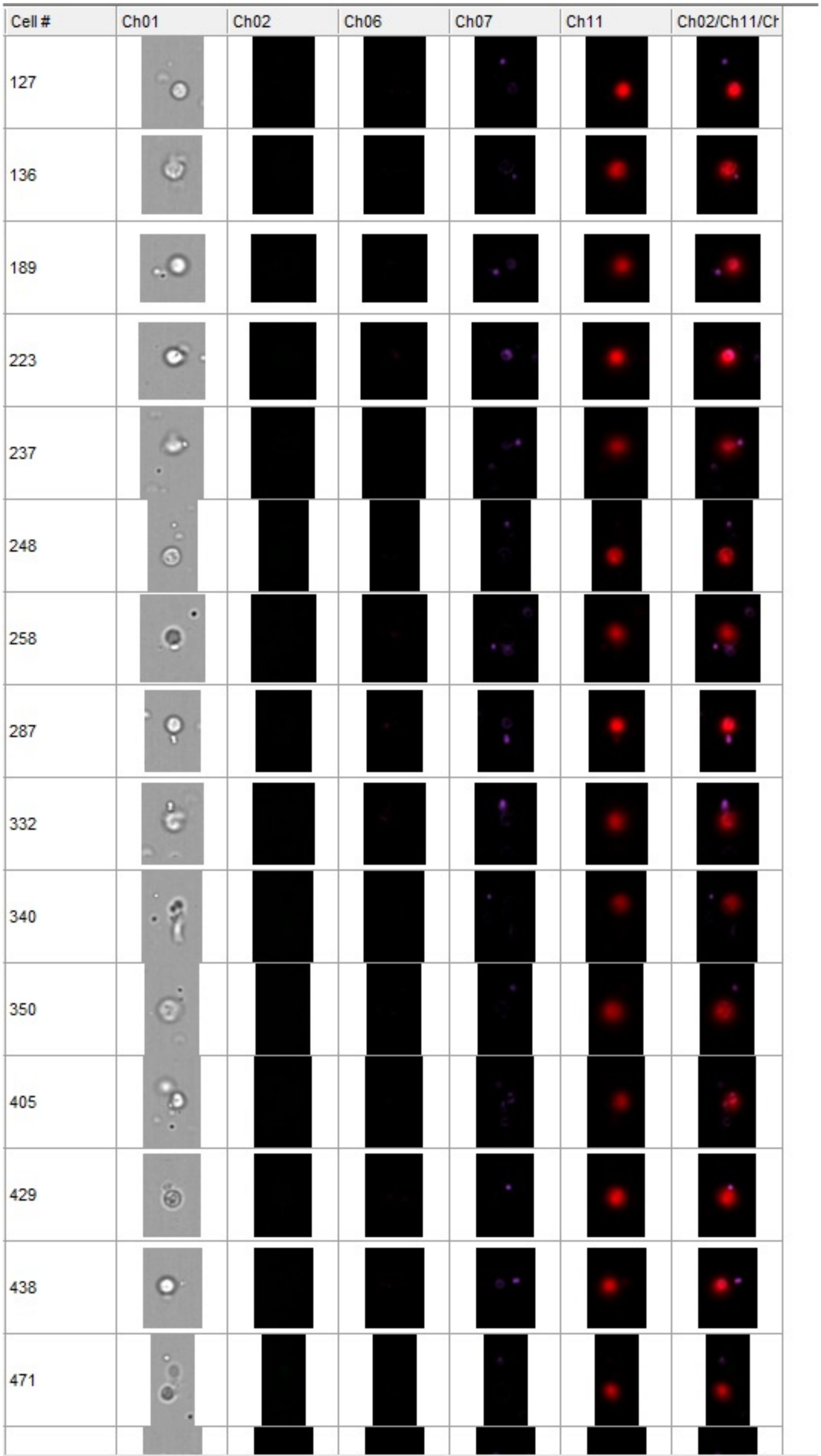
Cell #
Ch01
Ch02
Ch06
Ch07
Ch11
Ch02/Ch11/Ch
127
136
189
223
237
248
258
287
332
340
350
405
429
438
471

*Pc7*_P

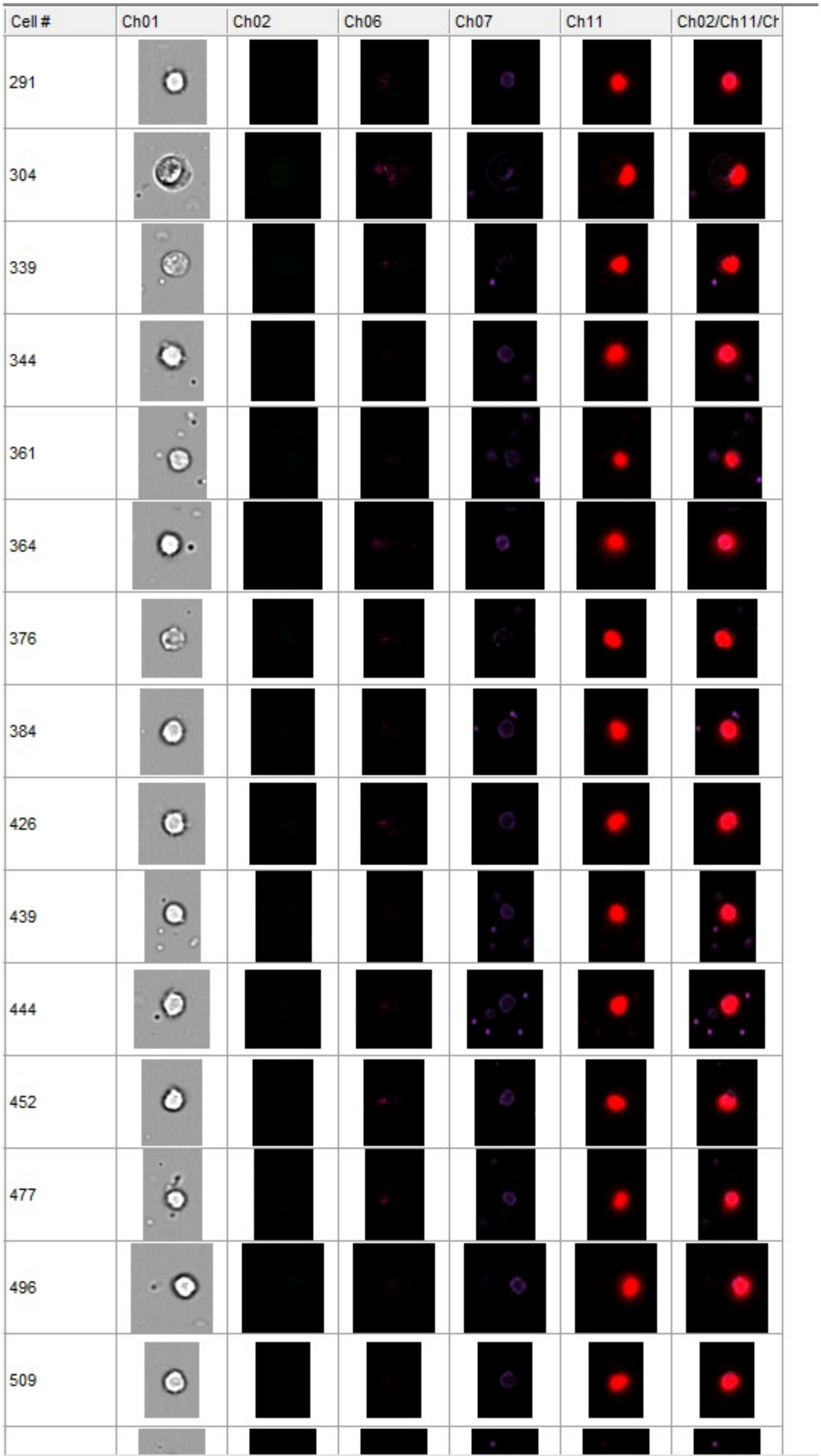
Cell #
Ch01
Ch02
Ch06
Ch07
Ch11
Ch02/Ch11/Ch
291
304
339
344
361
364
376
384
426
439
444
452
477
496
509

*Pc*8_P

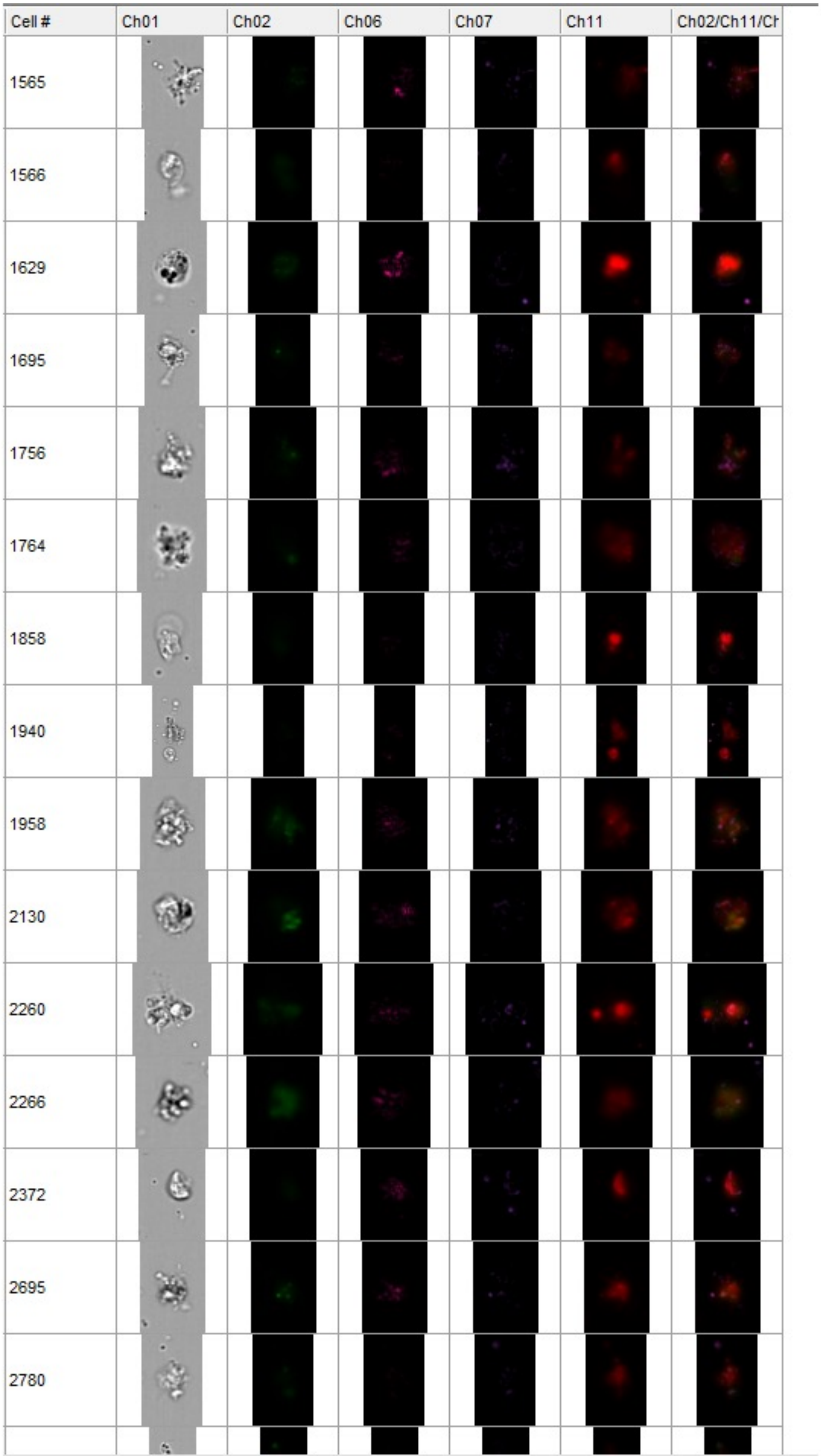

Cell #
Ch01
Ch02
Ch06
Ch07
Ch11
Ch02/Ch11/Ch
1565
1566
1629
1695
1756
1764
1858
1940
1958
2130
2260
2266
2372
2695
2780

*Pc*9_P

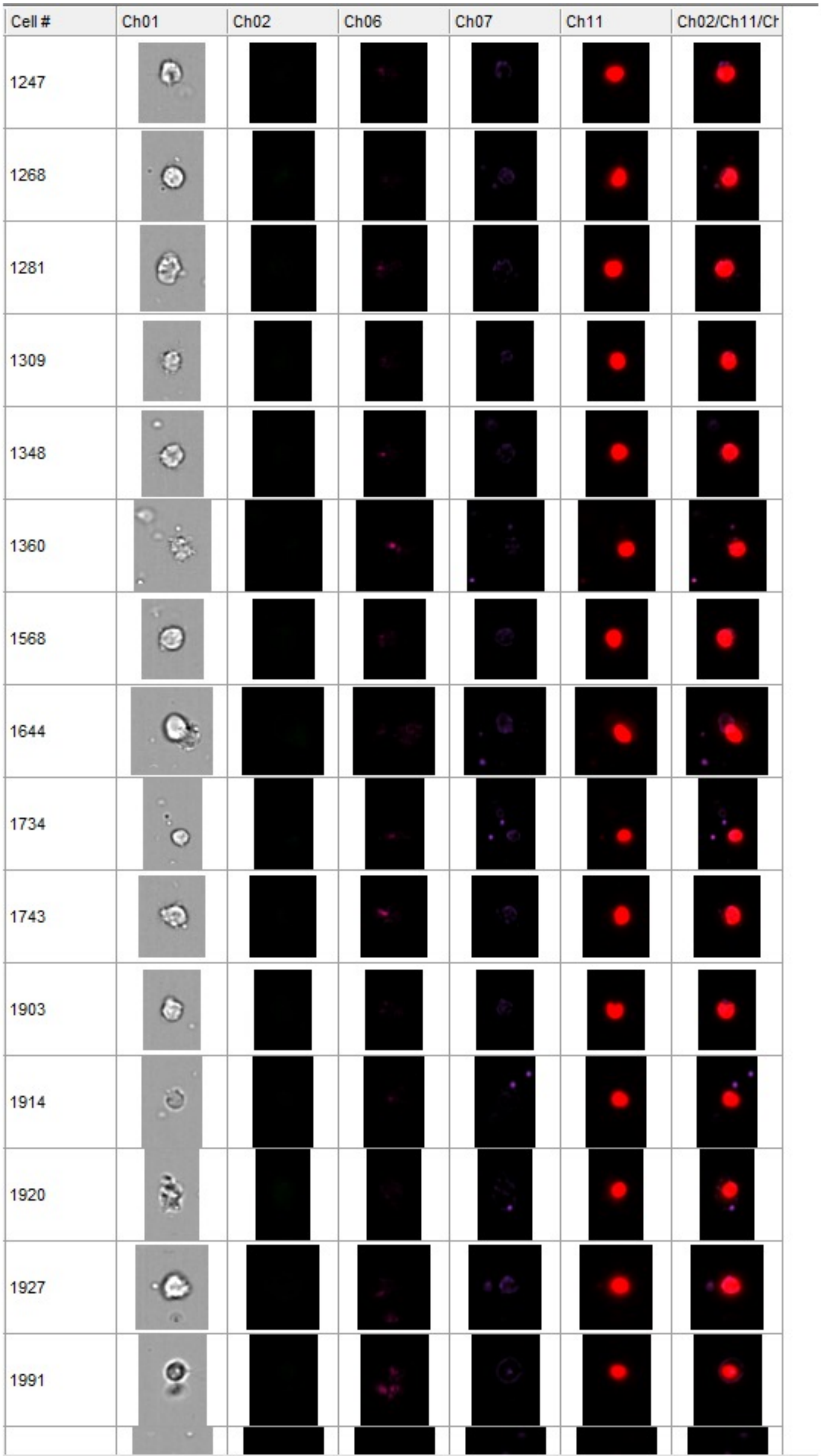
Cell #
Ch01
Ch02
Ch06
Ch07
Ch11
Ch02/Ch11/Ch
1247
1268
1281
1309
1348
1360
1568
1644
1734
1743
1903
1914
1920
1927
1991

*Pc*10_P

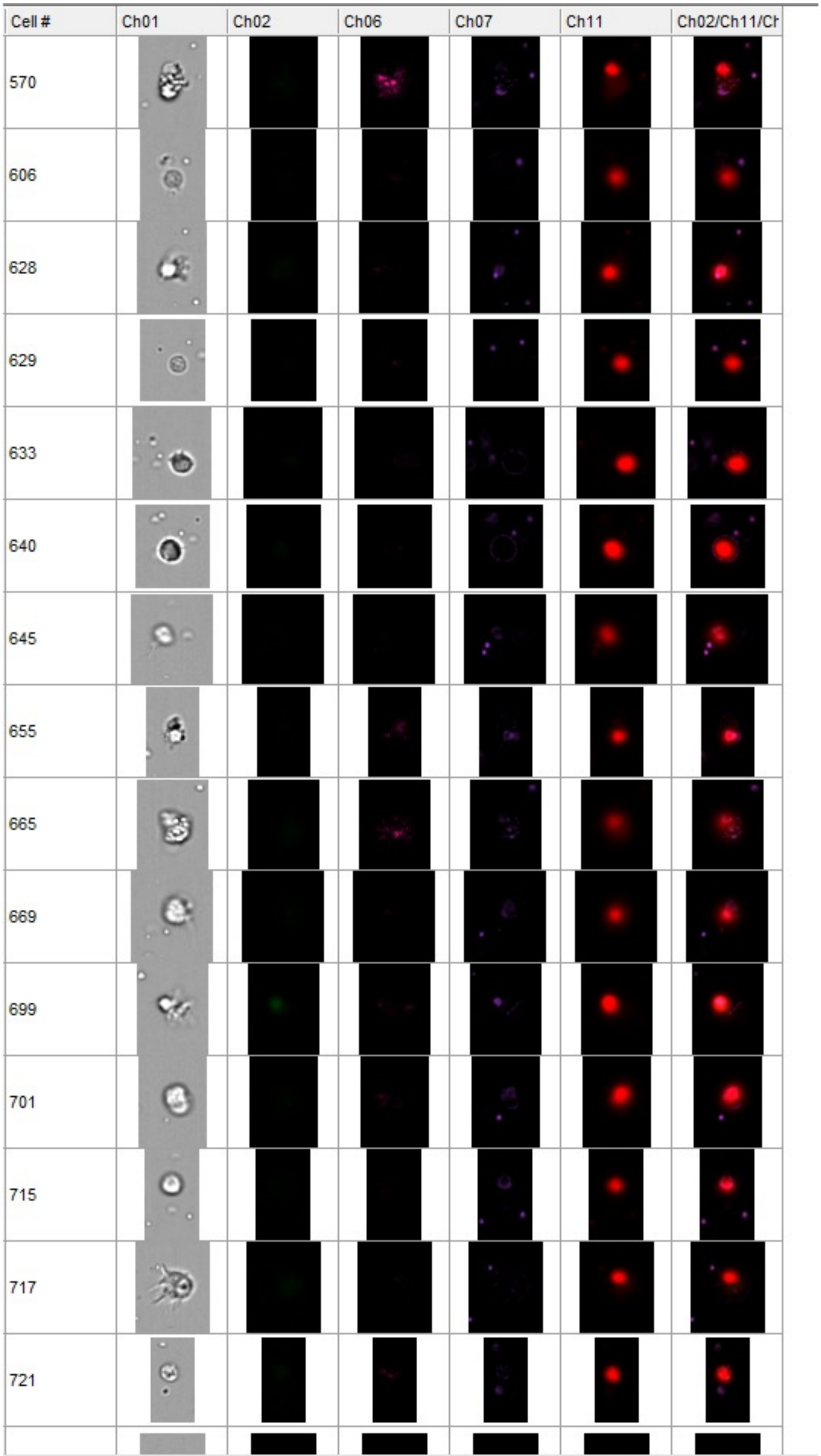
Cell #
Ch01
Ch02
Ch06
Ch07
Ch11
Ch02/Ch11/Ch
570
606
628
629
633
640
645
655
665
669
699
701
715
717
721

*Pc*11_P

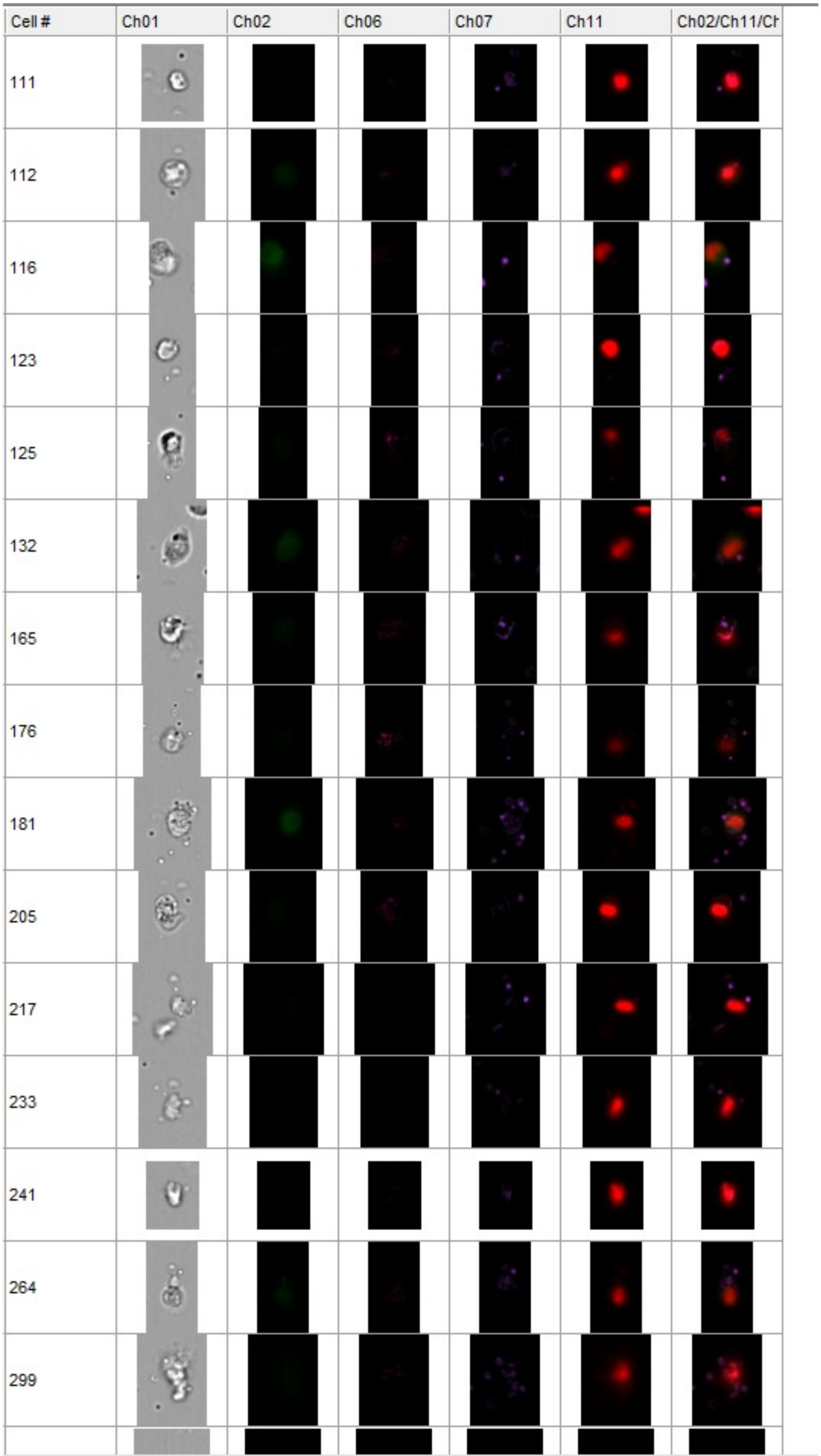

Cell #
Ch01
Ch02
Ch06
Ch07
Ch11
Ch02/Ch11/Ch
111
112
116
123
125
132
165
176
181
205
217
233
241
264
299

*Pc*12_P

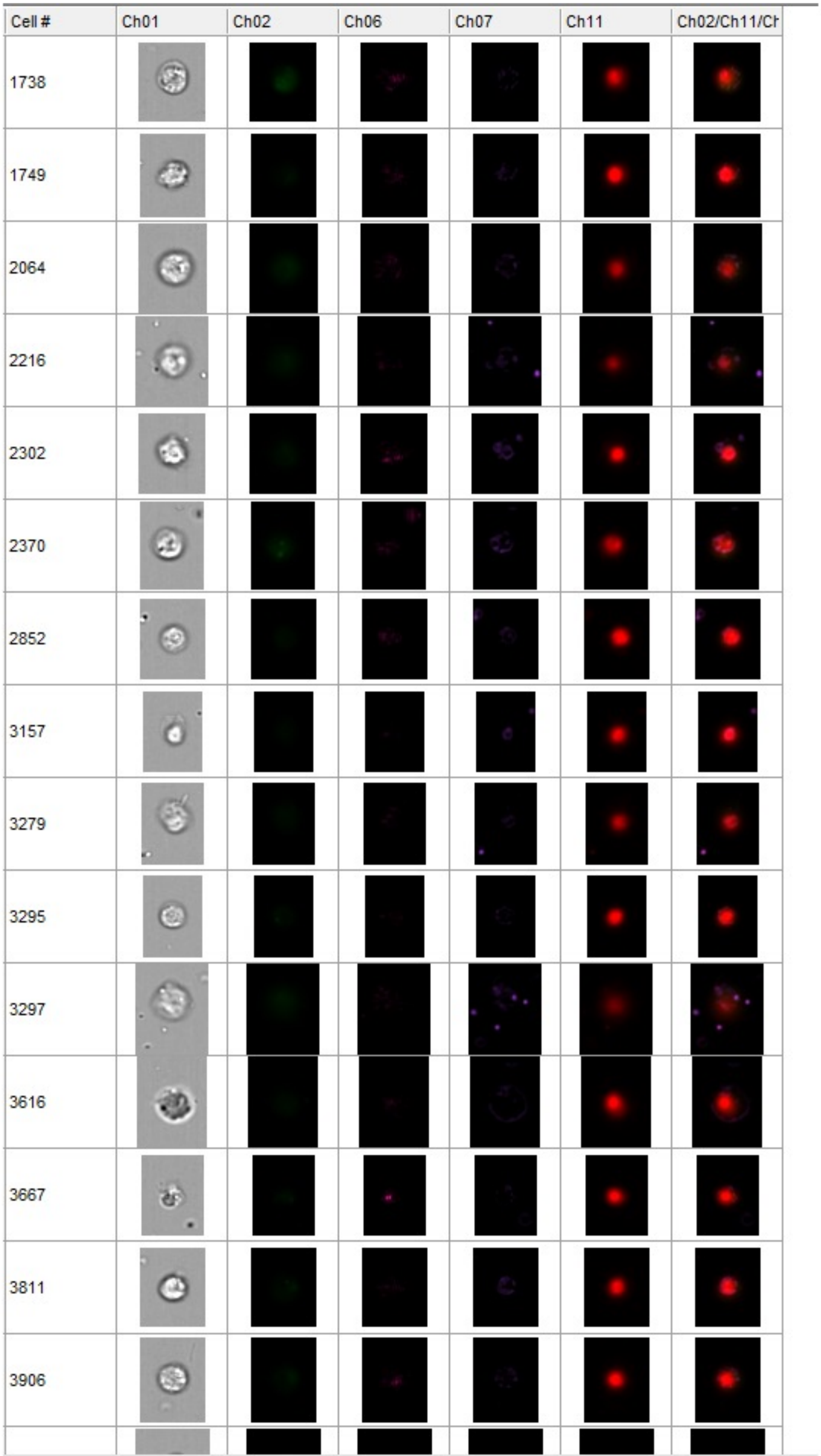
Cell #
Ch01
Ch02
Ch06
Ch07
Ch11
Ch02/Ch11/Ch
1738
1749
2064
2216
2302
2370
2852
3157
3279
3295
3297
3616
3667
3811
3906

*Pc*13_P

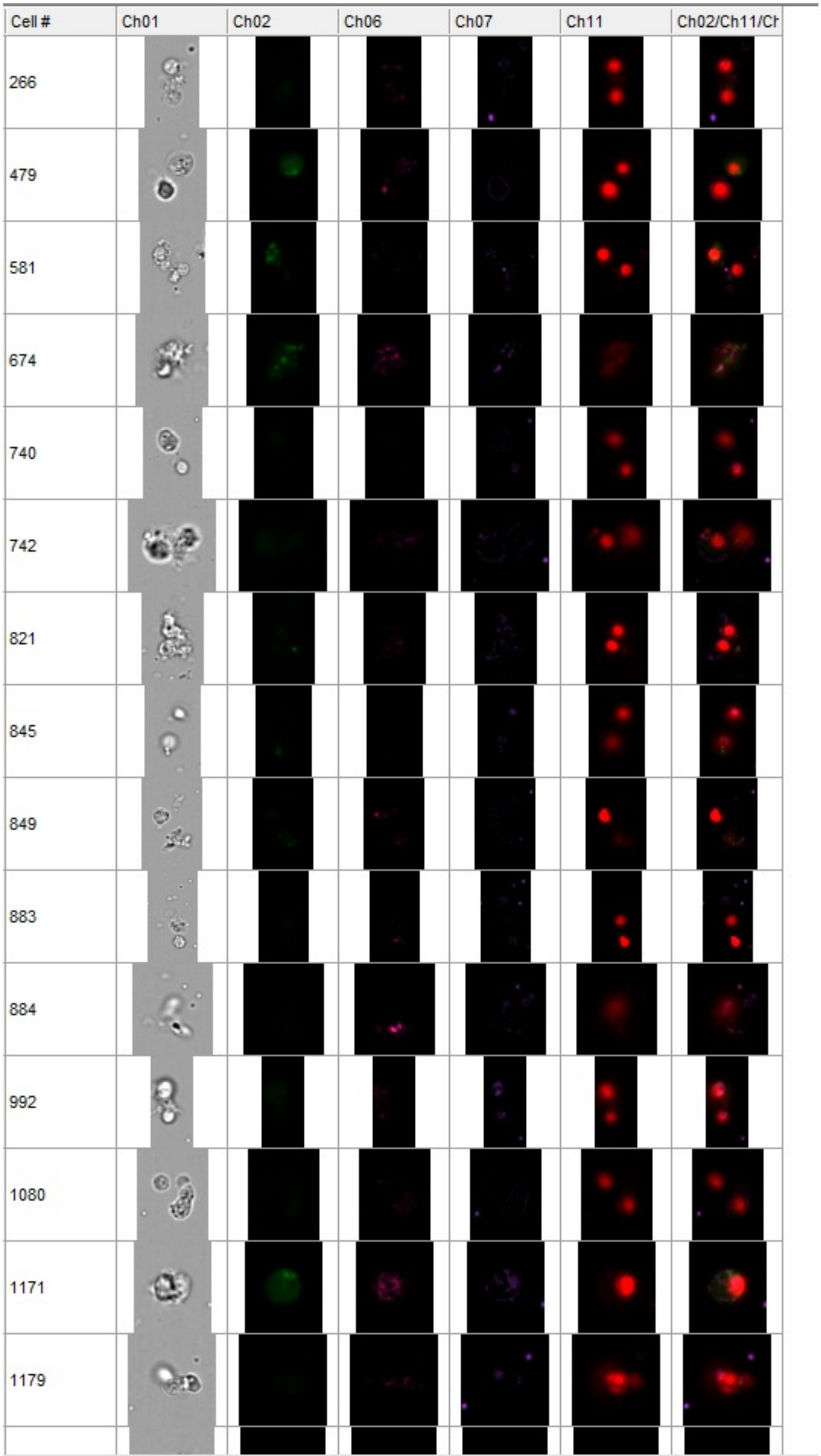

Cell #
Ch01
Ch02
Ch06
Ch07
Ch11
Ch02/Ch11/Ch
266
479
581
674
740
742
821
845
849
883
884
992
1080
1171
1179

*Pc*14_P

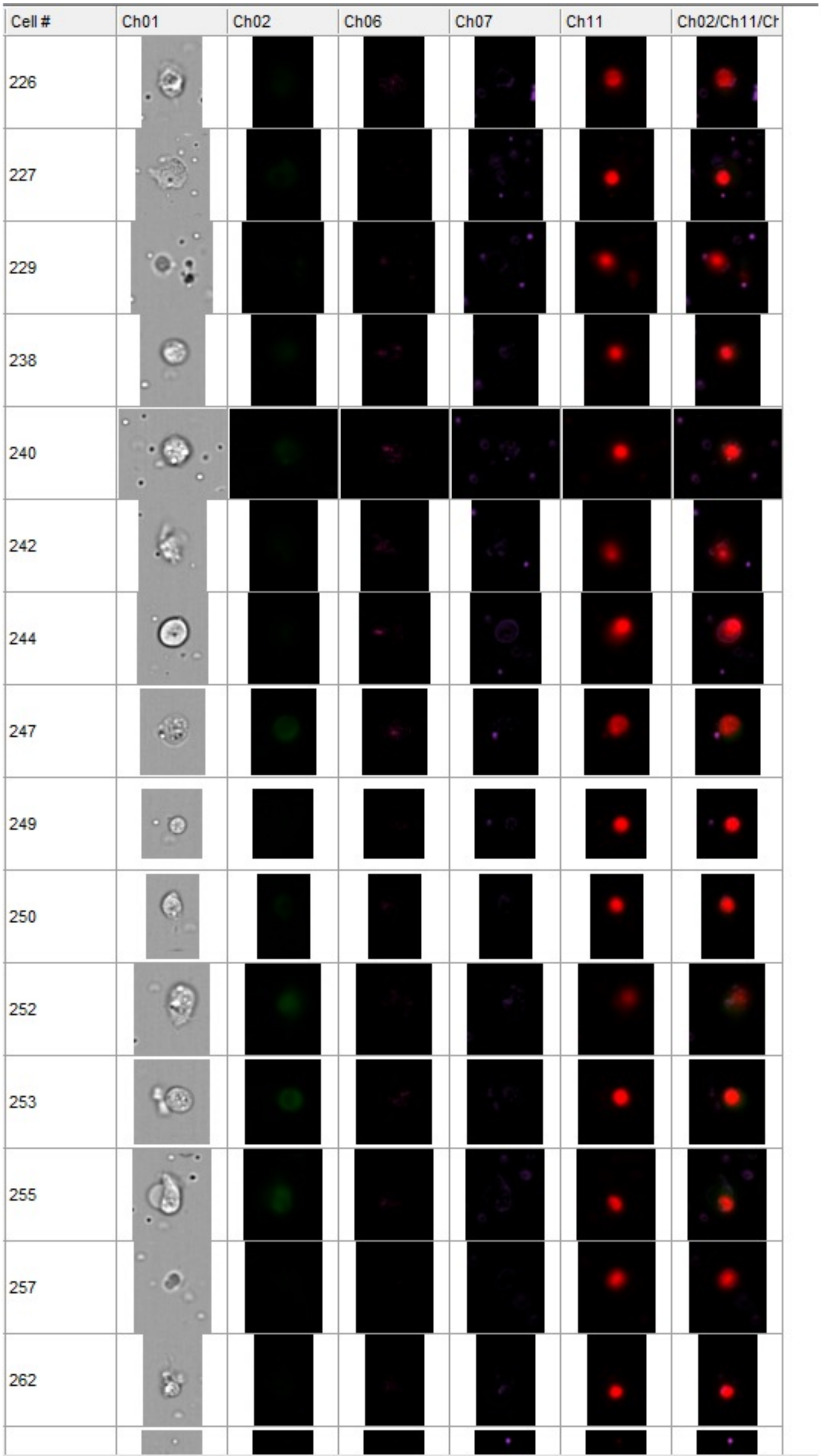
Cell #
Ch01
Ch02
Ch06
Ch07
Ch11
Ch02/Ch11/Ch
226
227
229
238
240
242
244
247
249
250
252
253
255
257
262

*Pc*15_P

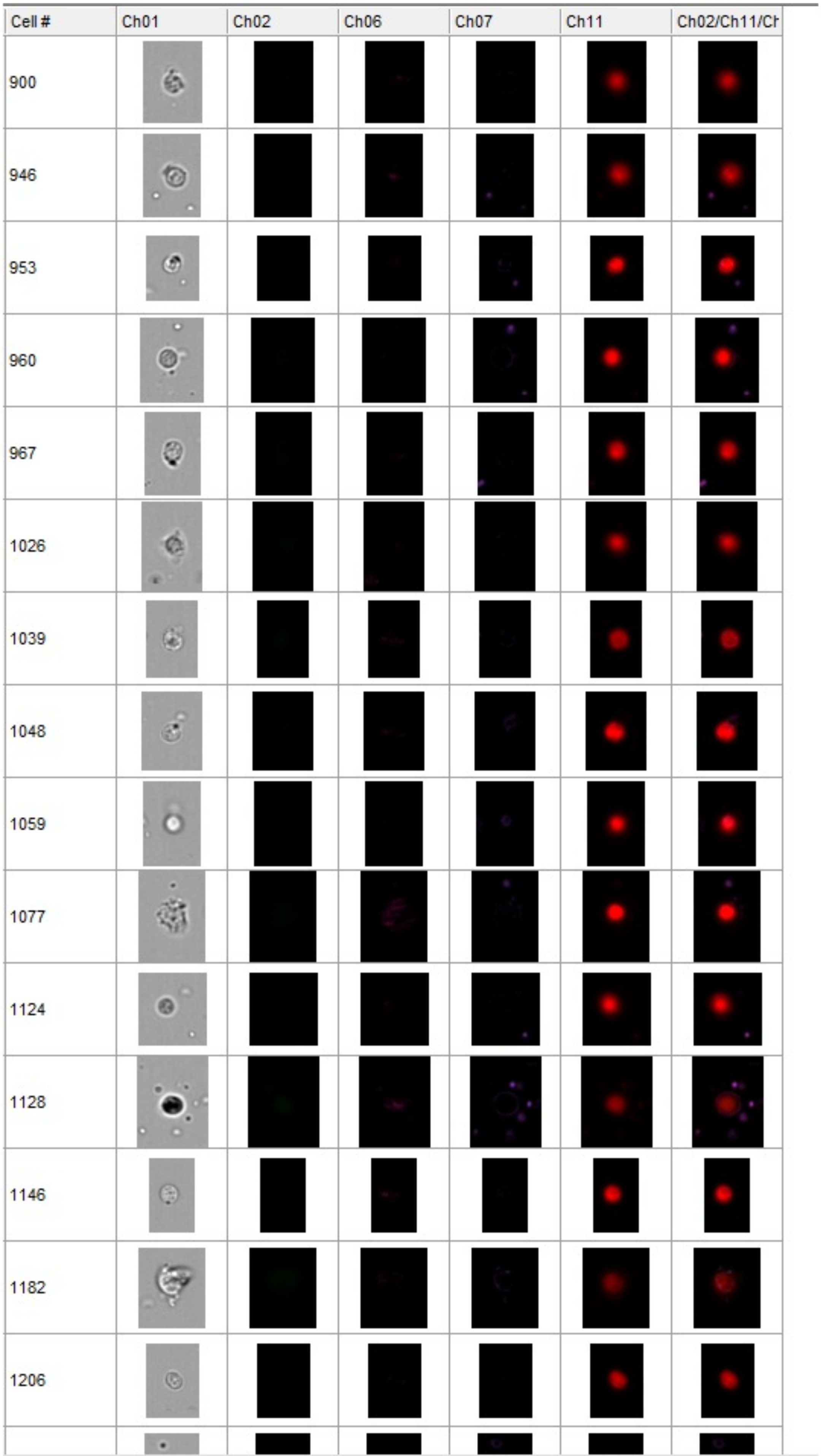
Cell #
Ch01
Ch02
Ch06
Ch07
Ch11
Ch02/Ch11/Ch
900
946
953
960
967
1026
1039
1048
1059
1077
1124
1128
1146
1182
1206

*Pc*16_P

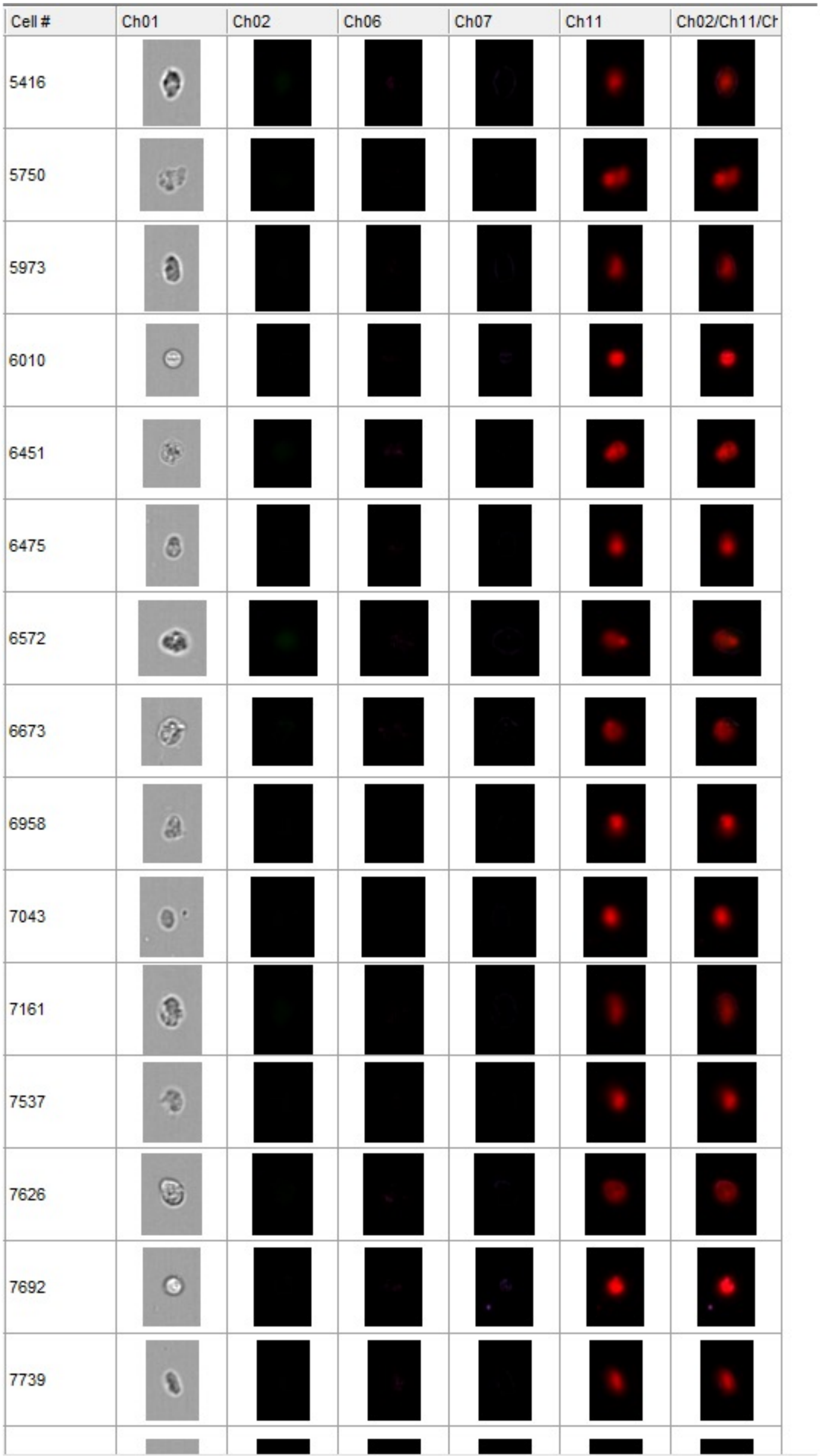

Cell #
Ch01
Ch02
Ch06
Ch07
Ch11
Ch02/Ch11/Ch
5416
5750
5973
6010
6451
6475
6572
6673
6958
7043
7161
7537
7626
7692
7739

*Pc*17_P   Professional phagocytes

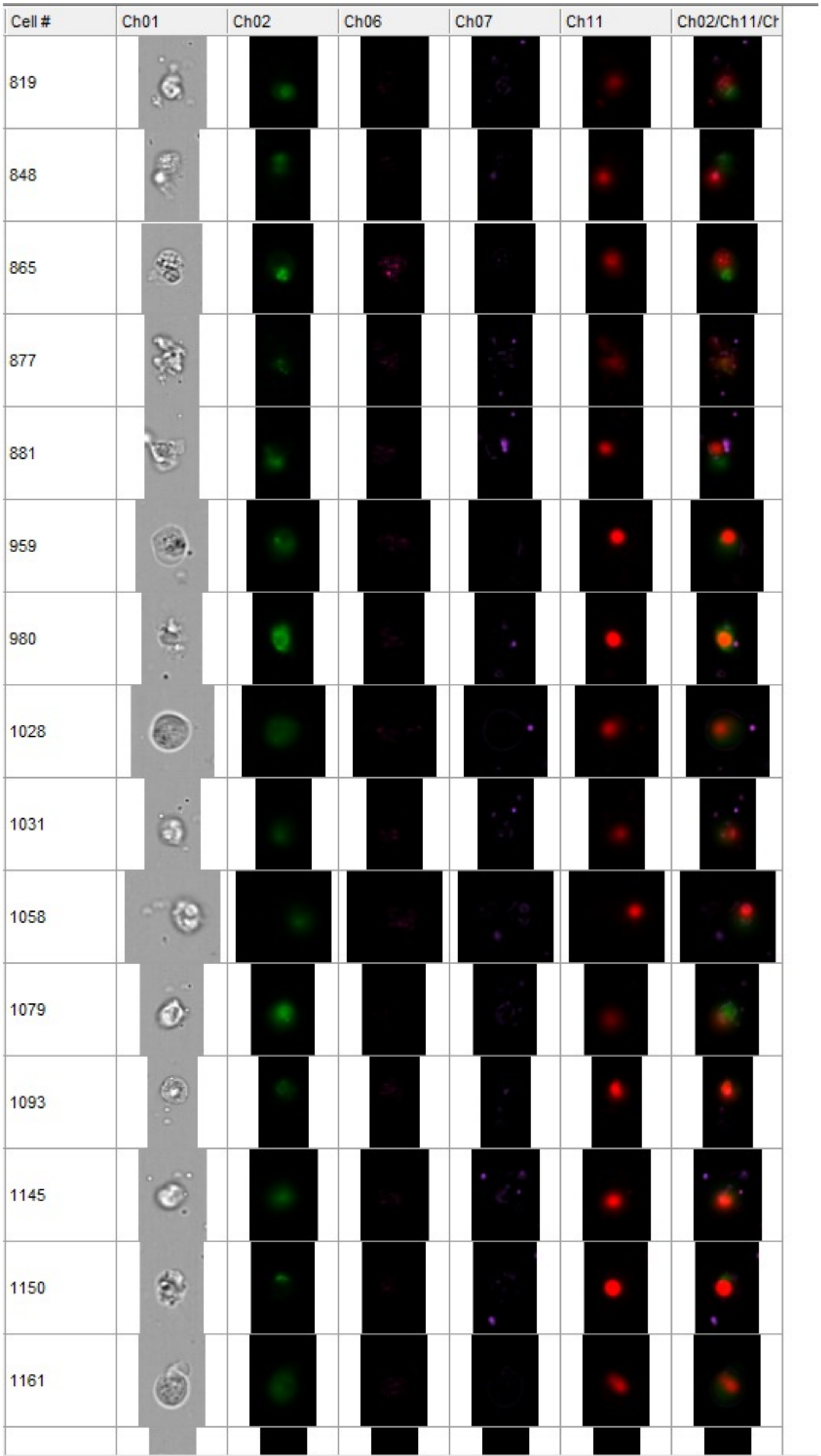

*Pc*18_P

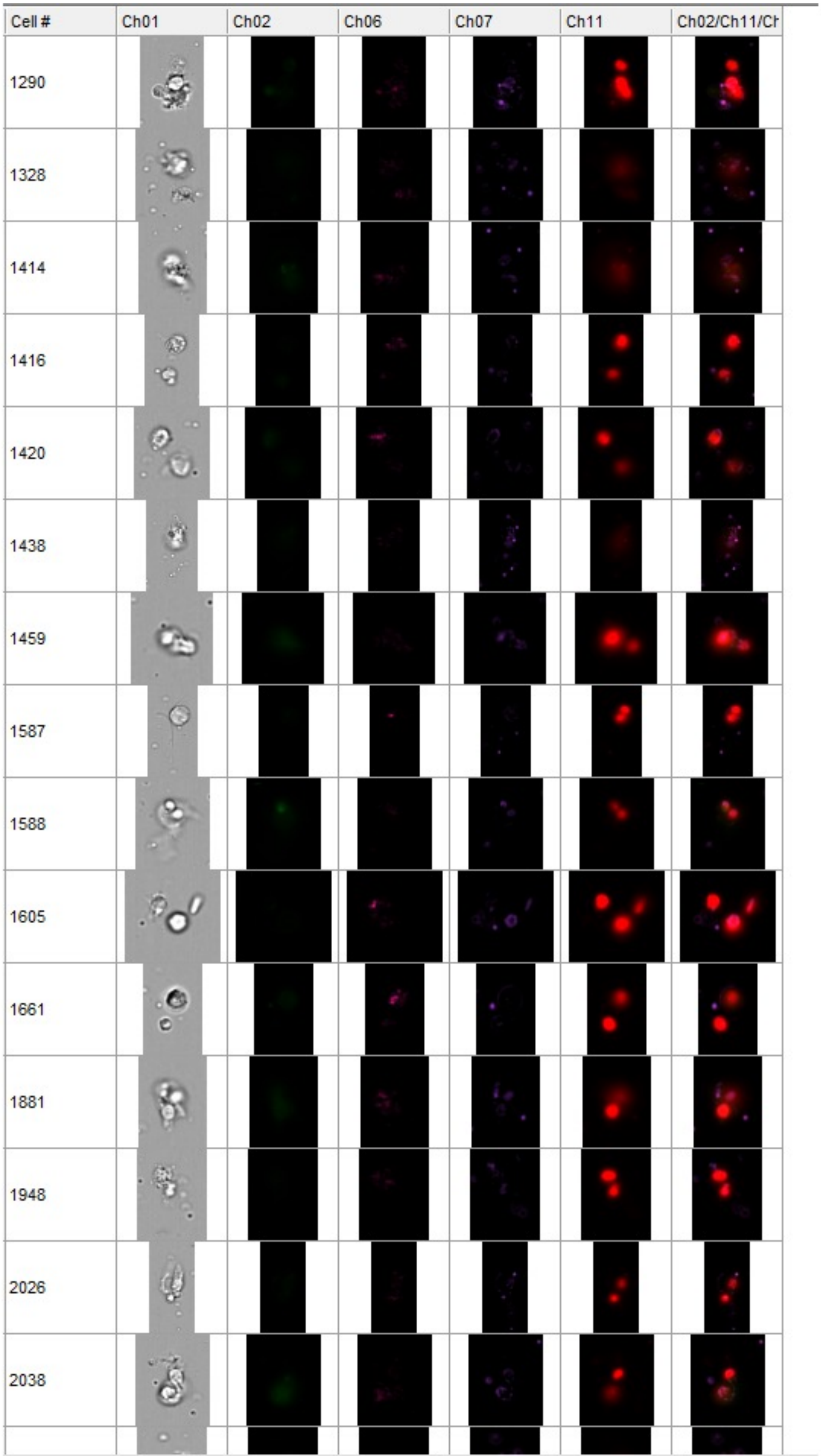
Cell #
Ch01
Ch02
Ch06
Ch07
Ch11
Ch02/Ch11/Ch
1290
1328
1414
1416
1420
1438
1459
1587
1588
1605
1661
1881
1948
2026
2038

*Pc*19_P

Cell #	Ch01	Ch02	Ch06	Ch07	Ch11	Ch02/Ch11/Ch
1289						
1311						
1366						
1404						
1466						
1592						
1779						
1838						
1902						
1917						
2132						
2189						
2213						
2625						
2886						

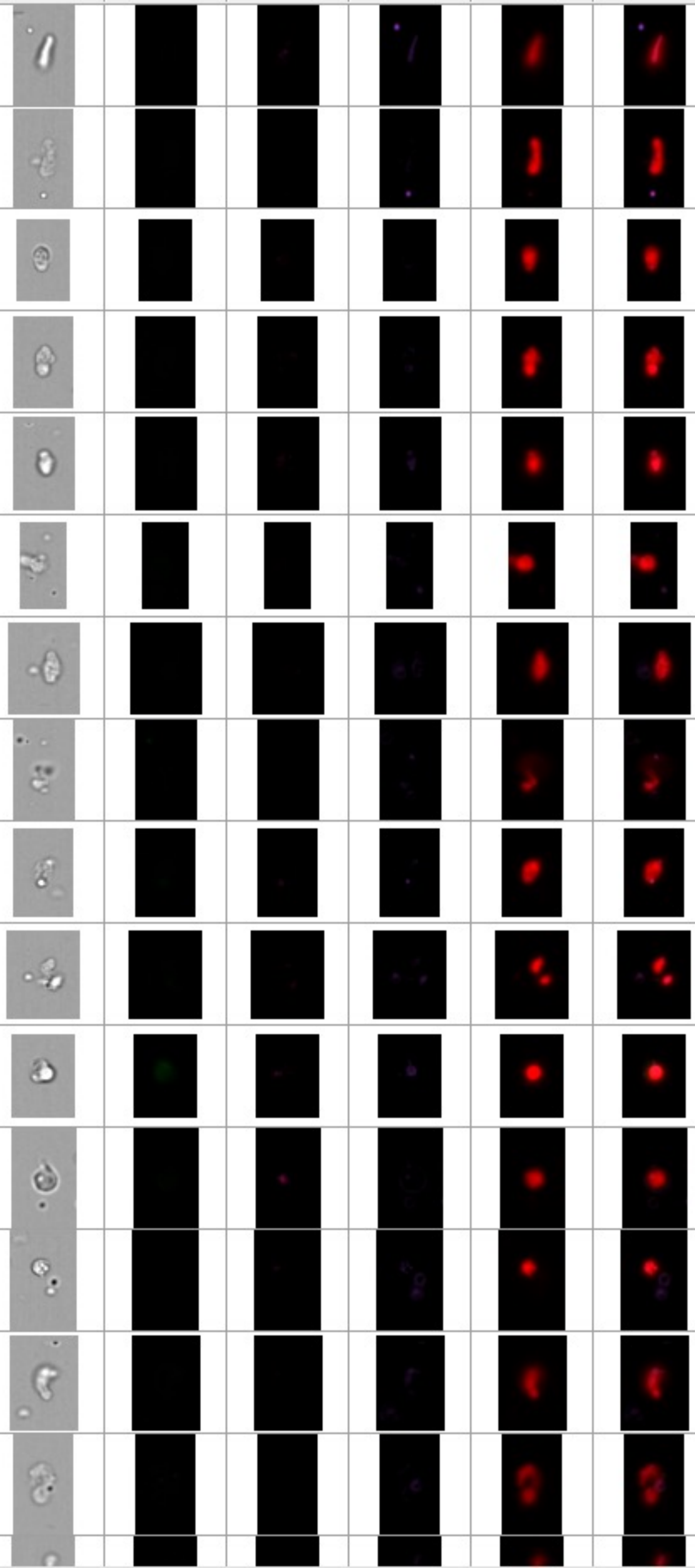

*Pc*20_P

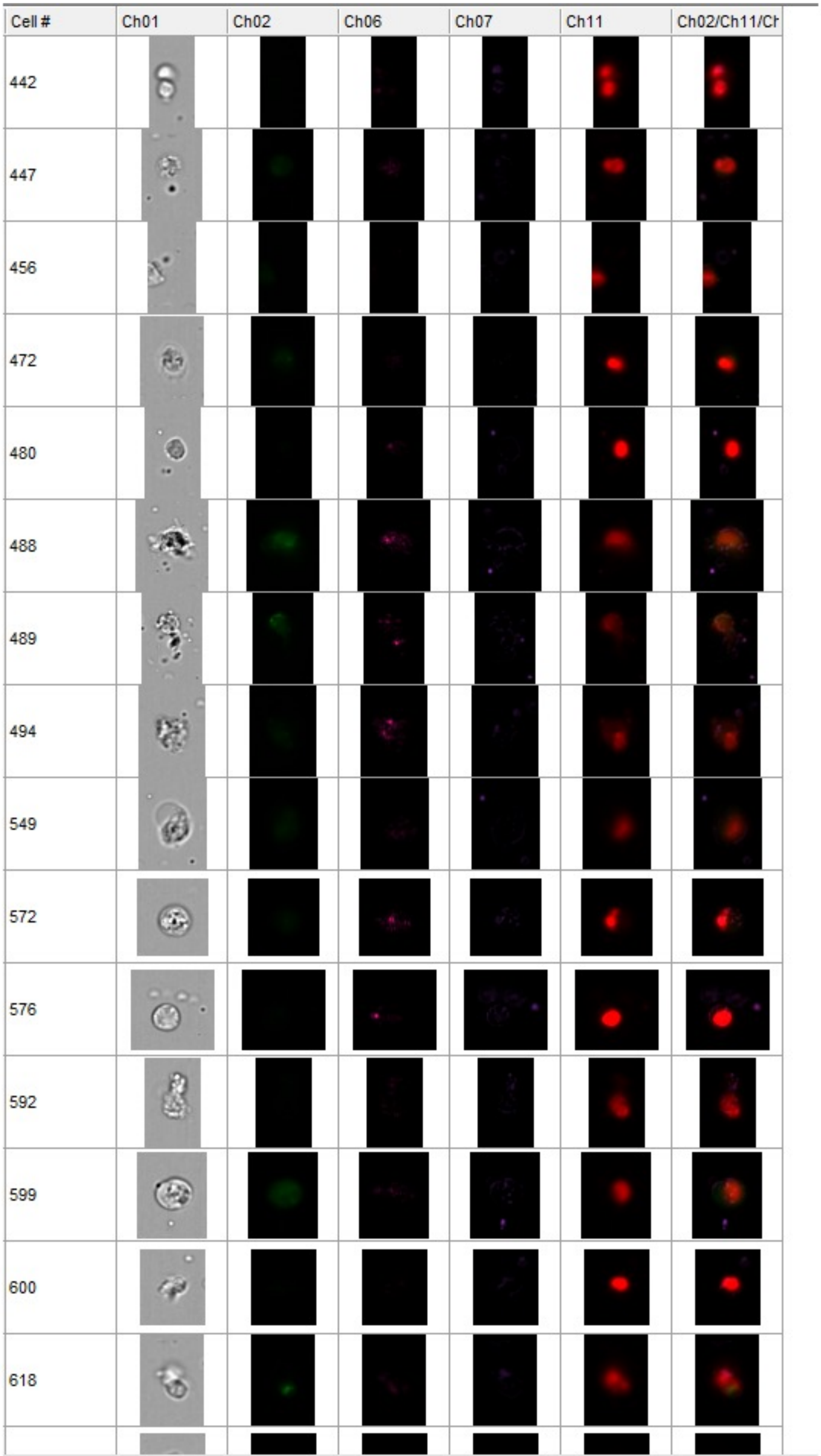
Cell #
Ch01
Ch02
Ch06
Ch07
Ch11
Ch02/Ch11/Ch
442
447
456
472
480
488
489
494
549
572
576
592
599
600
618
